# Catalytic Ring Opening of Epoxides by Metal Triflates: Influence of a Pending Olefin

**DOI:** 10.3390/molecules31162828

**Published:** 2026-08-13

**Authors:** Matthieu Jorandon, Nadia Patino, Elisabet Duñach, Mohamed Mehiri

**Affiliations:** Institut de Chimie de Nice, Université Côte d’Azur, CNRS UMR 7272, 06108 Nice, France; matthieu.jorandon@univ-cotedazur.fr (M.J.); nadia.patino@univ-cotedazur.fr (N.P.); elisabet.dunach-clinet@univ-cotedazur.fr (E.D.)

**Keywords:** epoxides, metal triflates, epoxyolefin cyclization, epoxide isomerization, bismuth (III) triflate

## Abstract

Lewis acid-catalyzed epoxide openings efficiently promote intramolecular cyclizations forming complex oxygenated frameworks. Herein, we investigated the reactivity of epoxyolefins under catalytic conditions using various metal triflates. The study focuses on the competition between epoxide isomerization and intramolecular cyclization involving a pendant olefin. Screening of several metal triflates revealed that Lewis superacids, particularly Bi(OTf)_3_, promote efficient transformations at room temperature with low catalyst loadings (1 mol%). Structural variation in the substrates significantly influenced product distribution likely through coordination effects, impacting cyclization efficiency with higher yields observed for non-chelating epoxyolefins. Substrates derived from geraniol underwent selective cyclization to *cis*-cyclic alcohols and bicyclic ethers in good yields, with stereochemical outcomes consistent with the Eschenmoser model. Reaction conditions such as temperature and catalyst loading further modulated selectivity, allowing control over product distribution. These results highlight the potential of metal triflates as versatile and efficient catalysts for the stereoselective intramolecular cyclization of epoxyolefins, offering access to cyclic frameworks under mild conditions.

## 1. Introduction

Activation and ring-opening of epoxides by Lewis acids is a process that can lead to epoxide polymerization or isomerization [1,2]. Simultaneous activation of epoxides and olefins enables biomimetic cyclizations providing oxygenated natural products [3,4,5]. In particular, the biosynthesis of triterpenes from oxidosqualene affords a large structural variety of triterpenols and other metabolites, with important biological activities [6,7]. Thus, the major advantage of this synthetic approach lies in its ability to generate a high level of molecular complexity in a single step starting from acyclic substrates.

**Scheme 1:**
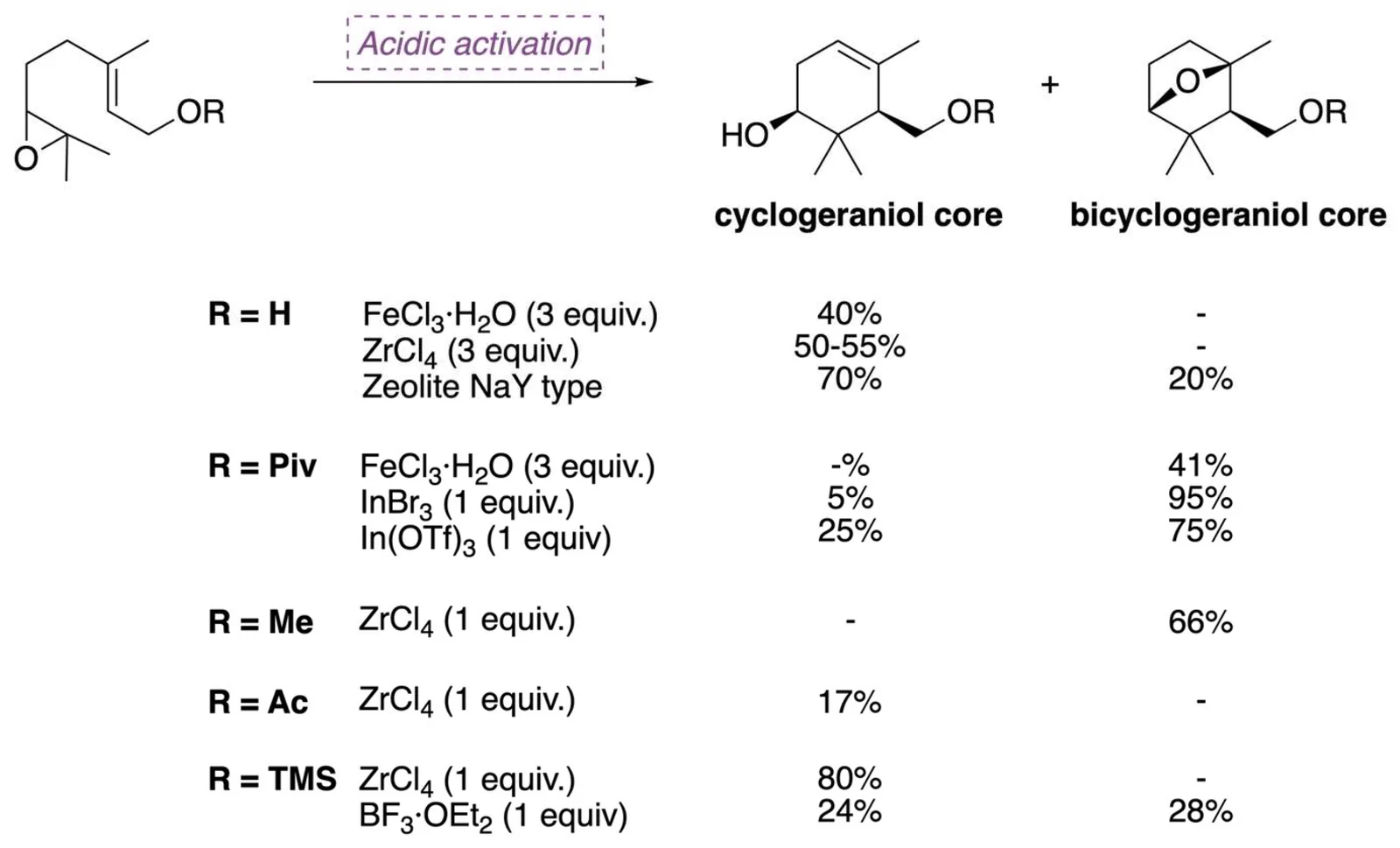
Overview of significant previous reports concerning the cyclization of 6,7-epoxygeraniol derivatives [8,9,10,11,12,13,14,15,16,17,18,19,20,21,22].

Previous Lewis acid-mediated cyclizations of epoxyolefins typically required stoichiometric conditions, using reagents such as BF_3_.OEt_2_ [8], Sn(IV) [9,10,11], MeAl(II) [12,13,14,15], Zr(IV) [16,17], Fe(III) [18], In(III) [19], or Bi(III) halides [20] (Scheme 1). As an example, InBr_3_ has been used for the cyclization of geranyl pivaloyl oxide by Graham A. E. and co-workers [1]. Furthermore, Mohan’s group has described the catalysis by some metal triflates and determined that a catalytic amount of Bi(OTf)_3_ was efficient to promote the cyclization of epoxygeraniol [20]. Additionally, the use of NaY zeolites has also been reported as an interesting alternative [21,22]. In most promising examples reported under stoichiometric or excess conditions, attempts have generally been made to reduce the reagent loading to achieve catalytic turnover. However, this generally resulted in a drastic decrease in cyclization rate, mainly due to competing epoxide isomerization. It is noteworthy that a recent example has reported the formation of cyclic ether, including eight-membered ring cyclic ethers, from the intermolecular cyclization (i.e., [6+2] cycloaddition) of epoxide derivatives and vinyl oxetane [23].

On this basis, we sought to develop a catalytic methodology centered on more active Lewis acids such as metal triflates to promote the cyclization of diverse epoxyolefins, including the 6,7-epoxygeraniol scaffold. Such an approach could provide a catalytic entry to the synthesis of natural products featuring cyclogeraniol-type cores, such as damascone or apotrisporin E [24].

It should be kept in mind that, in the presence of Lewis acids, epoxides may undergo, besides polymerization, ring opening to ketones or allylic alcohols, as well as the Meinwald rearrangement to aldehydes [2]. It was recently reported that acetals bearing pendant olefins were efficiently cyclized with only 1 mol% of Bi(OTf)_3_ [25]. Herein, we report the reactivity of various epoxyolefins under highly catalytic Lewis acid conditions. The extension of this approach to ether-containing epoxyolefins would provide a new route to access cyclic ether architectures. In this context, epoxyolefins which do or do not contain an ether function were examined, as well as the reactivity of a series of monoterpene epoxide derivatives.

## 2. Results and Discussion

### 2.1. Ether Epoxyolefins

**Scheme 2:**
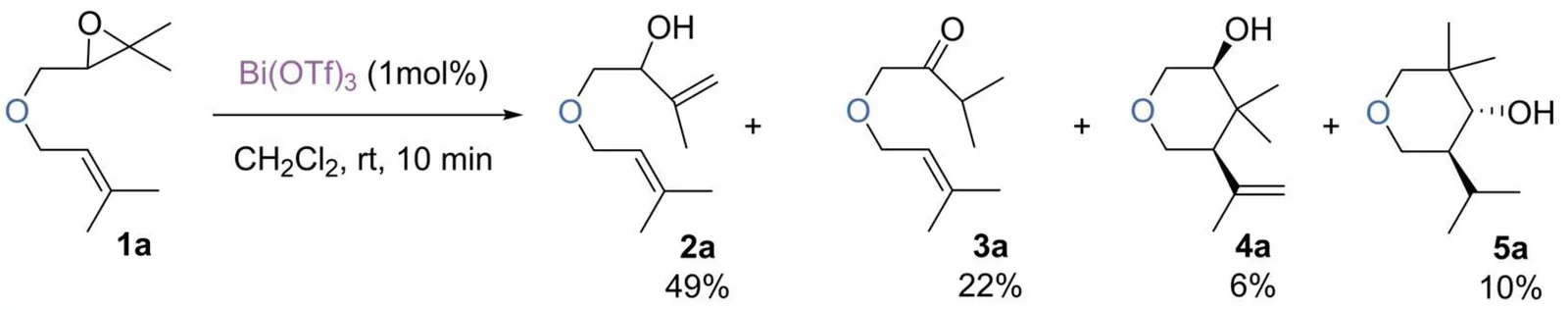
Reactivity of the model substrate **1a** in presence of 1 mol% of Bi(OTf)_3_ (GCFID yields).

The monoepoxide of 3-methyl-1-[(3-methylbut-2-en-1-yl)oxy]but-2-ene, **1a**, was prepared and used as a model substrate for its simplicity and ease of preparation to investigate its reactivity in metal triflate-catalyzed transformations. All reactions were monitored by GCMS and GCFID analysis. Treatment of **1a** with 1 mol% of Bi(OTf)_3_ in dichloromethane (0.03 M) at room temperature led to its complete conversion within 10 min. As shown in Scheme 2, four compounds were isolated: allylic alcohol **2a** (49%, GCFID yield), ketone **3a** (22%, GCFID yield), and two cyclized alcohols *cis*-**4a** (6%, GCFID yield) and *trans*-**5a** (10%, GCFID yield). The relative configurations of **4a** and **5a** were determined by ^1^H–^1^H NOESY NMR experiments.

**Scheme 3:**
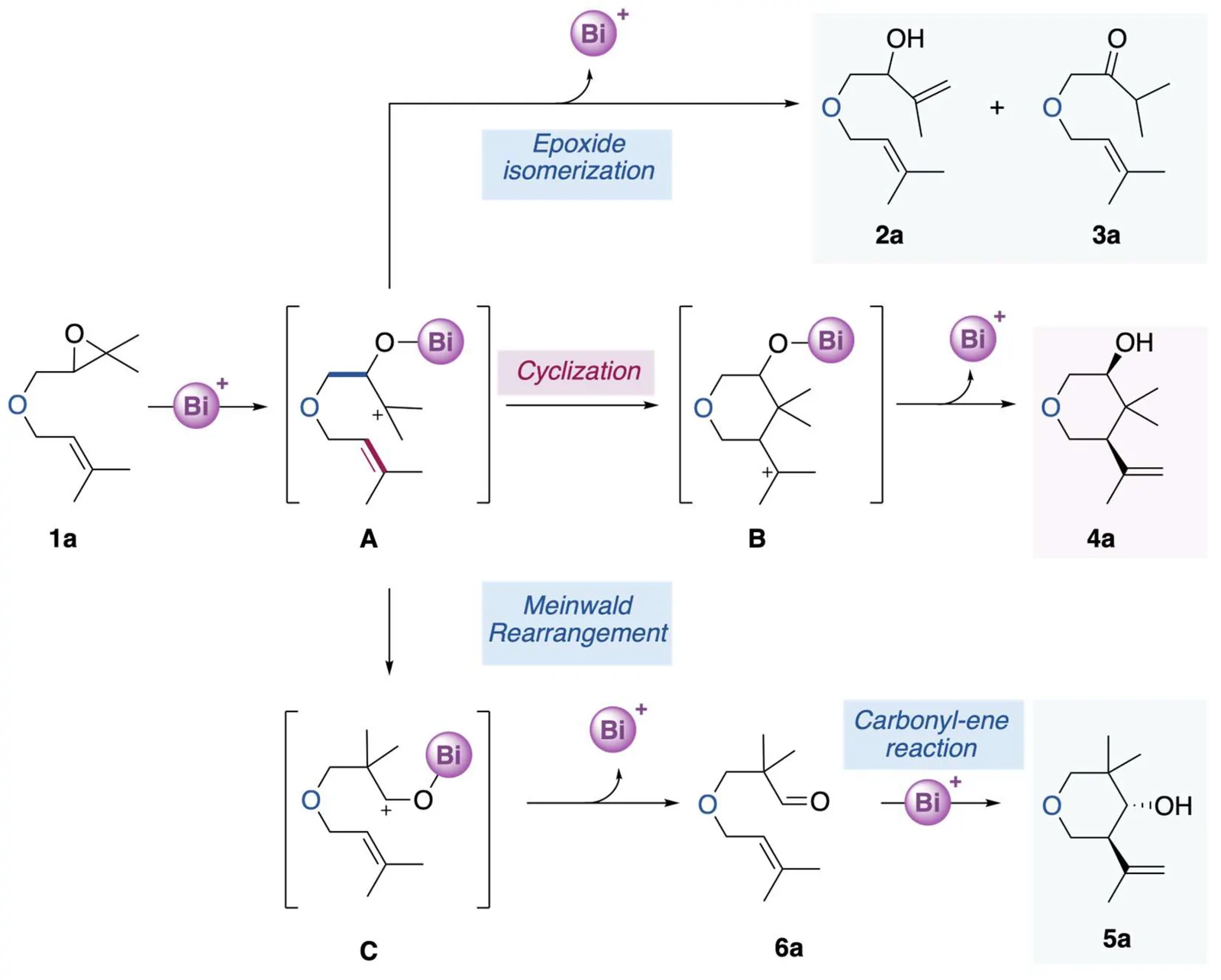
Proposed mechanism for the formation of **2a**–**6a**.

The formation of compounds **2a**–**5a** is consistent with a cationic mechanism involving different reaction pathways, as proposed in Scheme 3. However, this mechanistic proposal remains speculative, as it is not supported by direct experimental evidence, and further experiments (i.e., control, trapping, or kinetic studies) might help to substantiate it. Whereas **2a** and **3a** arise from the epoxide isomerization, compound **4a** may be derived from a first activation of the epoxide followed by the addition of the olefin to the intermediate carbocation **A**, which cyclizes to intermediate **B**. In addition, formation of the cyclized product **5a** may proceed from a first Meinwald epoxide rearrangement to the aldehyde intermediate **6a** *via* intermediate **C**. Moreover, the conversion of **6a** into **5a** was observed in certain reactions through GCMS monitoring (e.g., at lower temperature), which supports the conclusion that cyclized product **5a** is not formed directly from **1a** but rather arises *via* an intramolecular carbonyl-ene type cyclization from **6a** [26].

The reactivity of **1a** was further investigated using several additional catalysts at only 1 mol%, as summarized in Table 1.

All the assessed metal triflate catalysts demonstrated pronounced reactivity, leading to complete conversion of **1a** within 10 min (entries 1–5). In contrast, significantly lower activities were observed with BiCl_3_, BF_3_.OEt_2_, and *p*-toluenesulfonic acid (entries 6–8). In all reactions catalyzed by metal triflates, alcohol **2a** was identified as the main compound. Only a limited formation of cyclized product **4a**, through the direct olefin addition was observed with Bi(OTf)_3_ and Fe(OTf)_3_ (entries 1, 2), whereas aldehyde **6a** was preferentially formed in the presence of Al(OTf)_3_, Sc(OTf)_3,_ and In(OTf)_3_ (entries 3–5). This may suggest that these Lewis acids are less effective at coordinating the aldehyde functionality of **6a**, thereby disfavoring activation toward the carbonyl-ene process required for cyclization to **5a**. Nevertheless, Lewis acidity may not be the only parameter governing the product distribution. Differences in the size of the metal center, stability or rate of each step involving the catalyst–substrate complex may also play a role. Moreover, some commercially available metal triflates may contain some residual water, thus reducing their acidity.

The use of different triflates enabled us to examine the influence of the metal cation, whereas using BiCl_3_ was employed to probe the effect of the anion. As chloride is a more coordinating and less electron-withdrawing anion than triflate, it decreases the Lewis acidity of the metal center, making BiCl_3_ a significantly weaker Lewis acid than Bi(OTf)_3_. Consistently, no conversion was observed with BiCl_3_ (entry 6), indicating that a significant Lewis acidity is required to efficiently activate the epoxide under catalytic conditions. In addition, the weakly coordinating triflate anion may facilitate the catalytic cycle by allowing substrate coordination while stabilizing cationic intermediates [27].

**Scheme 4:**
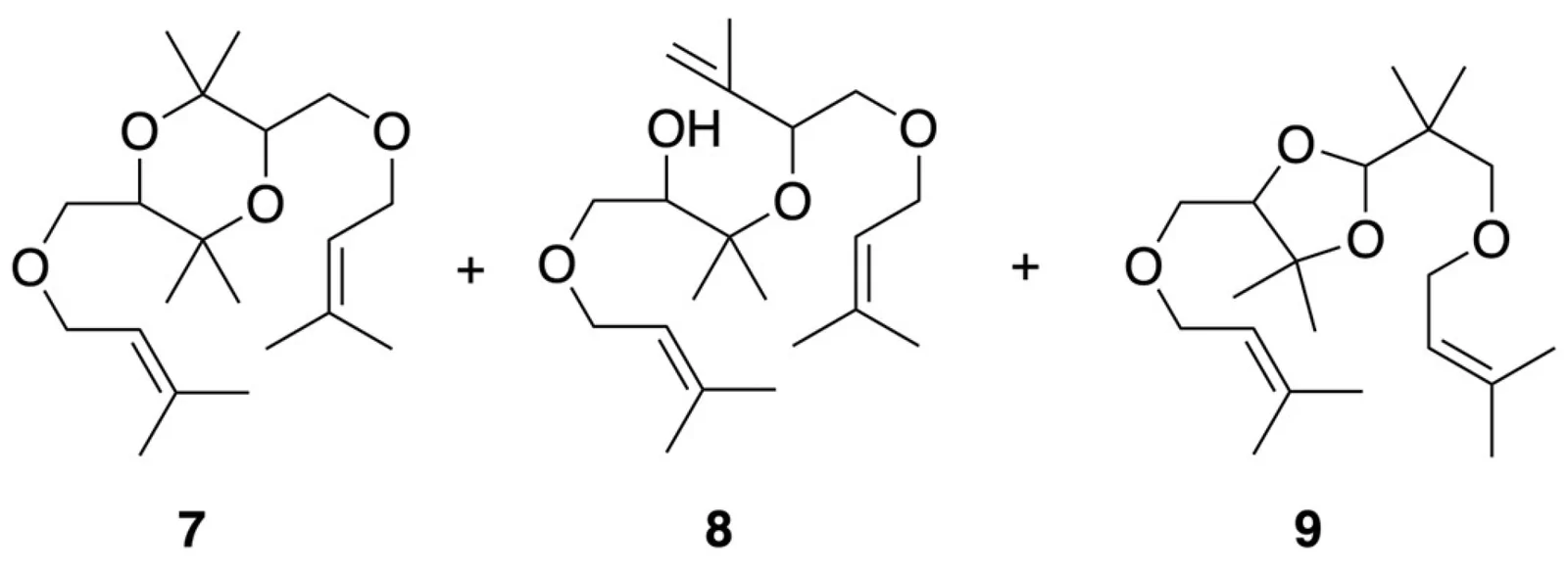
Structures of the dimerization products derived from **1a**: **7**, **8** and **9**.

Interestingly, BF_3_·OEt_2_ led to poor conversion (entry 7), with no further reaction progress observed after 2 h. Moreover, over 32% of the reaction mixture consisted of dimerization products (Scheme 4). The formation of dimerization products with BF_3_·OEt_2_ likely arises from epoxide activation, followed by trapping by a second epoxide molecule, as in the epoxide polymerization, without involving the olefin.

Exposure to a strong Brønsted acid (PTSA) afforded only **2a** (entry 8) with low conversion, this product being an epoxide isomerization product. This might indicate that activation by simple protonation does not enable the same mechanistic pathway as coordination to a metal center. As no significant activation toward cyclization was observed under these protic conditions, this suggests that Brønsted acids are not able to efficiently activate both the epoxide and the olefin in this system.

Moreover, the influence of temperature on the product distribution was examined. Epoxyolefin **1a** was treated at 0, 20, 40 and 80 °C in reactions catalyzed by Bi(OTf)_3_. In all cases, alcohol **2a** remained the predominant compound. Interestingly, the direct cyclization to **4a** increased with the reaction temperature. At 80 °C, in 1,2-dichloroethane, after 10 min of catalytic amount of Bi(OTf)_3_ reaction, **4a** was obtained in 17% yield together with **5a**, detected in 44% yield (GCFID analysis). However, several by-products underwent thermal degradation.

**Scheme 5:**
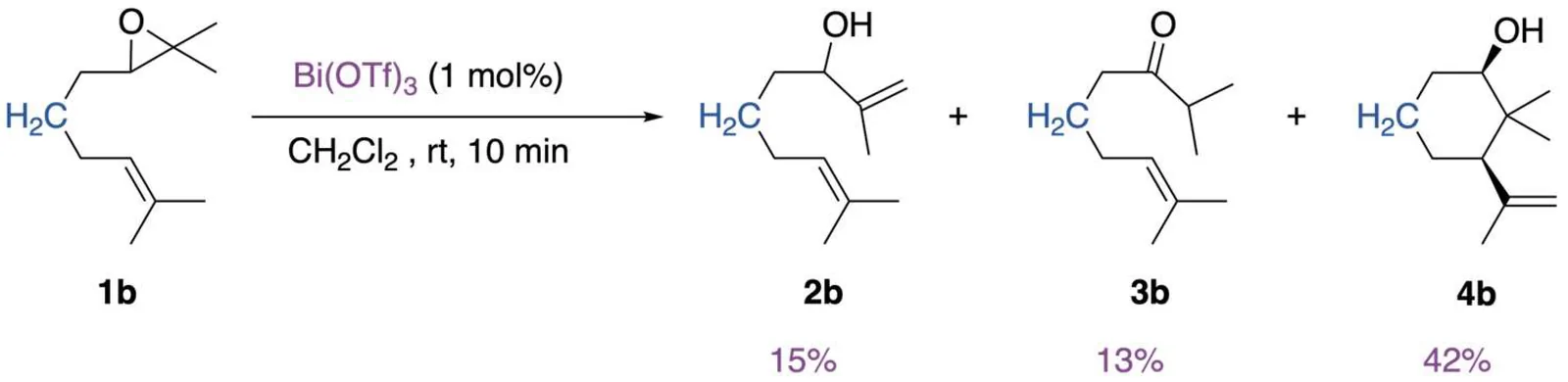
Reactivity of **1b** in presence of 1 mol% of Bi(OTf)_3_.

The chain oxygen in **1a** likely coordinates to the Lewis acid, reducing direct cyclization efficiency to **4a**. In order to test this hypothesis, compound 2,8-dimethyl-2,7-nonadiene was prepared and oxidized to the analogous epoxyolefin **1b**. The reactivity of **1b** under the conditions of entry 1 (Table 1) afforded the expected cyclized **4b** as the main reaction product, in 42% yield (Scheme 5).

**Scheme 6:**
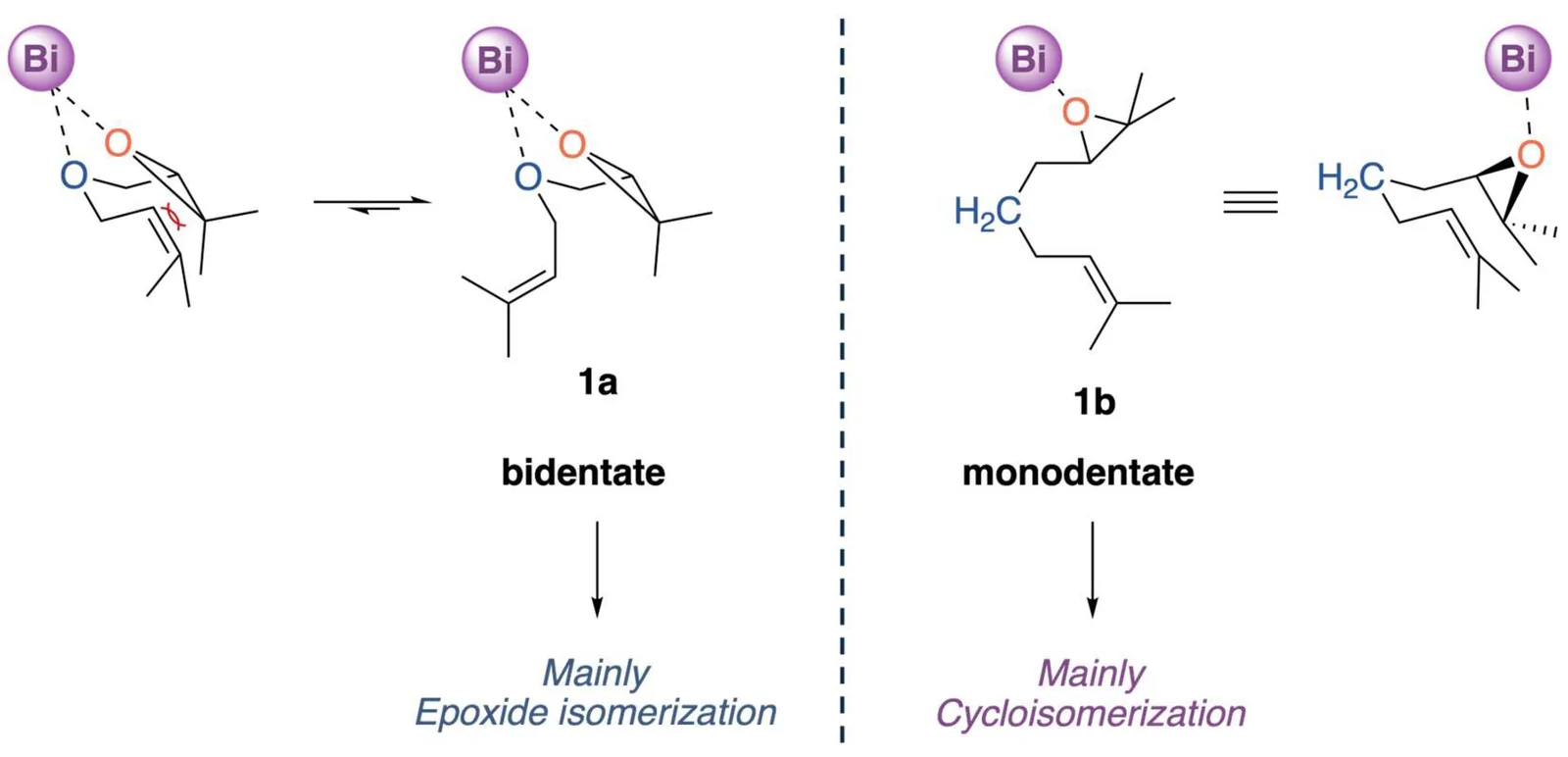
Influence of the Bi(OTf)_3_ coordination to **1a** or **1b** that influences the reaction outcome.

The oxo group in **1a** appears to strongly influence the reaction outcome compared to **1b**. Regarding the direct cyclization with the pendant olefin with 1 mol% of Bi(OTf)_3_, compound **4a** was obtained in only 6% yield, whereas under identical conditions, compound **4b** became the major product, obtained in 42% yield. As illustrated in Scheme 6, a change in coordination to the Bi(III) catalyst between **1a** and **1b** might explain this result.

Bidentate coordination, arising from chelation between the epoxide and the oxygen atom within the carbon chain, induces a folded and relatively stable conformation. Due to steric hindrance, this conformational constraint may both reduce the accessibility of the electrophilic carbon of the epoxide and locate the olefin farther from the reactive center, thereby disfavoring intramolecular addition. Therefore, it may promote competing epoxide opening and isomerization pathways. In contrast, under monodentate coordination, only the epoxide is activated while the rest of the substrate remains conformationally flexible and can adopt conformations that bring the olefin closer to the reactive center, thereby facilitating cyclization. This hypothesis remains speculative and would require theoretical calculations for validation.

### 2.2. Monoterpene Derivatives

**Scheme 7:**
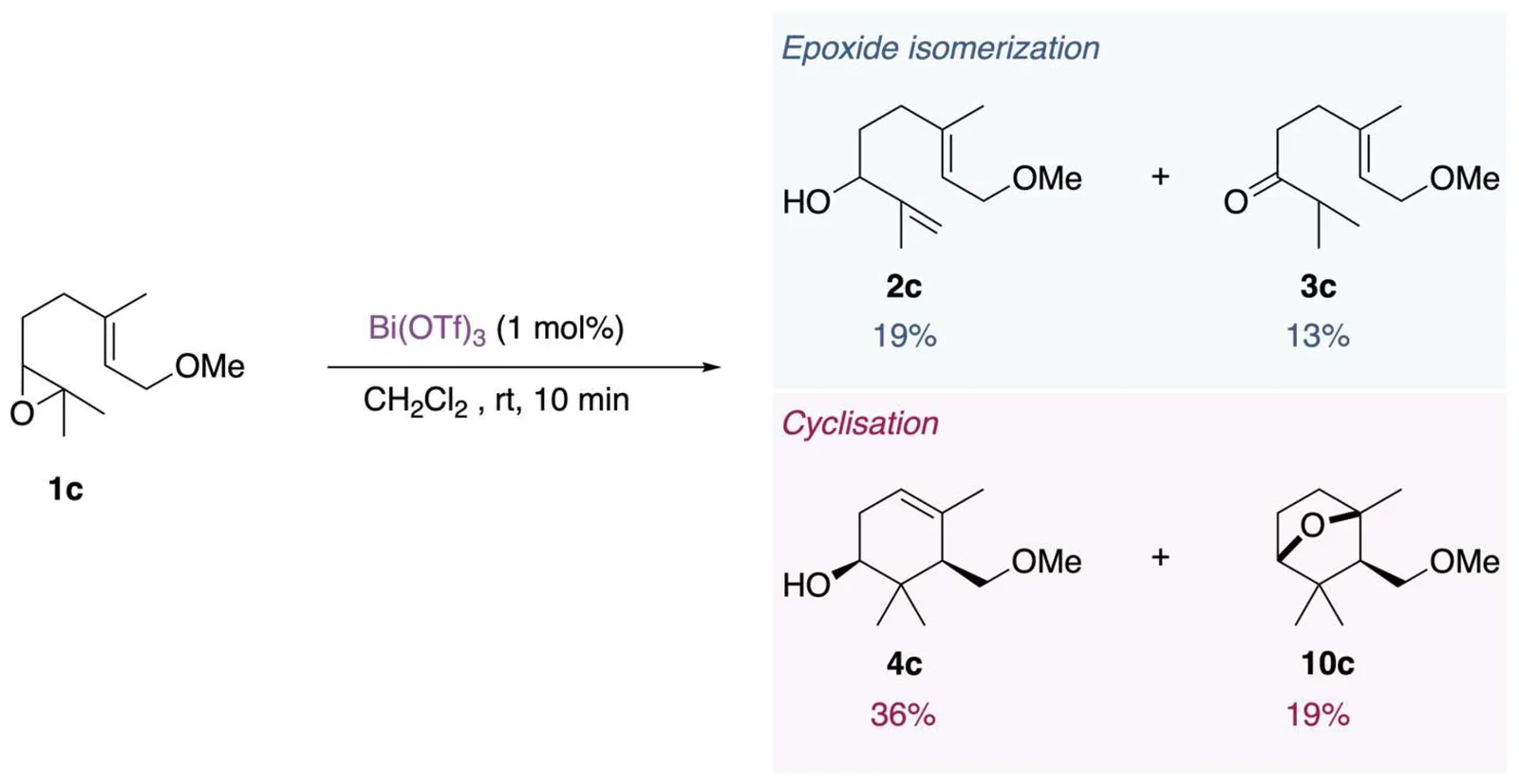
Reactivity of **1c** with 1 mol% of Bi(OTf)_3_.

To extend the scope of the reaction, monoepoxides derived from geraniol were examined under similar catalytic conditions. The 6,7-epoxygeraniol methyl ether (**1c**) was selected as the model substrate. The reactivity of **1c** in the presence of 1 mol% of Bi(OTf)_3_ under the same conditions as in Scheme 2 led to the isolation of four compounds: the epoxide isomerization products **2c** and **3c**, the cyclic compound **4c** resulting from intramolecular olefin addition, as well as a bicyclic compound **10c**, as illustrated in Scheme 7.

Bismuth (III) triflate efficiently catalyzed the cyclization through olefin addition to the activated epoxide. Bicyclic compound **10c** may be derived from a second cyclization from the unsaturated alcohol **4c**, the global yield of cyclization being 55%. As the intramolecular cyclization of olefinic alcohols catalyzed by metal triflates has already been reported [28], this hypothesis was investigated by resubmitting **4c** to the cyclization conditions. However, no formation of the bicyclic product **10c** was observed. This suggests that the formation of **10c** occurs independently rather than through interconversion from **4c**.

The relative stereochemistries of **4c** and **10c** were established by ^1^H–^1^H NOESY NMR experiments. Their stereochemical outcome can be rationalized using the Stork–Eschenmoser principle [29,30]. However, it is noteworthy that **10c** was detected as a mixture of two diastereoisomers (dr 2:1), and NOESY analysis allowed the major isomer to be assigned as the *cis* isomer, as depicted in Scheme 7.

Given the relative efficiency and selectivity observed for the Bi(III)-catalyzed cyclization, other metal triflates were subsequently evaluated for the reactivity of **1c**. The influence of the catalyst nature and the reaction temperature is summarized in Table 2.

The combined cyclization yield to **4c** and **10c** at room temperature reaches 55–57% with the different metal triflates tested (entries 1–5), indicating that this family of catalysts is able to activate the epoxide in a highly catalytic manner. The limited variation in product distribution when changing the metal center suggests that these M^3+^ cations exhibit comparable Lewis acidities in this context. As a result, the reaction appears to be weakly dependent on the nature of the metal and rather governed by the overall Lewis acidity imparted by the triflate ligands, pointing toward a common activation mode (i.e., metal-epoxide coordination).

The cyclization carried out at 80 °C, in 1,2-dichloroethane, with 1 mol% of Bi(OTf)_3_ (entry 6) afforded **4c** (47%) and **10c** (19%) almost exclusively, with a global yield of 66%, despite some decomposition (polymerization). An increase in temperature favors the intramolecular cyclization pathways relative to competing intermolecular processes. Under these conditions, the rate of ring closure becomes higher than that of side-reactions, leading to preferential formation of the cyclized products **4c** and **10c**, while linear intermediates are more rapidly consumed.

When 0.5 mol% of Bi(OTf)_3_ was used (entry 7), the overall cyclization yield remained at 66%, without any isomerization products **2c** or **3c**. A possible explanation is that a milder activation at room temperature with a reduced catalyst loading may provide better control over the formation and lifetime of the cationic intermediates, thereby favoring the intramolecular cyclization pathway over competing isomerization processes.

Notably, interconversion between the linear compounds **2c** and **3c** does not lead to the cyclic products **4c** and **10c**, indicating that these parameters influence the reaction pathway at the transition-state level rather than through product equilibration. Increasing the temperature suggests that cyclization is associated with the thermally favored pathway. Additionally, decreasing the catalyst loading may result in a milder activation of the epoxide, sufficient to slow down competing isomerization processes and allow time for intramolecular olefin addition, ultimately leading to the cyclized products.

**Scheme 8:**
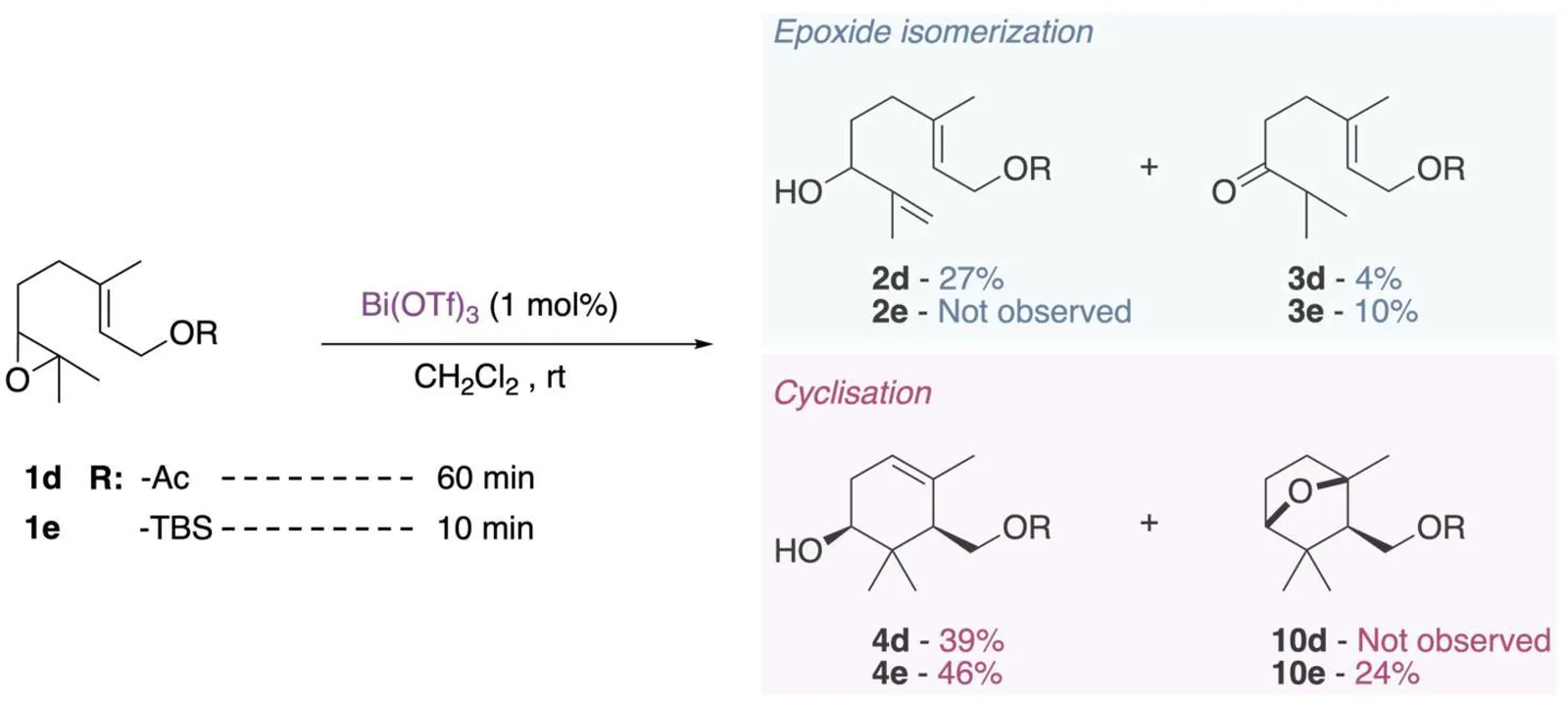
Reactivity of **1d**–**1e** with 1 mol% of Bi(OTf)_3_, in CH_2_Cl_2_ at room temperature.

The C(1) protecting group markedly influenced cyclization efficiency among the geraniol derivatives **1c**–**1e**. The substitution of the methoxy group in **1c** with an acetyl or a *tert*-butyldimethylsilyl substituent allowed us to evaluate the influence of the protecting group with **1d** and **1e**, respectively, as illustrated in Scheme 8.

Acetyl derivative **1d** afforded complete conversion after 1 h at room temperature with a global 60% yield of cyclization. After 10 min of reaction, the conversion of **1d** reached 47%, highlighting a lower reactivity than epoxyolefin **1c**. In addition, bicyclic product **10d** was not observed. This suggests that its formation may be less favorable under the reaction conditions. Possible explanations include a slower second cyclization step, incomplete conversion, or competing side reactions.

The silyl-substitution led to 70% of combined **4e** and **10e** cyclization after 10 min reaction at room temperature, affording the highest cyclization rate among the substrates tested. One possible explanation is that the electron-donating character of the silyl substituent partially offsets the electron-withdrawing effect of the ether oxygen, thereby slightly increasing the electron density of the alkene and favoring cyclization. However, prolonged exposure of the products to Bi(OTf)_3_ results in partial desilylation, generating free alcohols that can undergo subsequent rearrangements or side-reactions, thus increasing the complexity of the reaction mixture over time. Nevertheless, when carefully controlled and quenched at an early stage, this transformation provides access to the highest overall cyclization yield (70%). These results can be directly compared to those reported by Wang and coworkers, who, using the same substrate, reported 80% cyclization but under stoichiometric conditions with ZrCl_4_ [24].

**Scheme 9:**
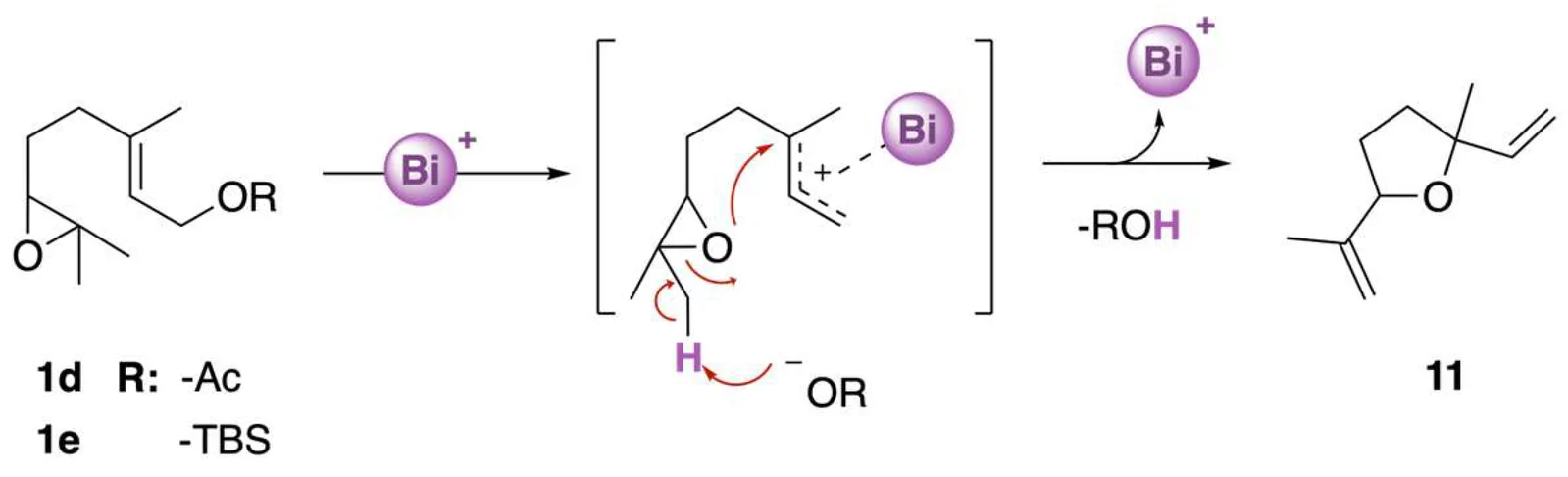
Proposed mechanism for the formation of **11** from **1d** and **1e**, in the presence of Bi(OTf)_3_.

At 80 °C, both substrates underwent a partial loss of the -OR group and led to the formation of the dihydrofuran derivative **11** (Scheme 9), which was identified in the crude mixture in 38% yield from **1d** and in 24% yield from **1e** (GC analysis).

**Scheme 10:**
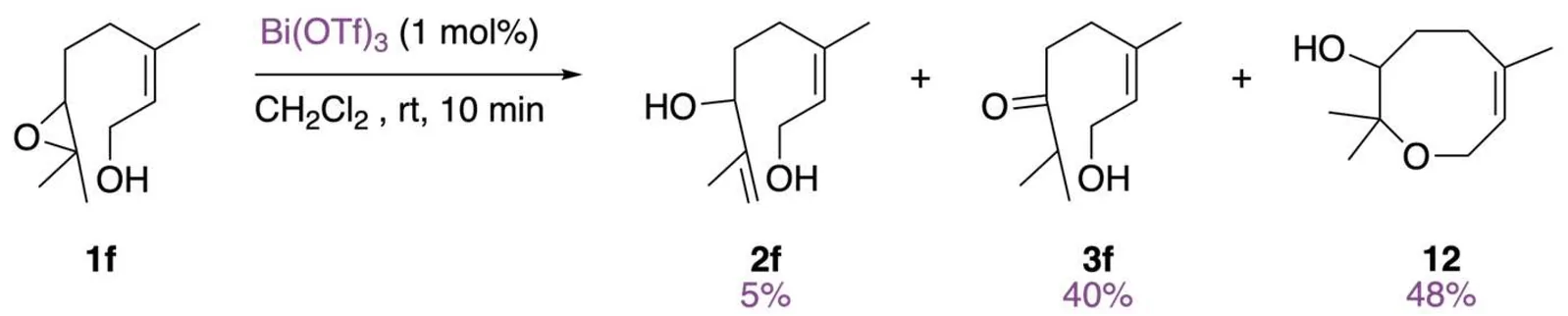
Cyclization of 6,7-epoxynerol using 1 mol% of Bi(OTf)_3_.

We further examined the reactivity of the unprotected alcohol. Cyclization of the unprotected 6,7-epoxygeraniol led to a mixture of compounds, whereas its corresponding (*Z*)-isomer, **1f** (nerol), afforded the eight-membered cyclic ether **12** as the major product in 48% yield (Scheme 10). Compound **12** results from the direct condensation of the hydroxyl group of **1f** on the epoxide, highlighting the necessity of a protecting group when performing these cyclizations under catalytic conditions. Nevertheless, such eight-membered ring cyclic ethers might be valuable intermediates for the synthesis of complex natural products.

## 3. Materials and Methods

### 3.1. General Information

All commercially available reagents were purchased from Sigma-Aldrich (St. Louis, MO, USA), BLDPharm (Shanghai, China), TCI (Tokyo, Japan), Alfa Aesar (Ward Hill, MA, USA), and Fluorochem (Glossop, UK), and were used without further purification unless otherwise stated. Solvents (≥99.5% purity or “solvent for synthesis”) were obtained from Sigma-Aldrich and Carlo Erba Reagents. Deuterated solvents were purchased from Sigma-Aldrich and, when necessary, passed through a short plug of basic alumina prior to NMR analysis.

Air-sensitive reactions were performed in vacuum-dried glassware under an inert nitrogen atmosphere using anhydrous solvents. Glassware was dried by heating under vacuum prior to use. Anhydrous dichloromethane was purchased from Sigma-Aldrich and titrated by Karl Fischer before each reaction. Metal triflates were stored in a desiccator under dry conditions and dried under vacuum heating prior to use.

Column chromatography was carried out on high-purity silica gel (pore size 60 Å, 40–63 µm, 230–400 mesh, Sigma-Aldrich).

NMR spectra (^1^H, ^13^C, HSQC, HMBC, COSY, NOESY) were recorded on Bruker Avance 400, 500 and 600 MHz spectrometers at 300 K. Chemical shifts (δ) are reported in ppm relative to TMS and were calibrated to residual solvent signals (CDCl_3_: ^1^H δ 7.26, ^13^C δ 77.16). Coupling constants (*J*) are reported in Hz. Signal multiplicities are denoted as s (singlet), d (doublet), t (triplet), q (quartet), quint. (quintet), sext. (sextet), hept. (heptet), m (multiplet), and br (broad). All NMR spectra were processed using MestReNova 16.0.0.

GCMS analyses were carried out on an Agilent 7820A GC system coupled to an Agilent 5977B MSD (Agilent, Santa Clara, CA, USA) using a Teknokroma SAPIENS-5MS capillary column (5% diphenyl—95% dimethylpolysiloxane, 10 m × 100 µm × 0.10 µm). Helium was used as carrier gas. The injection temperature was 250 °C; transfer line temperature, 300 °C; ion source temperature, 230 °C. The injection volume was 0.2 µL, with a split ratio of 1:50. The ionization energy was 70 eV; and the scan range was *m*/*z* 35–550.

### 3.2. Experimental and Characterization Data

#### 3.2.1. Representative Isomerization and Cyclization of Epoxyolefins

Round-bottom flasks were dried under vacuum with heating, allowed to cool, and charged with the selected Lewis or Brønsted acid catalyst (1 mol%). The flask was further dried under vacuum with heating, then purged three times with nitrogen. A solution of the substrate in anhydrous CH_2_Cl_2_ was added dropwise under a nitrogen atmosphere. The reaction mixture was stirred at ambient temperature until complete conversion, as determined by GCMS analysis. The reaction was quenched with saturated aqueous Na_2_CO_3_, and the organic layer was separated, washed with water and brine, dried over anhydrous MgSO_4_, and concentrated under reduced pressure. The crude product was purified by flash column chromatography on silica gel.

For reaction monitoring, aliquots (0.10 mL) were periodically withdrawn and quenched into saturated aqueous NaHCO_3_ (1 mL). The aqueous phase was extracted with Et_2_O (1 mL), and the organic layer was dried over anhydrous MgSO_4_ to afford samples for GCMS injection.

#### 3.2.2. Characterization of the Isolated Compounds

2,2-dimethyl-3-(((3-methylbut-2-en-1-yl)oxy)methyl)oxirane (**1a**): **GCMS** [M-CH_3_]^+^ *m*/*z* = 155. **^1^H NMR** (400 MHz, CDCl_3_) δ 5.30 (t, *J* = 6.9 Hz, 1H), 4.10–3.95 (m, 2H), 3.59 (dd, *J* = 10.8, 4.9 Hz, 1H), 3.51 (dd, *J* = 10.8, 4.9 Hz, 1H), 2.96 (t, *J* = 5.4 Hz, 1H), 1.75 (s, 1H), 1.69 (s, 1H), 1.34 (s, 1H), 1.29 (s, 1H). **^13^C NMR** (101 MHz, CDCl_3_) δ 137.70, 120.90, 68.75, 67.76, 62.29, 57.90, 25.93, 24.91, 19.04, 18.16.
2,2-dimethyl-3-(5-methylhex-4-en-1-yl)oxirane (**1b**) [31]: **GCMS** [M]^+^ *m*/*z* = 152. **^1^H NMR** (400 MHz, CDCl_3_) δ 5.16–5.05 (m, 1H), 1.97 (q, *J* = 7.2 Hz, 2H), 1.69 (q, J = 1.3 Hz, 3H), 1.60 (d, *J* = 1.5 Hz, 3H), 1.51–1.29 (m, 2H).
(*E*)-3-(5-methoxy-3-methylpent-3-en-1-yl)-2,2-dimethyloxirane (**1c**) [32]: **GCMS** [M-CH_3_]^+^ *m*/*z* = 169. ^**1**^**H NMR** (400 MHz, CDCl_3_) δ 5.38 (t, *J* = 6.7 Hz, 1H), 3.93 (d, *J* = 6.8 Hz, 2H), 3.32 (s, 3H), 2.71 (t, *J* = 6.2 Hz, 1H), 2.28–2.08 (m, 2H), 1.69 (s, 3H), 1.68–1.62 (m, 2H), 1.30 (s, 3H), 1.25 (s, 3H).
(*E*)-5-(3,3-dimethyloxiran-2-yl)-3-methylpent-2-en-1-yl acetate (**1d**) [33]: GCMS [M-CH_3_]^+^ *m*/*z* = 197. **^1^H NMR** (400 MHz, CDCl_3_) δ 5.38 (t, *J* = 7.0 Hz, 1H), 4.59 (d, *J* = 7.1 Hz, 2H), 2.69 (t, *J* = 6.2 Hz, 1H), 2.29–2.09 (m, 2H), 2.04 (s, 3H), 1.72 (s, 3H), 1.65–1.60 (m, 2H), 1.30 (s, 3H), 1.25 (s, 3H).**^13^C NMR** (101 MHz, CDCl_3_) δ 171.18, 141.39, 119.06, 64.04, 61.38, 58.49, 36.32, 27.21, 24.96, 21.16, 18.88, 16.59.
(*E*)-tert-butyl((5-(3,3-dimethyloxiran-2-yl)-3-methylpent-2-en-1-yl)oxy) dimethylsilane (**1e**) [34]: **GCMS** [M-*t*Bu]^+^ *m*/*z* = 227. **^1^H NMR** (400 MHz, CDCl_3_) δ 5.34 (t, *J* = 6.3 Hz, 1H), 4.19 (d, *J* = 6.3 Hz, 2H), 2.71 (t, *J* = 6.2 Hz, 1H), 2.23–2.04 (m, 2H), 1.73–1.56 (m, 2H), 1.64 (s, 3H), 1.30 (s, 3H), 1.26 (s, 3H), 0.90 (s, 9H), 0.07 (s, 6H).
(*Z*)-5-(3,3-dimethyloxiran-2-yl)-3-methylpent-2-en-1-ol (**1f**) [35]: **GCMS**: [M-CH_3_]^+^ *m*/*z* = 227. ^1^H NMR (400 MHz, CDCl_3_) δ 5.51 (td, *J* = 7.2–1.6 Hz, 1H), 4.20–4.05 (m, 2H), 2.73 (dd, *J* = 7.7–5.1 Hz, 1H), 2.36–2.16 (m, 2H), 1.77 (s, 3H), 1.73–1.64 (m, 2H), 1.30 (s, 3H), 1.27 (s, 3H).
3-methyl-1-((3-methylbut-2-en-1-yl)oxy)but-3-en-2-ol (**2a**): Cyclization performed on 500 mg of **1a**. Isolated yield: 16%. **GCMS** [M-CH_3_]^+^ *m*/*z* = 155. **^1^H NMR** (500 MHz, CDCl_3_) **δ** 5.35 (tdq, *J* = 7.0, 2.9, 1.4 Hz, 1H), 5.06 (s, 1H), 4.92 (s, 1H), 4.24 (d, *J* = 8.1 Hz, 1H), 4.02 (d, *J* = 7.0 Hz, 2H), 3.53 (dd, *J* = 9.6, 3.2 Hz, 1H), 3.34 (dd, *J* = 9.7, 8.4 Hz, 1H), 2.51 (s, 1H), 1.75 (s, 6H), 1.68 (s, 3H). **^13^C NMR** (125 MHz, CDCl_3_) **δ** 144.03, 137.57, 120.89, 112.01, 74.03, 73.21, 67.78, 25.93, 18.94, 18.18.
3-methyl-1-((3-methylbut-2-en-1-yl)oxy)butan-2-one (**3a**) [36]: Cyclization performed on 500 mg of **1a**. Isolated yield: 6%. **GCMS** [M-CH_3_]^+^ *m*/*z* = 155. **^1^H NMR** (400 MHz, CDCl_3_) **δ** 5.35 (tp, *J* = 7.1, 1.4 Hz, 1H), 4.10 (s, 2H), 4.03 (d, *J* = 7.0 Hz, 2H), 2.77 (hept, *J* = 7.0 Hz, 1H), 1.75 (s, 3H), 1.68 (s, 3H), 1.09 (d, *J* = 7.0 Hz, 6H). **^13^C NMR** (101 MHz, CDCl_3_) **δ** 212.57, 138.30, 120.45, 73.36, 67.78, 37.11, 25.94, 18.17, 18.15.
*cis*-4,4-dimethyl-5-(prop-1-en-2-yl)tetrahydro-2H-pyran-3-ol (**4a**): Cyclization performed on 500 mg of **1a**. Isolated yield: 1%. **GCMS** [M]^+^ *m*/*z* = 170; [M-CH_3_]^+^ *m*/*z* = 155. **^1^H NMR** (500 MHz, CDCl_3_) **δ** 5.01–4.99 (m, 1H), 4.64–4.59 (m, 1H), 3.77–3.59 (m, 2H), 3.52 (d, *J* = 11.1 Hz, 1H), 3.48–3.44 (m, 1H), 3.23 (d, *J* = 11.3 Hz, 1H), 2.63–2.56 (m, 1H), 1.78 (s, 3H), 1.09 (s, 3H), 0.92 (s, 3H). **^13^C NMR** (125 MHz, CDCl_3_) **δ** 144.08, 112.48, 72.18, 71.50, 64.94, 43.40, 34.77, 24.07, 22.77, 22.68.
*trans*-3,3-dimethyl-5-(prop-1-en-2-yl)tetrahydro-2H-pyran-4-ol (**5a**) [37]: Cyclization performed on 500 mg of **1a**. Isolated yield: 2%. **GCMS** [M]^+^ *m*/*z* = 170; [M-CH_3_]^+^ *m*/*z* = 155. **^1^H NMR** (400 MHz, CDCl_3_) **δ** 4.95–4.89 (m, 1H), 4.87–4.82 (m, 1H), 3.80 (ddd, *J* = 11.3, 4.8, 1.4 Hz, 1H), 3.48–3.36 (m, 1H), 3.31 (d, *J* = 10.6 Hz, 1H), 3.16 (t, *J* = 11.4 Hz, 1H), 3.05 (dd, *J* = 11.5, 0.9 Hz, 1H), 2.36 (td, *J* = 11.0, 4.7 Hz, 1H), 1.67 (s, 3H), 0.97 (s, 3H), 0.87 (s, 3H). **^13^C NMR** (101 MHz, CDCl_3_) **δ** 142.51, 114.57, 78.08, 76.22, 71.30, 48.50, 36.02, 23.71, 20.68, 17.64.
2,2-dimethyl-3-((3-methylbut-2-en-1-yl)oxy)propanal (**6a**) [37]: Cyclization performed on 500 mg of **1a**. Isolated yield: 6%. **GCMS** [M]^+^ *m*/*z* = 170. **^1^H NMR** (400 MHz, CDCl_3_) **δ** 9.55 (s, 1H), 5.27 (tdt, *J* = 6.8, 2.8, 1.4 Hz, 1H), 3.94 (d, *J* = 6.8 Hz, 2H), 3.39 (s, 2H), 1.73 (s, 3H), 1.64 (s, 3H), 1.07 (s, 6H). **^13^C NMR** (101 MHz, CDCl_3_) δ 205.70, 137.03, 121.11, 75.09, 68.12, 47.21, 25.88, 19.22, 18.18.
2,2,5,5-tetramethyl-3,6-bis(((3-methylbut-2-en-1-yl)oxy)methyl)-1,4-dioxane (**7**): Cyclization performed on 500 mg of **1a**. Isolated yield: 3%. 2 diastereomers (dr 1:1). **GCMS** [M]^+^ *m*/*z*= 340. **^1^H NMR** (500 MHz, CDCl_3_) δ 5.32 (t, *J* = 7.0 Hz, 2H), 4.08–3.99 (m, 2H), 3.98–3.90 (m, 2H), 3.76–3.67 (m, 2H), 3.34 (d, *J* = 5.6 Hz, 4H), 1.73 (s, 6H), 1.66 (s, 6H), 1.22–1.16 (m, 12H). **^13^C NMR** (126 MHz, CDCl_3_) δ 136.78, 121.12, 75.44, 74.64, 73.04, 69.98, 67.85, 25.78, 23.44, 22.11, 18.06.
3-methyl-3-((3-methyl-1-((3-methylbut-2-en-1-yl)oxy)but-3-en-2-yl)oxy)-1-((3-methylbut-2-en-1-yl)oxy)butan-2-ol (**8**): Cyclization performed on 500 mg of **1a**. Isolated yield: 8%. 2 diastereomers (dr 1:1). **GCMS** M[C_13_H_23_O_2_]^+^ *m*/*z* = 211. **^1^H NMR** (400 MHz, CDCl_3_) δ 5.33–5.28 (m, 1H), 5.27–5.21 (m, 1H), 4.95 (s, 1H), 4.81 (s, 1H), 4.31 (s, 1H), 4.19 (dd, *J* = 7.5–4.5 Hz, 1H), 4.09 (t, *J* = 6.1 Hz, 1H, diastereoisomer), 4.03–3.87 (m, 4H), 3.82–3.75 (m, 1H), 3.64 (dd, *J* = 7.8–3.1 Hz, 1H, diastereoisomer), 3.57 (s, 1H, diastereoisomer), 3.49–3.23 (m, 4H), 1.70–1.54 (m, 15H), 1.10 (s, 3H), 1.07 (s, 3H); **^13^C NMR** (126 MHz, CDCl_3_) δ diastereoisomer 1: 145.80, 137.04, 136.70, 121.29, 120.96, 112.51, 77.81, 75.46, 73.34, 73.03, 71.49, 67.81, 67.74, 25.78, 25.75, 24.53, 22.57, 18.86, 18.07, 18.04; diastereoisomer 2: 145.17, 136.76, 136.62, 121.24, 120.56, 112.40, 77.75, 77.41, 74.33, 72.94, 70.95, 67.76, 67.69, 25.78, 25.75, 22.50, 19.68, 18.60, 18.07, 18.04.
4,4-dimethyl-2-(2-methyl-1-((3-methylbut-2-en-1-yl)oxy)propan-2-yl)-5-(((3-methylbut-2-en-1-yl)oxy)methyl)-1,3-dioxolane (**9**): Cyclization performed on 500 mg of **1a**. Isolated yield: 10%. 2 diastereomers (dr 1:1). **GCMS** [M-prenyl]^+^ *m*/*z* = 271. **^1^H NMR** (400 MHz, CDCl_3_) δ 5.34 (m, 2H), 4.78 (s, 1H), 4.08–3.96 (m, 2H), 3.93 (d, *J* = 6.8 Hz, 2H), 3.77 (dd, *J* = 6.6–5.5 Hz, 1H), 3.52 (dd, *J* = 10.1–6.6 Hz, 1H), 3.41 (dd, *J* = 10.1–5.5 Hz, 1H), 3.23 (s, 2H), 1.74 (dd, *J* = 6.3–1.4 Hz, 6H), 1.66 (dd, *J* = 11.4–1.4 Hz, 6H), 1.29 (s, 3H), 1.12 (s, 3H), 0.93 (d, *J* = 3.5 Hz, 6H); **^13^C NMR** (101 MHz, CDCl_3_) δ 137.33, 136.16, 121.91, 121.09, 105.42, 82.99, 78.94, 76.29, 68.91, 68.27, 67.93, 38.16, 26.37, 25.93, 22.73, 19.71, 19.63, 18.22, 18.18.
2,8-dimethylnona-1,7-dien-3-ol (**2b**) [38]: Cyclization performed on 42.1 mg of **1b**. Isolated yield: 12%. **GCMS** [M]^+^ *m*/*z* = 168; [M-H_2_O]^+^ *m*/*z* = 150; [M-H_2_O-CH_3_]^+^ *m*/*z* = 135. **^1^H NMR** (500 MHz, CDCl_3_) **δ** 5.11 (t, *J* = 7.4 Hz, 1H), 4.93 (s, 1H), 4.83 (t, *J* = 1.8 Hz, 1H), 4.06 (t, *J* = 6.6 Hz, 1H), 2.00 (q, *J* = 7.5 Hz, 2H), 1.72 (s, 3H), 1.68 (s, 3H), 1.60 (s, 3H), 1.58–1.49 (m, 4H). ^1^**^3^C NMR** (125 MHz, CDCl_3_) **δ** 147.78, 131.82, 124.55, 111.14, 76.12, 34.66, 27.99, 25.95, 25.87, 17.85, 17.61.
2,8-dimethylnon-7-en-3-one (**3b**) [39]: Cyclization performed on 42.1 mg of **1b**. Isolated yield: 8%. **GCMS** [M]^+^ *m*/*z* = 168. **^1^H NMR** (600 MHz, CDCl_3_) **δ** 5.08 (dd, *J* = 7.2, 1.4 Hz, 1H), 2.59 (hept, *J* = 6.9 Hz, 1H), 2.43 (t, *J* = 7.4 Hz, 2H), 2.04–1.94 (m, 2H), 1.68 (t, *J* = 1.3 Hz, 4H), 1.64–1.58 (m, 5H), 1.08 (d, *J* = 6.9 Hz, 6H). **^13^C NMR** (150 MHz, CDCl_3_) **δ** 215.18, 132.41, 124.02, 40.94, 39.89, 27.60, 25.86, 24.03, 18.43, 17.85.
*cis*-2,2-dimethyl-3-(prop-1-en-2-yl)cyclohexan-1-ol (**4b**): Cyclization performed on 42.1 mg of **1b**. Isolated yield: 6%. **GCMS** [M]^+^ *m*/*z* = 168. **^1^H NMR** (600 MHz, CDCl_3_) δ 4.86 (p, *J* = 1.6 Hz, 1H), 4.65 (dt, *J* = 2.3, 0.8 Hz, 1H), 3.26 (dd, *J* = 11.5, 4.2 Hz, 1H), 1.78 (dd, *J* = 12.9, 3.2 Hz, 1H), 1.75 (dd, *J* = 1.5, 0.8 Hz, 3H), 1.74–1.69 (m, 1H), 1.56 (qd, *J* = 13.1, 3.8 Hz, 1H), 1.49–1.31 (m, 4H), 0.98 (s, 3H), 0.85 (s, 3H). **^13^C NMR** (125 MHz, CDCl_3_) **δ** 146.88, 113.15, 78.83, 53.60, 39.52, 30.79, 27.57, 26.46, 24.49, 24.24, 13.73.
(*E*)-8-methoxy-2,6-dimethylocta-1,6-dien-3-ol (**2c**): Cyclization performed on 500 mg of **1c**. Isolated yield: 16%. **GCMS** [M]^+^ *m*/*z* = 184; [M-CH_3_]^+^ *m*/*z* = 169. **^1^H NMR** (400 MHz, CDCl_3_) **δ** 5.37 (ddt, *J* = 6.8, 5.5, 1.3 Hz, 1H), 4.97–4.91 (m, 1H), 4.87–4.81 (m, 1H), 4.07–4.03 (m, 1H), 3.93 (d, *J* = 6.8 Hz, 2H), 3.32 (s, 3H), 2.18–1.97 (m, 2H), 1.72 (d, *J* = 1.1 Hz, 3H), 1.68 (d, *J* = 1.3 Hz, 3H), 1.67–1.60 (m, 2H). **^13^C NMR** (101 MHz, CDCl_3_) **δ** 147.55, 140.19, 121.13, 111.23, 75.65, 69.05, 57.95, 35.63, 33.06, 17.69, 16.59.
(*E*)-8-methoxy-2,6-dimethyloct-6-en-3-one (**3c**) [40]: Cyclization performed on 500 mg of **1c**. Isolated yield: 12%. **GCMS** [M]^+^ *m*/*z* = 184; [M-CH_3_]^+^ *m*/*z* = 169; [M-MeOH]^+^ *m*/*z* = 152. **^1^H NMR** (400 MHz, CDCl_3_) **δ** 5.33 (tt, *J* = 6.7, 1.3 Hz, 1H), 3.91 (d, *J* = 6.6 Hz, 2H), 3.32 (s, 3H), 2.66–2.53 (m, 3H), 2.28 (t, *J* = 7.8 Hz, 2H), 1.68 (d, *J* = 1.3 Hz, 3H), 1.09 (d, *J* = 6.9 Hz, 6H). **^13^C NMR** (101 MHz, CDCl_3_) **δ** 214.14, 139.33, 121.15, 69.00, 58.02, 41.03, 38.67, 33.25, 18.37, 16.76.
*cis*-5-(methoxymethyl)-4,6,6-trimethylcyclohex-3-en-1-ol (**4c**) [24]: Cyclization performed on 500 mg of **1c**. Isolated yield: 6%. **GCMS** [M]^+^ *m*/*z* = 184; [M-H_2_O]^+^ *m*/*z* = 166; [M-MeOH]^+^ *m*/*z* = 152; [M-H_2_O-MeOH]^+^ *m*/*z* = 134. **^1^H NMR** (400 MHz, CDCl_3_) **δ** 5.34 (ddq, *J* = 4.5, 3.0, 1.5 Hz, 1H), 3.76 (dd, *J* = 7.9, 5.7 Hz, 1H), 3.51 (dd, *J* = 10.0, 4.3 Hz, 1H), 3.41 (dd, *J* = 10.0, 3.4 Hz, 1H), 3.28 (s, 3H), 2.40 – 2.21 (m, 1H), 2.10 – 1.86 (m, 1H), 1.85 (d, *J* = 4.8 Hz, 1H), 1.73 (s, 3H), 1.00 (s, 3H), 0.91 (s, 3H). **^13^C NMR** (101 MHz, CDCl3) **δ** 133.81, 120.38, 72.27, 71.80, 58.75, 51.07, 36.95, 32.04, 23.18, 22.48, 21.61.
*cis*-2-(methoxymethyl)-1,3,3-trimethyl-7-oxabicyclo[2.2.1]heptane (**10c**): Cyclization performed on 500 mg of **1c**. Isolated yield: 10%. **GCMS** [M]^+^ *m*/*z* = 184; [M-CH_3_]^+^ *m*/*z* = 169; [M-MeOH]^+^ *m*/*z* = 152. **^1^H NMR** (400 MHz, CDCl_3_) **δ** major diastereomer: 3.73 (d, *J* = 5.3 Hz, 1H), 3.36–3.32 (m, 2H), 3.29 (s, 3H), 1.92 (ddd, *J* = 12.4, 9.1, 4.5 Hz, 1H), 1.74–1.37 (m, 4H), 1.33 (s, 3H), 1.06 (s, 3H), 1.00 (s, 3H); minor diastereomer: 3.80 (d, *J* = 5.2 Hz, 1H), 3.36–3.31 (m, 2H), 3.30 (s, 3H), 1.95–1.92 (m, 1H), 1.74–1.48 (m, 5H), 1.44 (s, 3H), 1.09 (s, 3H), 0.89 (s, 3H). **^13^C NMR** (101 MHz, CDCl_3_) **δ** major diastereomer*:* 86.16, 86.10, 71.69, 58.60, 55.48, 44.89, 38.64, 26.07, 26.01, 22.97, 18.31; minor diastereomer*:* 87.12, 86.02, 71.80, 58.96, 55.96, 42.19, 32.13, 29.53, 26.56, 21.90, 19.09.
(*E*)-6-hydroxy-3,7-dimethylocta-2,7-dien-1-yl acetate (**2d**) [41]: Cyclization performed on 500 mg of **1d**. Isolated yield: 5%. **GCMS** [M-AcOH]^+^ *m*/*z* = 152. **^1^H NMR** (400 MHz, CDCl_3_) **δ** 5.41–5.29 (m, 1H), 4.93 (p, *J* = 0.8 Hz, 1H), 4.83 (p, *J* = 1.6 Hz, 1H), 4.57 (d, *J* = 7.1 Hz, 2H), 4.03 (t, *J* = 6.4 Hz, 1H), 2.20–2.05 (m, 1H), 2.03 (s, 3H), 1.72–1.69 (m, 8H). **^13^C NMR** (101 MHz, CDCl_3_) **δ** 171.24, 147.47, 142.05, 118.68, 111.28, 75.56, 61.45, 35.58, 32.92, 21.15, 17.68, 16.61.
(*E*)-3,7-dimethyl-6-oxooct-2-en-1-yl acetate (**3d**) [42]: Cyclization performed on 500 mg of **1d**. Isolated yield: 1%. **GCMS** [M-Ac]^+^ *m*/*z* = 170. **^1^H NMR** (400 MHz, CDCl_3_) **δ** 5.33 (tq, *J* = 7.0, 1.4 Hz, 1H), 4.57 (d, *J* = 7.0 Hz, 2H), 2.59 (hept, *J* = 6.8 Hz, 1H), 2.56 (t, *J* = 8.4 Hz, 2H), 2.30 (t, *J* = 7.7 Hz, 2H), 2.04 (s, 3H), 1.71 (s, 3H), 1.09 (d, *J* = 7.0 Hz, 6H). **^13^C NMR** (101 MHz, CDCl_3_) δ 213.81, 171.05, 141.08, 118.68, 61.21, 40.95, 38.38, 33.12, 21.02, 18.23, 16.62.
(*cis*-5-hydroxy-2,6,6-trimethylcyclohex-2-en-1-yl)methyl acetate (**4d**) [43]: Cyclization performed on 500 mg of **1d**. Isolated yield: 3%. **GCMS** [M-OAc]^+^ *m*/*z* = 152. **^1^H NMR** (400 MHz, CDCl_3_) **δ** 5.40–5.32 (m, 1H), 4.30 (dd, *J* = 11.8, 4.8 Hz, 1H), 4.10 (dd, *J* = 11.8, 3.7 Hz, 1H), 3.71 (t, *J* = 6.8 Hz, 1H), 2.35 (m, 1H), 2.03 (s, 3H), 2.01 (s, 1H), 1.97–1.85 (m, 1H), 1.72 (d, *J* = 1.9 Hz, 3H), 1.00 (s, 3H), 0.94 (s, 3H). **^13^C NMR** (101 MHz, CDCl_3_) **δ** 171.08, 132.78, 121.18, 72.13, 63.27, 49.62, 36.88, 32.07, 23.16, 22.47, 21.64, 21.25.
(*E*)-8-((*tert*-butyldimethylsilyl)oxy)-2,6-dimethyloct-6-en-3-one (**3e**): Cyclization performed on 500 mg of **1e**. Product obtained as a mix with **10e**, Fraction yield: 21%. **GCMS** [M-*t*Bu]^+^ *m*/*z* = 227. **^1^H NMR** (400 MHz, CDCl_3_) **δ** 5.29 (tq, *J* = 6.3, 1.3 Hz, 1H), 4.17 (dq, *J* = 6.2, 1.0 Hz, 2H), 2.59 (hept, *J* = 7.0 Hz, 1H), 2.56 (d, *J* = 8.1 Hz, 2H), 2.25 (t, *J* = 7.8 Hz, 2H), 1.62 (s, 3H), 1.08 (d, *J* = 7.0 Hz, 6H), 0.89 (s, 9H), 0.06 (s, 6H). **^13^C NMR** (101 MHz, CDCl_3_) **δ** 214.28, 135.94, 124.85, 60.36, 41.04, 38.74, 33.29, 32.26, 26.15, 16.69, −5.42.
*cis*-5-(((*tert*-butyldimethylsilyl)oxy)methyl)-4,6,6-trimethylcyclohex-3-en-1-ol (**4e**): Cyclization performed on 500 mg of **1e**. Isolated yield: 7%. **GCMS** [M-*t*Bu]^+^ *m*/*z* = 227. **^1^H NMR** (400 MHz, CDCl_3_) **δ** 5.35–5.31 (m, 1H), 3.91 (dd, *J* = 8.3, 5.8 Hz, 1H), 3.73 (dd, *J* = 3.7, 1.0 Hz, 2H), 2.38–2.26 (m, 1H), 1.93–1.81 (m, 1H), 1.73 (s, 1H), 1.71 (t, *J* = 1.9 Hz, 3H), 1.04 (s, 3H), 0.90 (s, 3H), 0.87 (s, 9H), 0.02 (d, *J* = 7.0 Hz, 6H). **^13^C NMR** (101 MHz, CDCl_3_) **δ** 133.48, 120.59, 72.31, 61.37, 53.48, 37.06, 32.27, 23.47, 22.66, 21.50, 18.27, −5.39, −5.45.
*tert*-butyldimethyl((*cis*-1,3,3-trimethyl-7-oxabicyclo[2.2.1]heptan-2-yl)methoxy)silane (**10e**) [44]: Cyclization performed on 500 mg of **1e**. Product obtained as a mix with **3e**, Fraction yield: 21%. **GCMS** [M-*t*Bu]^+^ *m*/*z* = 227. **^1^H NMR** (400 MHz, CDCl_3_) **δ** *major diastereomer*: 3.74–3.68 (d, *J* = 4.9 Hz, 1H), 3.65–3.56 (m, 1H), 3.56–3.45 (m, 1H), 1.97–1.86 (m, 1H), 1.74–1.65 (m, 1H), 1.60–1.49 (m, 1H), 1.47–1.42 (m, 1H), 1.33 (s, 3H), 1.29–1.20 (m, 1H), 1.07 (s, 3H), 1.04 (s, 3H), 0.88 (s, 9H), 0.03 (d, *J* = 1.0 Hz, 6H); *minor diastereomer*: 3.78 (d, *J* = 4.9 Hz, 1H), 3.65–3.56 (m, 1H), 3.56–3.45 (m, 1H), 1.97–1.86 (m, 1H), 1.74–1.65 (m, 1H), 1.60–1.49 (m, 1H), 1.47 – 1.42 (m, 1H), 1.33 (s, 3H), 1.29–1.20 (m, 1H), 1.07 (s, 3H), 1.04 (s, 3H), 0.88 (s, 9H), 0.04 (s, 6H). **^13^C NMR** (101 MHz, CDCl_3_) **δ** *major diastereomer*: 86.18, 86.01, 61.48, 57.76, 44.87, 36.85, 29.38, 26.18, 26.02, 22.90, 18.40, 18.10, −5.33; *minor diastereomer*: 87.28, 85.94, 61.58, 58.25, 42.09, 36.85, 29.38, 26.64, 26.04, 22.27, 18.49, 18.28, −4.93.
(*Z*)-3,7-dimethylocta-2,7-diene-1,6-diol (**2f**) [35]: Cyclization performed on 500 mg of **1f**. Product obtained as a mix with **S11**, Fraction yield: 11%**GCMS** [M]^+^ *m*/*z* = 170. **^1^H NMR** (400 MHz, CDCl_3_) **δ** 5.52 (t, *J* = 8.3 Hz, 1H), 4.89 (s, 1H), 4.78 (s, 1H), 4.19–4.08 (m, 2H), 3.97–3.89 (m, 1H), 2.49 (t, *J* = 1.2 Hz, 1H), 2.37–2.27 (m, 1H), 1.68 (d, *J* = 1.3 Hz, 3H), 1.65 (s, 3H), 1.62–1.54 (m, 2H). **^13^C NMR** (101 MHz, CDCl_3_) **δ** 147.73, 138.46, 125.65, 110.84, 74.35, 58.64, 33.01, 27.90, 23.00, 18.16.
(*Z*)-8-hydroxy-2,6-dimethyloct-6-en-3-one (**3f**): Cyclization performed on 500 mg of **1f**. Isolated yield: 23%. **GCMS** [M]^+^ *m*/*z* = 170. **^1^H NMR** (400 MHz, CDCl_3_) **δ** 5.53–5.44 (m, 1H), 4.16–4.09 (m, 2H), 2.59 (t, *J* = 7.1 Hz, 2H), 2.56 (hept, *J* = 7.0 Hz, 1H), 2.36 (t, *J* = 7.1 Hz, 2H), 2.10 (br s, 1H), 1.70 (s, 3H), 1.08 (d, *J* = 6.9 Hz, 6H). **^13^C NMR** (101 MHz, CDCl_3_) **δ** 214.73, 138.49, 125.47, 58.77, 41.17, 38.21, 25.73, 23.01, 18.31.
2-methyl-5-(prop-1-en-2-yl)-2-vinyltetrahydrofuran (**11**) [45]: Cyclization performed on 500 mg of **1d**. Isolated yield: 16%. **GCMS** [M-CH_3_]^+^ *m*/*z* = 137. **^1^H NMR** (400 MHz, CDCl_3_) **δ** 5.96–5.76 (m, 2H), 5.20–5.11 (m, 2H), 4.99–4.94 (m, 2H), 4.94–4.89 (m, 2H), 4.80–4.69 (m, 2H), 4.40–4.28 (m, 2H), 2.03–1.68 (m, 8H), 1.66 (d, *J* = 3.6 Hz, 6H), 1.29 (s, 3H), 1.27 (s, 3H). **^13^C NMR** (101 MHz, CDCl_3_) **δ** 146.24, 145.78, 144.65, 143.99, 111.61, 111.58, 110.53, 110.37, 83.38, 82.98, 82.30, 82.11, 38.17, 37.11, 31.13, 30.78, 26.97, 26.60, 18.45, 18.22.
(*Z*)-2,2,6-trimethyl-3,4,5,8-tetrahydro-2H-oxocin-3-ol (**12**) [35]: Cyclization performed on 500 mg of **1f**. Isolated yield: 20%. **GCMS** [M]^+^ *m*/*z* = 170. **^1^H NMR** (400 MHz, CDCl_3_) **δ** 5.16 (tt, *J* = 2.8, 1.4 Hz, 1H), 4.20 (d, *J* = 2.2 Hz, 2H), 3.88–3.78 (m, 1H), 3.17–3.03 (m, 1H), 1.96–1.75 (m, 3H), 1.74 (q, *J* = 0.8 Hz, 3H), 1.53 (d, *J* = 6.7 Hz, 1H), 1.28 (s, 3H), 1.23 (s, 3H). **^13^C NMR** (101 MHz, CDCl_3_) **δ** 134.71, 123.97, 79.10, 73.78, 63.29, 31.83, 28.15, 26.34, 23.80, 21.72.

## 4. Conclusions

Metal triflate-catalyzed reactions of epoxyolefins revealed a competition between epoxide isomerization and olefin cyclization, which is strongly governed by substrate structure and reaction conditions. After catalyst screening, Bi(OTf)_3_ at only 1 mol% emerged as a particularly efficient system, enabling fast transformations under mild conditions. This work establishes a catalytic (1 mol%) variant of transformations mainly reported under stoichiometric conditions, with GC yields comparable to those reported in the literature, highlighting the efficiency of metal triflates for epoxyolefin activation.

Our study shows that with substrate **1a**, bearing an oxygen atom in the main chain, the isomerization products were predominant, although the cyclization rate could be increased by increasing the temperature. The analogous substrate **1b**, without the chain oxygen, led to cyclization affording **4b** as the main compound. This difference is consistent with a coordination effect, where interaction of the Lewis acid with the chain oxygen likely stabilizes conformations that disfavor intramolecular olefin attack and instead promote competitive pathways.

The cyclizations with 6,7-epoxygeraniol derivatives indicated the formation of the expected cyclized compounds **4c**–**4e** and of the bicyclic compounds **10c**–**10e** in combined yields of 55 to 66% with only 1 mol% of bismuth triflate. These compounds were obtained stereoselectively affording the *cis*-alcohols **4c**–**4e**, following the Eschenmoser rules. These cyclic alcohols, with the same catalyst, could in turn be cyclized at the pendant olefin to afford stereoselectively the *cis*-bicyclic ether derivatives **10c**–**10e**. These results confirm the catalytic control of chemoselectivity (isomerization *vs* cyclization) by substrate structure. The unsuccessful case of substrate **1a** defines the scope and limitations of this method.

## Figures and Tables

**Table 1:** Influence of the nature of the catalyst on the reactivity of **1a** ^a^.

Entry	Catalyst (1 mol%)	Time	Conversion	% Obtained Compounds ^b^				
				2a	3a	4a	5a	6a
1	Bi(OTf)_3_	10 min	100%	49	22	6	10	-
2	Fe(OTf)_3_	10 min	100%	43	20	5	9	2
3	Al(OTf)_3_	10 min	100%	40	20	-	1	16
4	Sc(OTf)_3_	10 min	100%	39	20	1	1	14
5	In(OTf)_3_	10 min	100%	32	22	-	1	19
6	BiCl_3_	18 h	4%	1	1	-	-	2
7 ^c^	BF_3_.OEt_2_	2 h	39%	-	-	-	-	7
8	PTSA	24 h	25%	25	-	-	-	-

^a^ General reaction conditions: **1a** (0.5 to 1 mmol) in dry dichloromethane (0.033 M) with 1 mol% of metal triflate at room temperature. The reaction progress was monitored by GC analysis. Compounds were purified by column chromatography on silica gel using cyclohexane and ethyl acetate mixtures as eluents. ^b^ Ratios determined by GC analysis of the crude reaction closely agree with those obtained by ^1^H NMR integration. Compounds identified as dimerization products 0–32%. ^c^ Reaction no longer progressed after 2 h.

**Table 2:** Influence of the catalyst and the reaction conditions on the reactivity of **1c** ^a^.

Entry	Catalyst (mol%)	Time	Temperature	% Obtained Compounds ^b^				
				2c	3c	4c	10c	Global Cyclization ^c^
1	Bi(OTf)_3_ (1)	10 min	Rt	19	13	36	19	55
2	In(OTf)_3_ (1)	10 min	Rt	18	12	37	19	56
3	Fe(OTf)_3_ (1)	10 min	Rt	19	13	36	19	55
4	Al(OTf)_3_ (1)	60 min	Rt	18	12	35	22	57
5	Sc(OTf)_3_ (1)	10 min	Rt	18	12	38	19	57
6	Bi(OTf)_3_ (1)	10 min	80 °C	2	4	47	19	66
7	Bi(OTf)_3_ (0.5)	10 min	Rt	-	-	44	22	66

^a^ General reaction conditions: **1c** (0.5 to 1 mmol) in dry dichloromethane or 1,2-dichloroethane (0.033 M) with 1 mol% of metal triflate at room temperature. The reaction progress was monitored by GC analysis. Compounds were purified by column chromatography on silica gel using cyclohexane and ethyl acetate mixtures as eluents. ^b^ Ratios determined by GC analysis of the crude reaction mixture were consistent with those obtained by ^1^H NMR integration. Dimerized compounds: 13–34%. ^c^ Global cyclization refers to the sum of the cyclized compounds **4c** and **10c**.

## Data Availability

The original data presented in the study are included in the article/Supplementary Material; further inquiries can be directed to the corresponding author.

## Supplementary Materials

The following supporting information can be downloaded at: https://www.mdpi.com/article/10.3390/molecules31162828/s1, Figure S1: ^1^H NMR spectrum of **2a** (CDCl_3_, 500 MHz); Figure S2: ^13^C NMR spectrum of **2a** (CDCl_3_, 126 MHz); Figure S3: ^1^H NMR spectrum of **3a** (CDCl_3_, 400 MHz); Figure S4: ^13^C NMR spectrum of **3a** (CDCl_3_, 101 MHz); Figure S5: ^1^H NMR spectrum of **4a** (CDCl_3_, 500 MHz); Figure S6: ^13^C NMR spectrum of **4a** (CDCl_3_, 126 MHz); Figure S7: ^1^H—^13^C HSQC NMR spectrum of **4a** (CDCl_3_, 500/126 MHz); Figure S8: ^1^H—^1^H COSY NMR spectrum of **4a** (CDCl_3_, 500/500 MHz); Figure S9: ^1^H—^13^C HMBC NMR spectrum of **4a** (CDCl_3_, 500/126 MHz); Figure S10: ^1^H NMR spectrum of **5a** (CDCl_3_, 400 MHz); Figure S11: ^13^C NMR spectrum of **5a** (CDCl_3_, 101 MHz); Figure S12: ^1^H—^13^C HSQC NMR spectrum of **5a** (CDCl_3_, 400/101 MHz); Figure S13: ^1^H—^1^H COSY NMR spectrum of **5a** (CDCl_3_, 400/400 MHz); Figure S14: ^1^H—^13^C HMBC NMR spectrum of **5a** (CDCl_3_, 400/101 MHz); Figure S15: ^1^H—^1^H NOESY NMR spectrum of **5a** (CDCl_3_, 400/400 MHz); Figure S16: ^1^H NMR spectrum of **6a** (CDCl_3_, 400 MHz); Figure S17: ^13^C NMR spectrum of **6a** (CDCl_3_, 101 MHz); Figure S18: ^1^H NMR spectrum of **7** (CDCl_3_, 500 MHz); Figure S19: ^13^C NMR spectrum of **7** (CDCl_3_, 126 MHz); Figure S20: ^1^H NMR spectrum of **8** (CDCl_3_, 400 MHz); Figure S21: ^13^C NMR spectrum of **8** (CDCl_3_, 101 MHz); Figure S22: ^1^H NMR spectrum of **9** (CDCl_3_, 400 MHz); Figure S23: ^13^C NMR spectrum of **9** (CDCl_3_, 101 MHz); Figure S24: ^1^H NMR spectrum of **2b** (CDCl_3_, 400 MHz); Figure S25: ^13^C NMR spectrum of **2b** (CDCl_3_, 101 MHz); Figure S26: ^1^H NMR spectrum of **3b** (CDCl_3_, 400 MHz); Figure S27: ^13^C NMR spectrum of **3b** (CDCl_3_, 101 MHz); Figure S28: ^1^H NMR spectrum of **4b** (CDCl_3_, 400 MHz); Figure S29: ^13^C NMR spectrum of **4b** (CDCl_3_, 101 MHz); Figure S30: ^1^H—^13^C HSQC NMR spectrum of **4b** (CDCl_3_, 400/101 MHz); Figure S31: ^1^H—^1^H COSY NMR spectrum of **4b** (CDCl_3_, 400/400 MHz); Figure S32: ^1^H—^13^C HMBC NMR spectrum of **4b**; Figure S33: ^1^H—^1^H NOESY NMR spectrum of **4b** (CDCl_3_, 400/400 MHz); Figure S34: ^1^H NMR spectrum of **2c** (CDCl_3_, 400 MHz); Figure S35: ^13^C NMR spectrum of **2c**; Figure S36: ^1^H NMR spectrum of **3c** (CDCl_3_, 400 MHz); Figure S37: ^13^C NMR spectrum of **3c**; Figure S38: ^1^H NMR spectrum of **4c** (CDCl_3_, 400 MHz); Figure S39: ^13^C NMR spectrum of **4c** (CDCl_3_, 101 MHz); Figure S40: ^1^H—^13^C HSQC NMR spectrum of **4c** (CDCl_3_, 400/101 MHz); Figure S41: ^1^H—^1^H COSY NMR spectrum of **4c** (CDCl_3_, 400/400 MHz); Figure S42: ^1^H—^1^H NOESY NMR spectrum of **4c** (CDCl_3_, 400/400 MHz); Figure S43: ^1^H NMR spectrum of **10c** (CDCl_3_, 400 MHz); Figure S44: ^13^C NMR spectrum of **10c** (CDCl_3_, 101 MHz); Figure S45: ^1^H—^13^C HSQC NMR spectrum of **10c** (CDCl_3_, 400/101 MHz); Figure S46: ^1^H—^1^H COSY NMR spectrum of **10c** (CDCl_3_, 400/400 MHz); Figure S47: ^1^H—^1^H NOESY NMR spectrum of **10c** (CDCl_3_, 400/400 MHz); Figure S48: ^1^H NMR spectrum of **2d** (CDCl_3_, 400 MHz); Figure S49: ^13^C NMR spectrum of **2d** (CDCl_3_, 101 MHz); Figure S50: ^1^H NMR spectrum of **3d** (CDCl_3_, 400 MHz); Figure S51: ^13^C NMR spectrum of **3d** (CDCl_3_, 101 MHz); Figure S52: ^1^H NMR spectrum of **4d** (CDCl_3_, 400 MHz); Figure S53: ^13^C NMR spectrum of **4d** (CDCl_3_, 101 MHz); Figure S54: ^1^H—^13^C HSQC NMR spectrum of **4d** (CDCl_3_, 400/101 MHz); Figure S55: ^1^H—^1^H COSY NMR spectrum of **4d** (CDCl_3_, 400/400 MHz); Figure S56: ^1^H—^1^H NOESY NMR spectrum of **4d** (CDCl_3_, 400/400 MHz); Figure S57: ^1^H NMR spectrum of **11** (CDCl_3_, 400 MHz); Figure S58: ^13^C NMR spectrum of **11** (CDCl_3_, 101 MHz); Figure S59: ^1^H NMR spectrum of the not separable mixture of **3e** and **10e** (CDCl_3_, 400 MHz); Figure S60: ^13^C NMR spectrum of the not separable mixture of **3e** and **10e** (CDCl_3_, 101 MHz); Figure S61: ^1^H NMR spectrum of **4e** (CDCl_3_, 400 MHz); Figure S62: ^13^C NMR spectrum of **4e**; Figure S63: ^1^H—^13^C HSQC NMR spectrum of **4e** (CDCl_3_, 400/101 MHz); Figure S64: ^1^H—^1^H COSY NMR spectrum of **4e** (CDCl_3_, 400/400 MHz); Figure S65: ^1^H NMR spectrum of the not separable mixture of **2f** and dimer **S11** (CDCl_3_, 400 MHz); Figure S66: ^13^C NMR spectrum of the not separable mixture of **2f** and dimer **S11** (CDCl_3_, 101 MHz); Figure S67: ^1^H NMR spectrum of **3f** (CDCl_3_, 400 MHz); Figure S68: ^13^C NMR spectrum of **3f** (CDCl_3_, 101 MHz); Figure S69: ^1^H NMR spectrum of **12** (CDCl_3_, 400 MHz); Figure S70: ^13^C NMR spectrum of **12** (CDCl_3_, 101 MHz). See references cited in the Supplementary Materials [35,46,47,48,49,50,51,52,53].
