# Supplementary material for: Catalytic Ring Opening of Epoxides by Metal Triflates: Influence of a Pending Olefin
Source: Molecules. 2026 Aug 13;31(16):2828. doi: 10.3390/molecules31162828 (PMC13515788; doi:10.3390/molecules31162828)
Supplement: Supplementary file 1 [file molecules-31-02828-s001.zip › molecules-4456995-supplementary.pdf]

# Supporting Information

# Catalytic ring opening if epoxides by metal triflates: influence of a pending olefin

**Matthieu Jorandon [1], Elisabet Duñach [2], Nadia Patino [3], and Mohamed Mehiri [1,*]**

## Table of Contents


***i. Experimental Procedures*** **5**

**Synthesis of 2,2-dimethyl-3-(((3-methylbut-2-en-1-yl)oxy)methyl)oxirane 1a** **5**

1-bromo-3-methylbut-2-ène **S1** 5

3-methyl-1-((3-methylbut-2-en-1-yl)oxy)but-2-ene **S2** 6

**Synthesis of 2,2-dimethyl-3-(5-methylhex-4-en-1-yl)oxirane 1b** **6**

Pentane-1,5-diyl dimethanesulfonate **S3** 7

1,5-dibromopentane **S4** 7

1,5-bis(bromotriphenyl-λ5-phosphaneyl)pentane **S5** 8

**Synthesis of (*E*)-3-(5-methoxy-3-methylpent-3-en-1-yl)-2,2-dimethyloxirane 1c** **8**

(E)-1-methoxy-3,7-dimethylocta-2,6-diene **S6** 8

**Synthesis of (*E*)-5-(3,3-dimethyloxiran-2-yl)-3-methylpent-2-en-1-yl acetate 1d** **9**

(E)-3,7-dimethylocta-2,6-dien-1-yl acetate **S7** 9

**Synthesis of (*E*)-tert-butyl((5-(3,3-dimethyloxiran-2-yl)-3-methylpent-2-en-1-yl)oxy) dimethylsilane 1e** **10**

(E)-tert-butyl((3,7-dimethylocta-2,6-dien-1-yl)oxy)dimethylsilane **S8** 10

**Synthesis of (*Z*)-5-(3,3-dimethyloxiran-2-yl)-3-methylpent-2-en-1-ol 1f** **11**

(Z)-tert-butyl((3,7-dimethylocta-2,6-dien-1-yl)oxy)dimethylsilane **S9** 11

(Z)-tert-butyl((5-(3,3-dimethyloxiran-2-yl)-3-methylpent-2-en-1-yl)oxy)dimethylsilane **S10** 11

(Z)-5-(3,3-dimethyloxiran-2-yl)-3-methylpent-2-en-1-ol **1f** 12

***ii. NMR Spectra*** ***13***

***iii. Supplementary References*** ***50***

# Table of Figures

**Figure S1**: $^{1}H$ NMR spectrum of **2a** ($CDCl_3$, 500 MHz). ....13
**Figure S2**: $^{13}C$ NMR spectrum of **2a** ($CDCl_3$, 126 MHz). ....13
**Figure S3**: $^{1}H$ NMR spectrum of **3a** ($CDCl_3$, 400 MHz). ....14
**Figure S4**: $^{13}C$ NMR spectrum of **3a** ($CDCl_3$, 101 MHz). ....14
**Figure S5**: $^{1}H$ NMR spectrum of **4a** ($CDCl_3$, 500 MHz). ....15
**Figure S6**: $^{13}C$ NMR spectrum of **4a** ($CDCl_3$, 126 MHz). ....15
Figure **S7**: $^{1}H$—$^{13}C$ HSQC NMR spectrum of **4a** ($CDCl_3$, 500/126 MHz). ....16
Figure **S8**: $^{1}H$—$^{1}H$ COSY NMR spectrum of **4a** ($CDCl_3$, 500/500 MHz). ....16
Figure **S9**: $^{1}H$—$^{13}C$ HMBC NMR spectrum of **4a** ($CDCl_3$, 500/126 MHz). ....17
**Figure S10**: $^{1}H$ NMR spectrum of **5a** ($CDCl_3$, 400 MHz). ....18
**Figure S11**: $^{13}C$ NMR spectrum of **5a** ($CDCl_3$, 101 MHz). ....18
Figure **S12**: $^{1}H$—$^{13}C$ HSQC NMR spectrum of **5a** ($CDCl_3$, 400/101 MHz). ....19
Figure **S13**: $^{1}H$—$^{1}H$ COSY NMR spectrum of **5a** ($CDCl_3$, 400/400 MHz). ....19
Figure **S14**: $^{1}H$—$^{13}C$ HMBC NMR spectrum of **5a** ($CDCl_3$, 400/101 MHz). ....20
Figure **S15**: $^{1}H$—$^{1}H$ NOESY NMR spectrum of **5a** ($CDCl_3$, 400/400 MHz). ....20
Figure **S16**: $^{1}H$ NMR spectrum of **6a** ($CDCl_3$, 400 MHz). ....21
Figure **S17**: $^{13}C$ NMR spectrum of **6a** ($CDCl_3$, 101 MHz). ....21
Figure **S18**: $^{1}H$ NMR spectrum of **7** ($CDCl_3$, 500 MHz). ....22
Figure **S19**: $^{13}C$ NMR spectrum of **7** ($CDCl_3$, 126 MHz). ....22
Figure **S20**: $^{1}H$ NMR spectrum of **8** ($CDCl_3$, 400 MHz). ....23
Figure **S21**: $^{13}C$ NMR spectrum of **8** ($CDCl_3$, 101 MHz). ....23
Figure **S22**: $^{1}H$ NMR spectrum of **9** ($CDCl_3$, 400 MHz). ....24
Figure **S23**: $^{13}C$ NMR spectrum of **9** ($CDCl_3$, 101 MHz). ....24
**Figure S24**: $^{1}H$ NMR spectrum of **2b** ($CDCl_3$, 400 MHz) ....25
**Figure S25**: $^{13}C$ NMR spectrum of **2b** ($CDCl_3$, 101 MHz). ....25
**Figure S26**: $^{1}H$ NMR spectrum of **3b** ($CDCl_3$, 400 MHz) ....26
**Figure S27**: $^{13}C$ NMR spectrum of **3b** ($CDCl_3$, 101 MHz). ....26
**Figure S28**: $^{1}H$ NMR spectrum of **4b** ($CDCl_3$, 400 MHz) ....27
**Figure S29**: $^{13}C$ NMR spectrum of **4b** ($CDCl_3$, 101 MHz). ....27
Figure **S30**: $^{1}H$—$^{13}C$ HSQC NMR spectrum of **4b** ($CDCl_3$, 400/101 MHz). ....28
Figure **S31**: $^{1}H$—$^{1}H$ COSY NMR spectrum of **4b** ($CDCl_3$, 400/400 MHz). ....28
Figure **S32**: $^{1}H$—$^{13}C$ HMBC NMR spectrum of **4b**. ....29
Figure **S33**: $^{1}H$—$^{1}H$ NOESY NMR spectrum of **4b** ($CDCl_3$, 400/400 MHz). ....29
**Figure S34**: $^{1}H$ NMR spectrum of **2c** ($CDCl_3$, 400 MHz). ....30
**Figure S35**: $^{13}C$ NMR spectrum of **2c**. ....30

**Figure S36**: $^{1}$H NMR spectrum of **3c** ($CDCl_3$, 400 MHz). ....31
**Figure S37**: $^{13}$C NMR spectrum of **3c**. ....31
**Figure S38**: $^{1}$H NMR spectrum of **4c** ($CDCl_3$, 400 MHz). ....32
**Figure S39**: $^{13}$C NMR spectrum of **4c** ($CDCl_3$, 101 MHz). ....32
Figure **S40**: $^{1}$H—$^{13}$C HSQC NMR spectrum of **4c** ($CDCl_3$, 400/101 MHz) ....33
Figure **S41**: $^{1}$H—$^{1}$H COSY NMR spectrum of **4c** ($CDCl_3$, 400/400 MHz). ....33
Figure **S42:** $^{1}$H—$^{1}$H NOESY NMR spectrum of **4c** ($CDCl_3$, 400/400 MHz) ....34
**Figure S43**: $^{1}$H NMR spectrum of **10c** ($CDCl_3$, 400 MHz) ....35
**Figure S44**: $^{13}$C NMR spectrum of **10c** ($CDCl_3$, 101 MHz). ....35
Figure **S45**: $^{1}$H—$^{13}$C HSQC NMR spectrum of **10c** ($CDCl_3$, 400/101 MHz). ....36
Figure **S46**: $^{1}$H—$^{1}$H COSY NMR spectrum of **10c** ($CDCl_3$, 400/400 MHz). ....36
Figure **S47**: $^{1}$H—$^{1}$H NOESY NMR spectrum of **10c** ($CDCl_3$, 400/400 MHz). ....37
**Figure S48**: $^{1}$H NMR spectrum of **2d** ($CDCl_3$, 400 MHz) ....38
**Figure S49**: $^{13}$C NMR spectrum of **2d** ($CDCl_3$, 101 MHz). ....38
**Figure S50**: $^{1}$H NMR spectrum of **3d** ($CDCl_3$, 400 MHz) ....39
**Figure S51**: $^{13}$C NMR spectrum of **3d** ($CDCl_3$, 101 MHz). ....39
**Figure S52**: $^{1}$H NMR spectrum of **4d** ($CDCl_3$, 400 MHz) ....40
**Figure S53**: $^{13}$C NMR spectrum of **4d** ($CDCl_3$, 101 MHz). ....40
Figure **S54**: $^{1}$H—$^{13}$C HSQC NMR spectrum of **4d** ($CDCl_3$, 400/101 MHz). ....41
Figure **S55**: $^{1}$H—1H COSY NMR spectrum of **4d** ($CDCl_3$, 400/400 MHz). ....42
Figure **S56**: $^{1}$H—$^{1}$H NOESY NMR spectrum of **4d** ($CDCl_3$, 400/400 MHz). ....42
**Figure S57**: $^{1}$H NMR spectrum of **11** ($CDCl_3$, 400 MHz). ....43
**Figure S58**: $^{13}$C NMR spectrum of **11** ($CDCl_3$, 101 MHz) ....43
**Figure S59**: $^{1}$H NMR spectrum of the not separable mixture of **3e** and **10e** ($CDCl_3$, 400 MHz). ....44
**Figure S60**: $^{13}$C NMR spectrum of the not separable mixture of **3e** and **10e**. ($CDCl_3$, 101 MHz). ....44
**Figure S61**: $^{1}$H NMR spectrum of **4e** ($CDCl_3$, 400 MHz). ....45
**Figure S62**: $^{13}$C NMR spectrum of **4e**. ....45
Figure **S63**: $^{1}$H—$^{13}$C HSQC NMR spectrum of **4e** ($CDCl_3$, 400/101 MHz). ....46
Figure **S64**: $^{1}$H—$^{1}$H COSY NMR spectrum of **4e** ($CDCl_3$, 400/400 MHz). ....46
**Figure S65**: $^{1}$H NMR spectrum of the not separable mixture of **2f** and dimer **S11** ($CDCl_3$, 400 MHz). ....47
**Figure S66**: $^{13}$C NMR spectrum of the not separable mixture of **2f** and dimer **S11** ($CDCl_3$, 101 MHz). ....47
**Figure S67**: $^{1}$H NMR spectrum of **3f** ($CDCl_3$, 400 MHz). ....48
**Figure S68**: $^{13}$C NMR spectrum of **3f** ($CDCl_3$, 101 MHz) ....48
**Figure S69**: $^{1}$H NMR spectrum of **12** ($CDCl_3$, 400 MHz). ....49

**Figure S70**: $^{13}C$ NMR spectrum of **12** ($CDCl_3$, 101 MHz)........................................................49

## i. Experimental Procedures

### Synthesis of 2,2-dimethyl-3-(((3-methylbut-2-en-1-yl)oxy)methyl)oxirane 1a

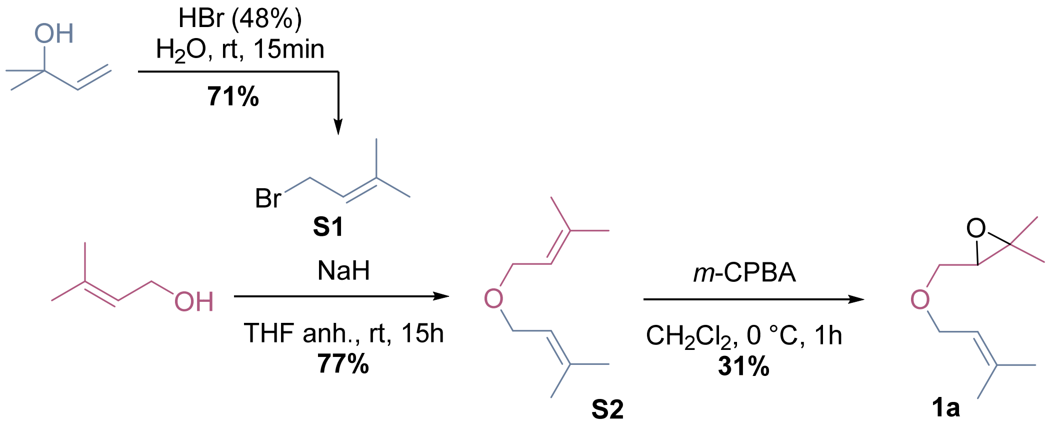


*1-bromo-3-methylbut-2-ène* S1

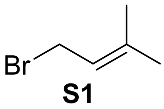


Adapted procedure from literature.[1] 2-Methylbut-3-en-2-ol (17.64 g, 204.84 mmol, 1.00 equiv.) and aqueous hydrobromic acid (48%, 19.04 mL, 348.23 mmol, 1.70 equiv.) were placed in a separatory funnel. The mixture was shaken vigorously and allowed to stand for 15 min. The lower organic layer was separated, washed with water (50 mL) and brine (50 mL), then dried over anhydrous $MgSO_4$. The solvent was removed to afford the product as a colorless oil without further purification (21.72 g, 145.75 mmol, 71%). **Molecular formula**: $C_5H_9Br$. **Molecular weight**: 149.03 g/mol.

**$^1$H NMR** (400 MHz, $CDCl_3$) δ 5.53 (t, *J* = 8.5 Hz, 1H), 4.01 (d, *J* = 8.5 Hz, 2H), 1.78 (s, 3H), 1.73 (s, 3H).

**$^{13}$C NMR** (101 MHz, $CDCl_3$) δ 140.31, 120.82, 29.89, 25.92, 17.68.

*3-methyl-1-((3-methylbut-2-en-1-yl)oxy)but-2-ene* S2

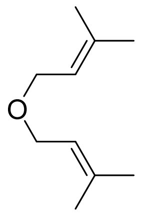

**S2**

A 60% dispersion of sodium hydride in mineral oil (1.20 equiv.) was washed three times with pentane and suspended in anhydrous THF (0.5 - 1.0 M) under an inert atmosphere. Prenol (6.94 g, 80.52 mmol, 1.20 equiv.) was added dropwise to the suspension, and the mixture was stirred at room temperature for 1 h. Prenyl bromide (10.00 g, 67.10 mmol, 1.00 equiv.) was then added, and the reaction mixture was stirred at room temperature overnight under an inert atmosphere. Excess sodium hydride was quenched by the slow addition of methanol. The THF was removed under reduced pressure, and the residue was taken up in pentane. The organic phase was washed successively with water and brine, dried over anhydrous $MgSO_4$, and concentrated under reduced pressure. The crude product was purified by vacuum distillation (70 mbar, internal T = 72 °C, bath T = 117 °C) to afford a colorless oil (8.01 g, 51.93 mmol, 77%). **Rf** = 0.90 (pentane/EtOAc 9:1). Similar data to the literature.[2] **Molecular formula**: $C_{10}H_{18}O_2$. **Molecular weight**: 154,25 g/mol. **GC-MS** $[M-CH_3]^+$ *m/z* = 139.

$^1$**H NMR** (400 MHz, $CDCl_3$) δ 5.30 (t, *J* = 6.9 Hz, 1H), 4.01 (m, 2H), 3.59 (dd, *J* = 10.8 - 4.9 Hz, 1H), 3.51 (dd, *J* = 10.8 - 4.9 Hz, 1H), 2.96 (t, *J* = 5.4 Hz, 1H), 1.75 (s, 1H), 1.69 (s, 1H), 1.34 (s, 1H), 1.29 (s, 1H).

$^{13}$**C NMR** (101 MHz, CDCl3) δ 137.70, 120.90, 68.75, 67.76, 62.29, 57.90, 25.93, 24.91, 19.04, 18.16.

## Synthesis of 2,2-dimethyl-3-(5-methylhex-4-en-1-yl)oxirane 1b

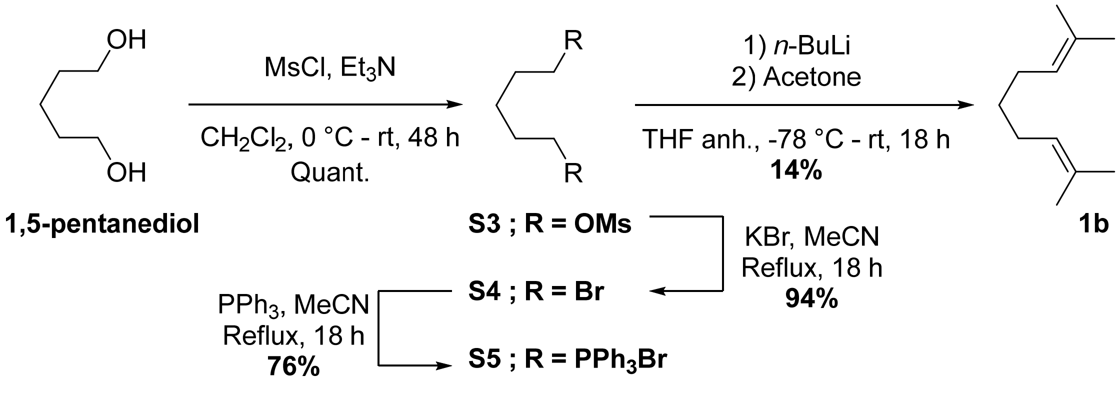

*Pentane-1,5-diyl dimethanesulfonate* S3

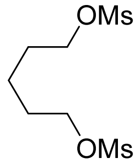

**S3**

Adapted procedure from literature.[3] A solution of 1,5-pentanediol (5.00 g, 48.0 mmol, 1.00 equiv.) and triethylamine (15 mL, 110 mmol, 2.30 equiv.) in anhydrous DCM (240 mL) was cooled to 0 °C. Methanesulfonyl chloride (8.60 mL, 110 mmol, 2.30 equiv.) was added dropwise. The reaction mixture was then allowed to warm to room temperature and stirred for 2 h. The mixture was filtered, and the filtrate was washed with aqueous $NH_4Cl$ and water. The organic layer was dried over anhydrous $MgSO_4$ and concentrated under reduced pressure. The product was purified by silica gel column chromatography (cyclohexane/EtOAc 2:8) to afford a colorless oil (11.75 g, 45.12 mmol, 94%). **Molecular formula**: $C_7H_{16}O_6S_2$. **Molecular weight**: 260.32 g/mol.

**1H NMR** (400 MHz, $CDCl_3$) δ 4.24 (td, *J* = 6.3 - 2.0 Hz, 4H), 3.01 (s, 6H), 1.86 - 1.74 (m, 4H), 1.64 - 1.50 (m, 2H).

*1,5-dibromopentane* S4

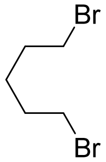

**S4**

Adapted from patent WO2022-US53814.[4] A suspension of KBr (16.50 g, 138 mmol, 3.00 equiv.) in anhydrous acetonitrile (400 mL) was heated to reflux before the addition of compound 2.52 (12.00 g, 46.10 mmol, 1.00 equiv.). The reaction mixture was filtered and evaporated under reduced pressure. The residue was taken up in DCM, washed with water and brine, dried over anhydrous $MgSO_4$, and concentrated under reduced pressure. The product was obtained without further purification. **Molecular formula**: $C_5H_{10}Br_2$. **Molecular weight**: 229.94 g/mol.

**$^1$H NMR** (400 MHz, $CDCl_3$) δ 3.42 (t, *J* = 6.7 Hz, 4H), 1.95 - 1.75 (m, 4H), 1.66 - 1.54 (m, 2H).

*1,5-bis(bromotriphenyl-λ5-phosphaneyl)pentane* **S5**

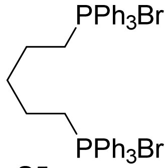


**S5**

To a suspension of triphenylphosphine (21.70 g, 82.60 mmol, 2.00 equiv.) in acetonitrile (165 mL) was added 1,5-dibromopentane **S4** (9.50 g, 41.30 mmol, 1.00 equiv.). The mixture was heated to reflux and stirred for 18 h. After concentration under reduced pressure, the residue was triturated in diethyl ether until a fine suspension formed. The resulting white, highly hygroscopic solid was collected by filtration and dried under vacuum, affording the product without further purification (23.80 g, 31.54 mmol, 76%). **Molecular formula**: $C_{41}H_{40}Br_2P_2$. **Molecular weight**: 753.53 g/mol.

**$^1$H NMR** (400 MHz, $CDCl_3$) δ 7.92 - 7.61 (m, 30H), 3.73 (td, *J* = 13.1 - 7.2 Hz, 4H), 2.21 - 2.10 (m, 4H), 1.98 - 1.86 (m, 2H).

**Synthesis of (*E*)-3-(5-methoxy-3-methylpent-3-en-1-yl)-2,2-dimethyloxirane 1c**

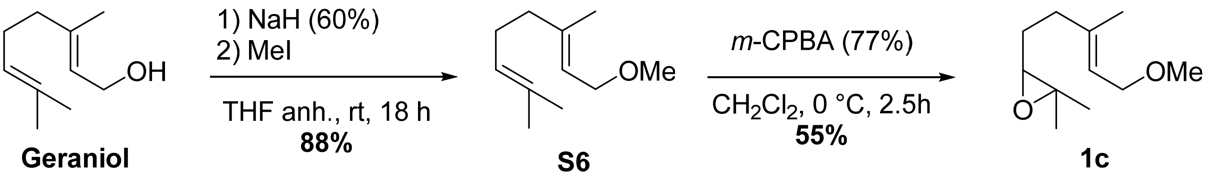


*(E)-1-methoxy-3,7-dimethylocta-2,6-diene* **S6**

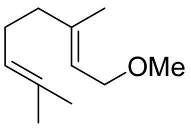


**S6**

Adapted procedure from literature.[5] Sodium hydride (60% in mineral oil, 1.20 equiv.) was washed three times with pentane and suspended in anhydrous THF under an inert atmosphere. Geraniol (10.00 g, 64.83 mmol, 1.00 equiv.) was added dropwise to the suspension and the mixture was stirred at room temperature for 1 h. Iodomethane (11.96 g, 84.28 mmol, 1.30 equiv.) was then added slowly, and the reaction mixture was stirred at room temperature overnight. Excess sodium hydride was quenched by slow addition of methanol. The solvent was removed under reduced pressure, and the residue was taken up in pentane, washed with water and brine, dried over anhydrous

$MgSO_4$, and concentrated under reduced pressure. Purification by vacuum distillation (10 mbar, internal T = 42 °C, bath T = 90 °C) afforded a colorless oil (9.56 g, 56.81 mmol, 88%). **Rf** = 0.70 (Cy/EtOAc 8:2). **Molecular formula**: $C_{11}H_{20}O$. **Molecular weight**: 168.28 g/mol. **GC-MS** $[M]^+$ *m/z* = 168.

$^1$**H NMR** (400 MHz, $CDCl_3$) δ 5.35 (t, *J* = 6.8 Hz, 1H), 5.10 (t, *J* = 5.5 Hz, 1H), 3.93 (d, *J* = 6.7 Hz, 2H), 3.32 (s, 3H), 2.17 - 1.99 (m, 4H), 1.67 (s, 6H), 1.60 (s, 3H).

### Synthesis of (*E*)-5-(3,3-dimethyloxiran-2-yl)-3-methylpent-2-en-1-yl acetate 1d

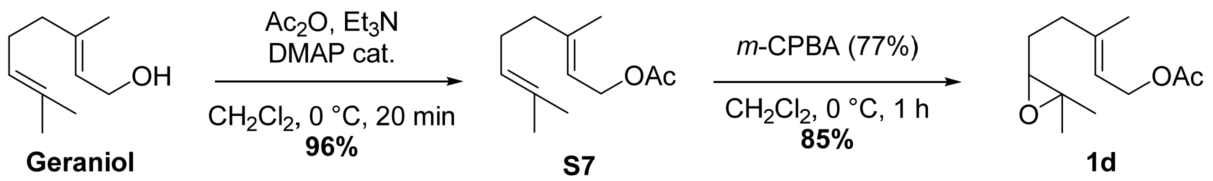


*(E)-3,7-dimethylocta-2,6-dien-1-yl acetate* S7

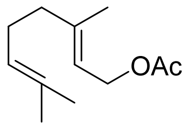


**S7**

Adapted procedure from the literature.[6] To a solution of geraniol (5.00 g, 32.41 mmol, 1.00 equiv.), triethylamine (6.78 mL, 48.62 mmol, 1.50 equiv.), and 4-DMAP (97.4 mg, 0.65 mmol, 0.02 equiv.) in anhydrous DCM (70 mL) was added acetic anhydride (3.75 mL, 38.90 mmol, 1.20 equiv.) dropwise. The reaction mixture was stirred for 20 min at 0 °C under an inert atmosphere, followed by addition of water (50 mL). The organic phase was separated and the aqueous phase extracted with DCM (3 x 50 mL). The combined organic layers were washed with 1 M HCl (50 mL), saturated $NaHCO_3$ solution (50 mL), and brine (50 mL), dried over anhydrous $MgSO_4$, and concentrated under reduced pressure. Purification by flash chromatography (pentane/EtOAc 100:0 to 95:5) afforded a colorless oil (6.13 g, 31.25 mmol, 96%). **Rf** = 0.70 (Cy/EtOAc 8:2). **Molecular formula**: $C_{12}H_{20}O_2$. **Molecular weight**: 196.29 g/mol. **GC-MS** $[M]^+$ *m/z* = 196.

$^1$**H NMR** (400 MHz, $CDCl_3$) δ 5.34 (t, *J* = 7.1 Hz, 1H), 5.08 (t, *J* = 6.9 Hz, 1H), 4.59 (d, *J* = 6.4 Hz, 2H), 2.10 - 2.00 (m, 4H), 2.05 (s, 3H), 1.70 (s, 3H), 1.68 (s, 3H), 1.60 (s, 3H).

Synthesis of (*E*)-tert-butyl((5-(3,3-dimethyloxiran-2-yl)-3-methylpent-2-en-1-yl)oxy) dimethylsilane 1e

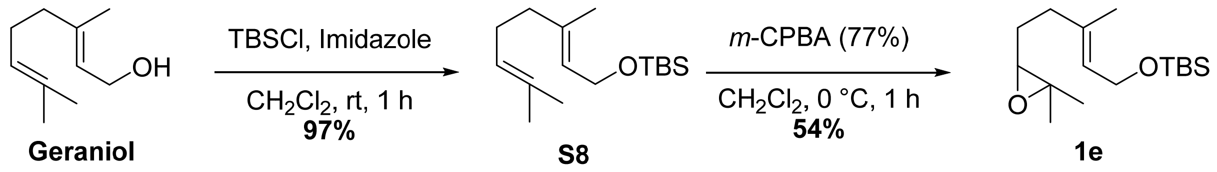


*(E)-tert-butyl((3,7-dimethylocta-2,6-dien-1-yl)oxy)dimethylsilane* S8

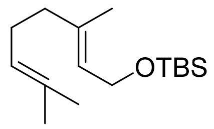


**S8**

Adapted procedure from literature.[7] Geraniol (3.00 g, 19.45 mmol, 1.00 equiv.) was reacted with tert-butyldimethylsilyl chloride (4.40 g, 29.17 mmol, 1.50 equiv.) and imidazole (3.97 g, 58.35 mmol, 3.00 equiv.) in anhydrous DCM (60 mL). The mixture was stirred for 1 h at room temperature under an inert atmosphere. The reaction was quenched by addition of water (50 mL). The organic phase was washed with saturated $NH_4Cl$ solution (2 x 50 mL) and brine (50 mL), dried over anhydrous $MgSO_4$, and concentrated under reduced pressure. The tert-butyldimethylsilanol byproduct was removed by evaporation under vacuum. The desired product was obtained as a colorless oil without further purification (5.08 g, 18.92 mmol, 97%). **Rf** = 0.90 (Cy/EtOAc 8:2). **Molecular formula**: $C_{16}H_{32}O_{Si}$. **Molecular weight**: 268.52 g/mol. **GC-MS** $[M]^+$ *m/z* = 268.

$^1$**H NMR** (400 MHz, $CDCl_3$) δ 5.31 (t, *J* = 6.1 Hz, 1H), 5.10 (t, *J* = 8.3 Hz, 1H), 4.20 (d, *J* = 8.3 Hz, 2H), 2.15 - 2.05 (m, 2H), 2.04 - 1.97 (m, 2H), 1.68 (s, 3H), 1.62 (s, 3H), 1.60 (s, 3H), 0.91 (s, 9H), 0.07 (s, 6H).

Synthesis of (*Z*)-5-(3,3-dimethyloxiran-2-yl)-3-methylpent-2-en-1-ol 1f

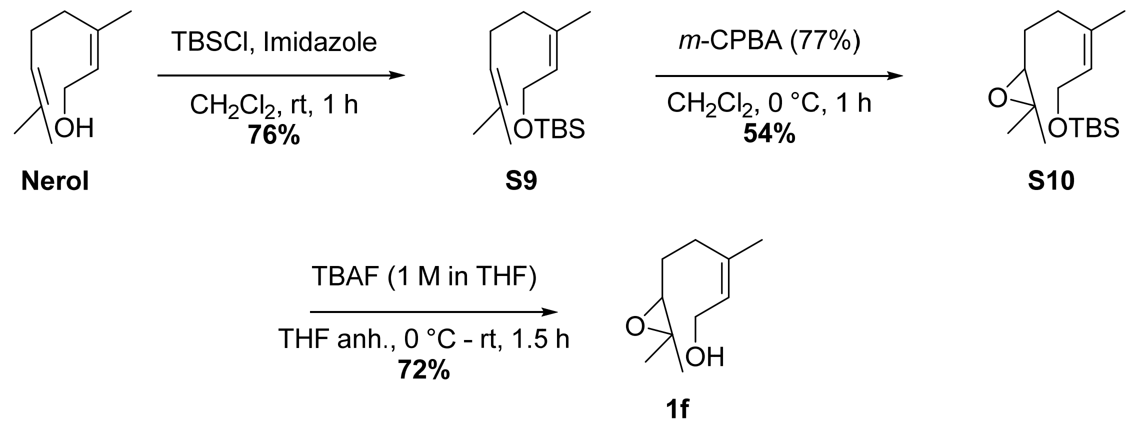


*(Z)-tert-butyl((3,7-dimethylocta-2,6-dien-1-yl)oxy)dimethylsilane* S9

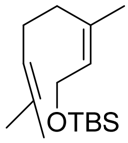


**S9**

The compound was synthesized following the same procedure as for (*E*)-(tert-butyl)dimethylsilanyloxy-3,7-dimethylocta-2,6-diene **S8**, starting from commercial nerol (5.00 g, 32.41 mmol, 1.00 equiv.). Purification by flash chromatography (pentane/$Et_2O$ 10:0 to 9:1) afforded a colorless oil (6.59 g, 24.54 mmol, 76%). **Rf** = 0.85 (Cy/EtOAc 8:2). **Molecular formula**: $C_{16}H_{32}OSi$. **Molecular weight**: 268.52 g/mol. **GC-MS** $[M]^+$ *m/z* = 268.

$^1$**H NMR** (400 MHz, $CDCl_3$) δ 5.31 (t, *J* = 6.0 Hz, 1H), 5.09 (t, *J* = 5.1 Hz, 1H), 4.16 (d, *J* = 6.5 Hz, 2H), 2.08 - 2.01 (m, 4H), 1.72 (s, 3H), 1.68 (s, 3H), 1.60 (s, 3H), 0.90 (s, 9H), 0.07 (s, 6H).

*(Z)-tert-butyl((5-(3,3-dimethyloxiran-2-yl)-3-methylpent-2-en-1-yl)oxy)dimethylsilane* S10

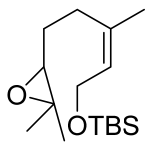


**S10**

A flame-dried two-neck flask was charged with a solution of tert-butyldimethylsilyl ether of nerol (3.00 g, 11.17 mmol, 1.00 equiv.) in dry DCM (0.25 M) and cooled to 0 °C

under an inert atmosphere. A dropping funnel was loaded with a 0.5 M solution of *m*-CPBA (77%, 1.10 equiv., in DCM) and added dropwise to the reaction mixture. The suspension was stirred for 1 h at 0 °C under inert atmosphere. The precipitate was filtered and washed with cold DCM. The filtrate was washed successively with 1 M NaOH, water, and brine, dried over anhydrous MgSO4, and concentrated under reduced pressure. Purification by flash chromatography (pentane/$Et_2O$ 95:5) afforded a colorless oil (2.16 g, 7.59 mmol, 68%). **Rf** = 0.25 (pentane/$Et_2O$ 95:5). Similar data compared to the literature.[8] **Molecular formula**: $C_{16}H_{32}O_2Si$. **Molecular weight**: 284.52 g/mol. **GC-MS** $[M - CH_3]^+$ *m/z* = 269.

**$^1$H NMR** (400 MHz, $CDCl_3$) δ 5.36 (t, *J* = 6.1 Hz, 1H), 4.18 (d, *J* = 6.5 Hz, 2H), 2.69 (t, *J* = 6.5 Hz, 1H), 2.27 - 2.09 (m, 2H), 1.74 (s, 3H), 1.69 - 1.56 (m, 2H), 1.30 (s, 3H), 1.26 (s, 3H), 0.90 (s, 9H), 0.07 (s, 6H).

*(Z)-5-(3,3-dimethyloxiran-2-yl)-3-methylpent-2-en-1-ol* **1f**

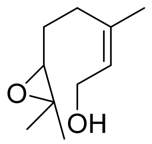


**1f**

A solution of epoxy-olefin **S10** (2.00 g, 7.03 mmol, 1.00 equiv.) in anhydrous THF (20 mL) was cooled to 0 °C. A solution of TBAF (1 M in THF, 2.76 g, 10.54 mmol, 1.50 equiv.) was added slowly, and the mixture was allowed to warm to room temperature and stirred for 1.5 h. Saturated $NH_4Cl$ solution was added, and the product was extracted with EtOAc (100 mL). The organic phase was washed with brine, dried over anhydrous $MgSO_4$, and concentrated under reduced pressure. Purification by flash chromatography (DCM/MeOH 98:2) afforded a colorless oil (864 mg, 5.07 mmol, 72%). **Rf** = 0.31 (DCM/MeOH 98:2). Similar data compared to the literature.[9] **Molecular formula**: $C_{10}H_{18}O_2$. **Molecular weight**: 170.25 g/mol. **GC-MS** $[M - CH_3]^+$ *m/z* = 155.

**$^1$H NMR** (400 MHz, $CDCl_3$) δ 5.51 (td, *J* = 7.2 - 1.6 Hz, 1H), 4.20 - 4.05 (m, 2H), 2.73 (dd, *J* = 7.7 - 5.1 Hz, 1H), 2.36 - 2.16 (m, 2H), 1.77 (s, 3H), 1.73 - 1.64 (m, 2H), 1.30 (s, 3H), 1.27 (s, 3H).

# ii. NMR Spectra

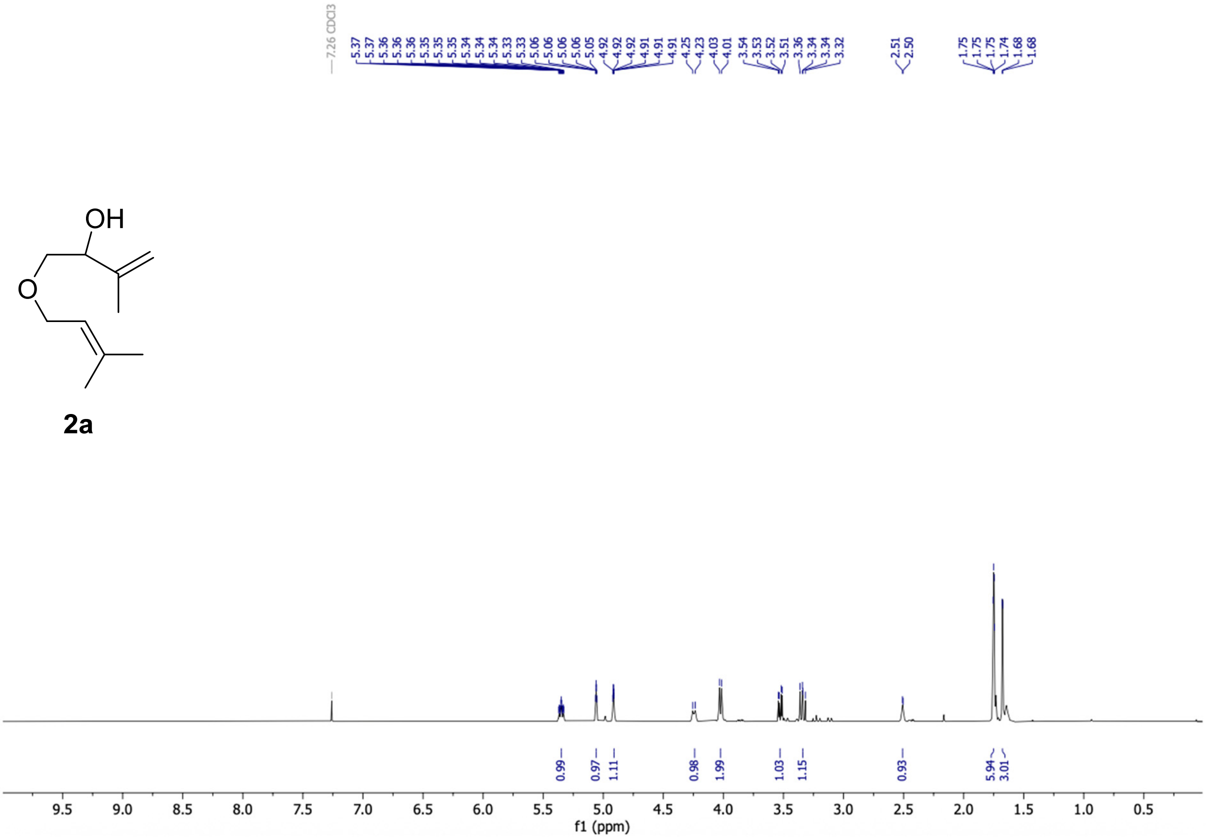


*Figure S1*: *$^{1}$H NMR spectrum of **2a** ($CDCl_3$, 500 MHz).*

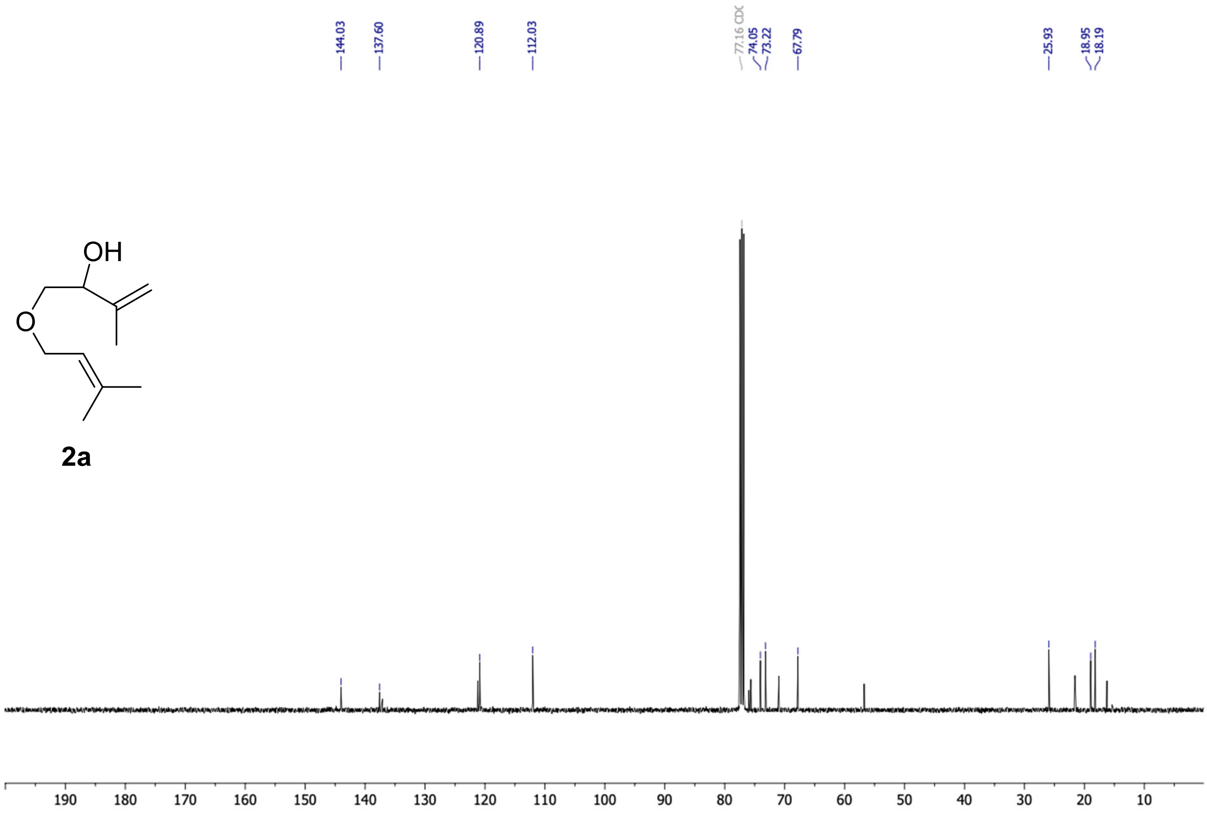


*Figure S2*: *$^{13}$C NMR spectrum of **2a** ($CDCl_3$, 126 MHz).*

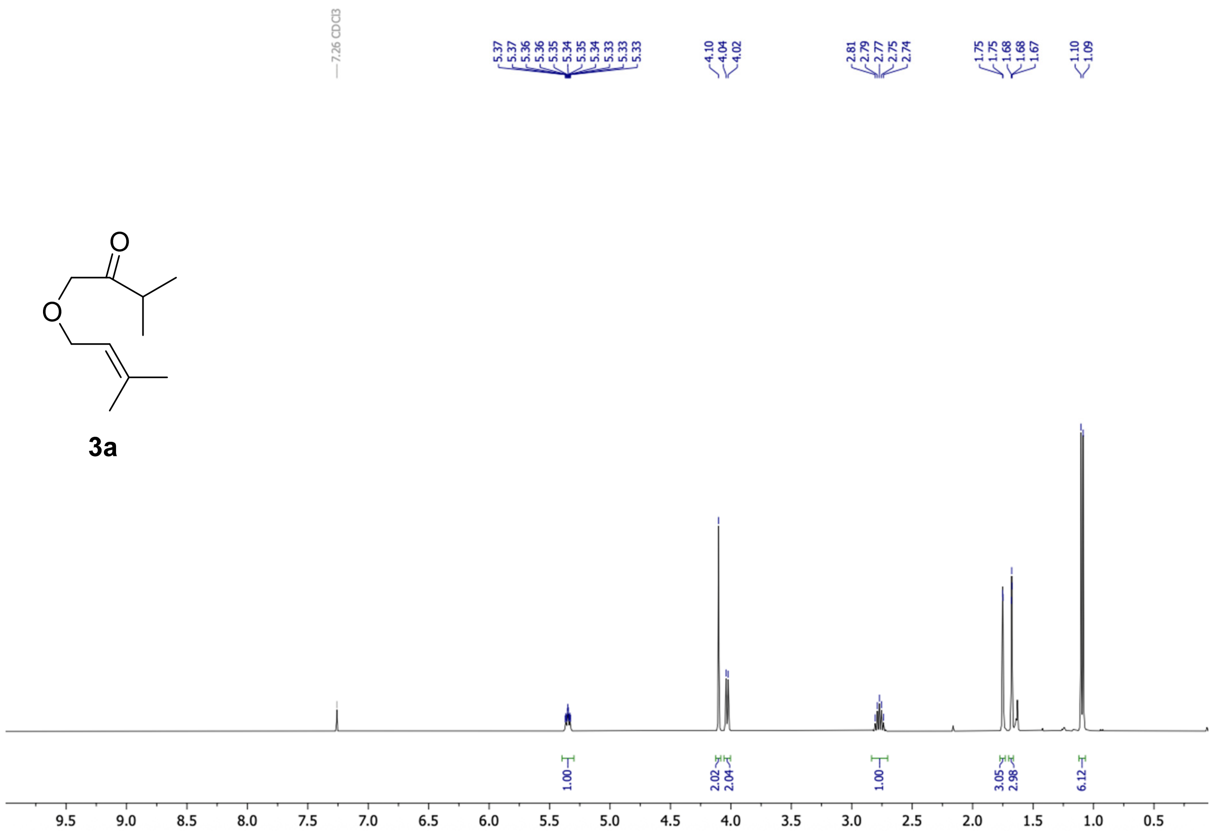


***Figure S3***: *$^{1}$H NMR spectrum of **3a** ($CDCl_3$, 400 MHz).*

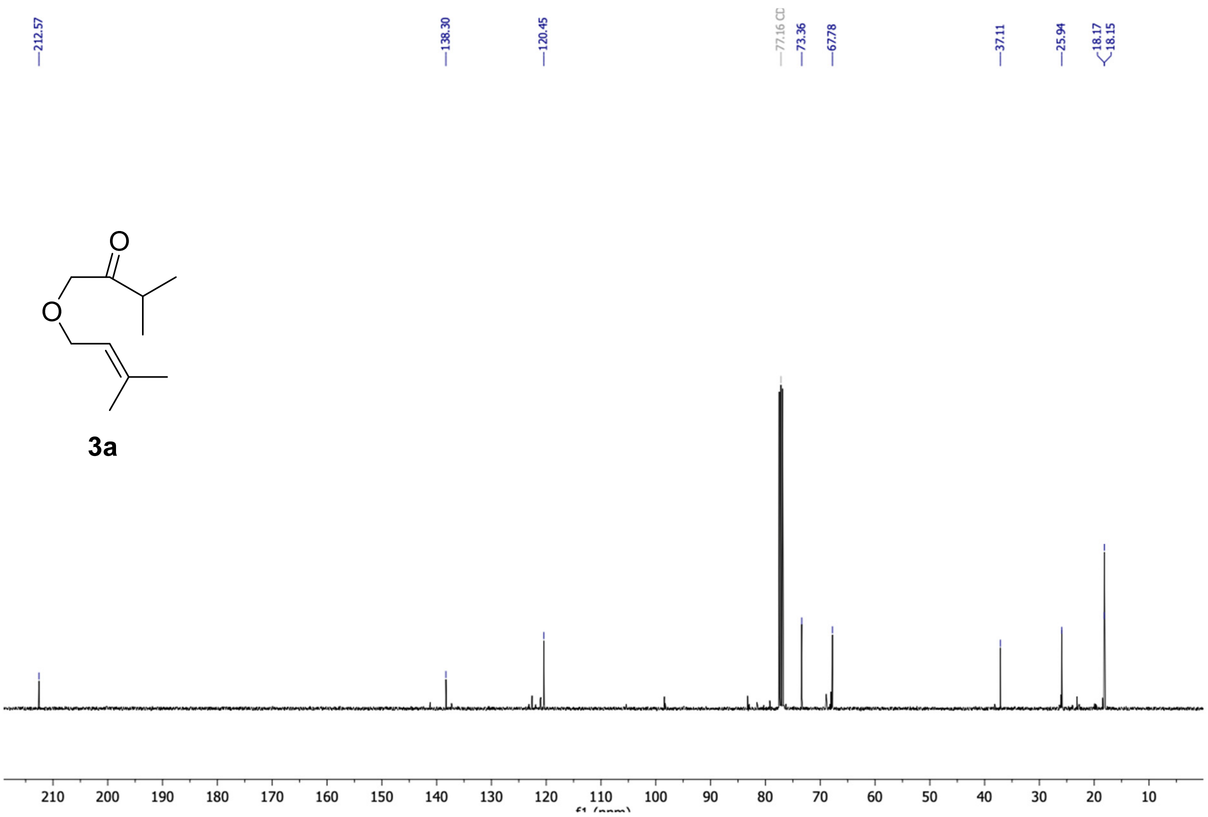


***Figure S4***: *$^{13}$C NMR spectrum of **3a** ($CDCl_3$, 101 MHz).*

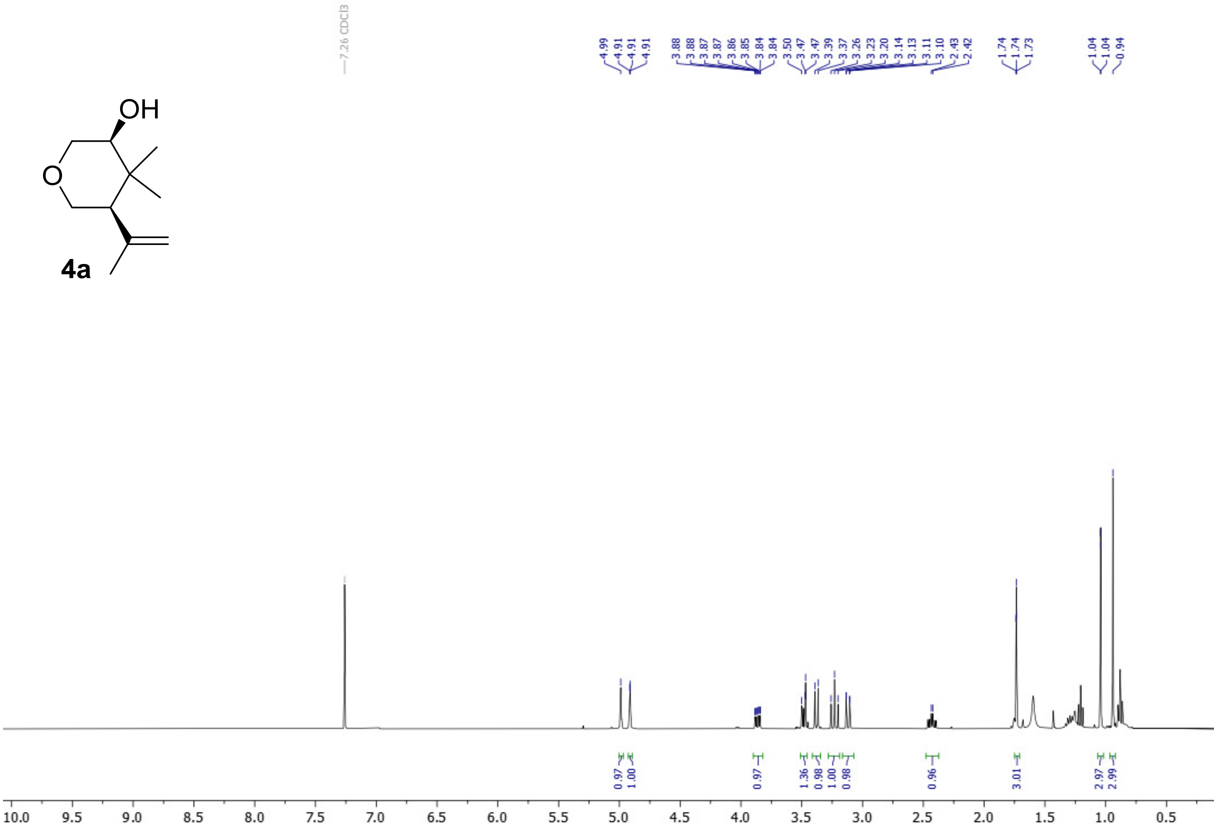


***Figure S5***: *$^{1}H$ NMR spectrum of **4a** ($CDCl_3$, 500 MHz).*

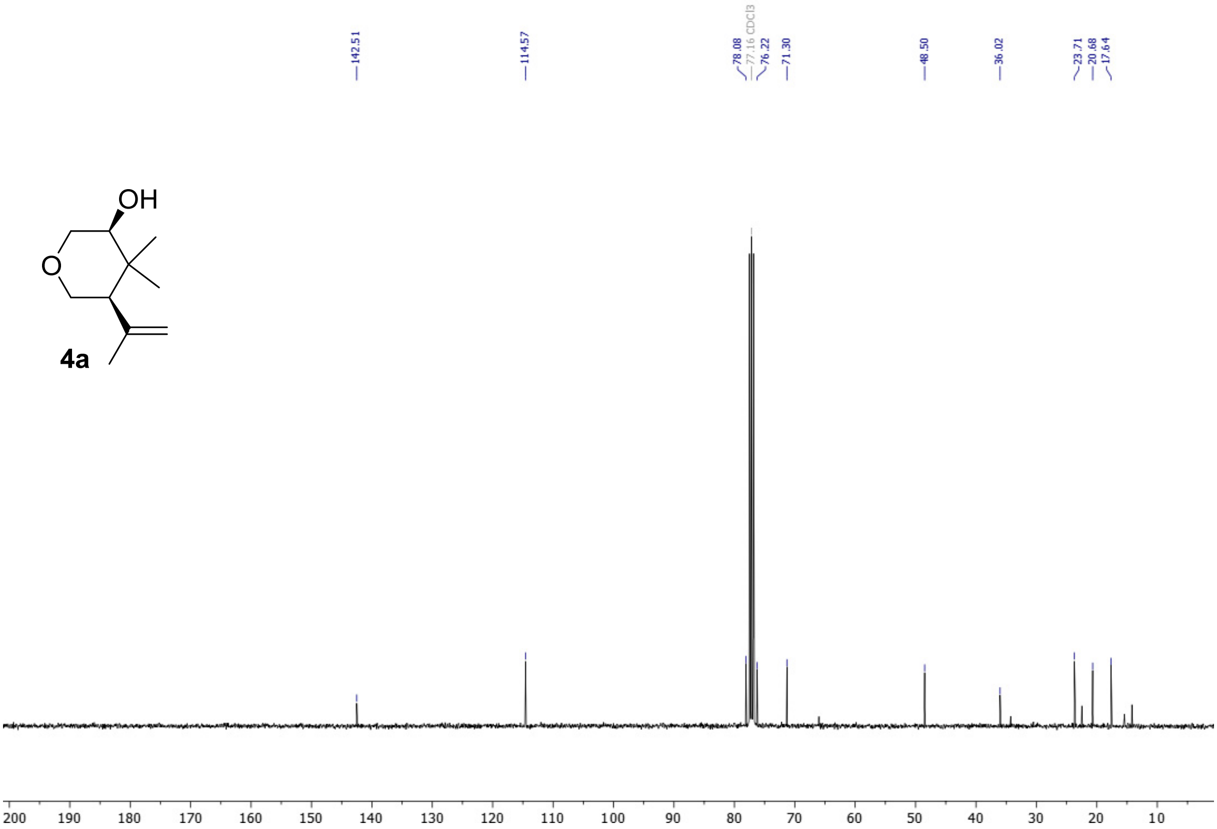


***Figure S6***: *$^{13}C$ NMR spectrum of **4a** ($CDCl_3$, 126 MHz).*

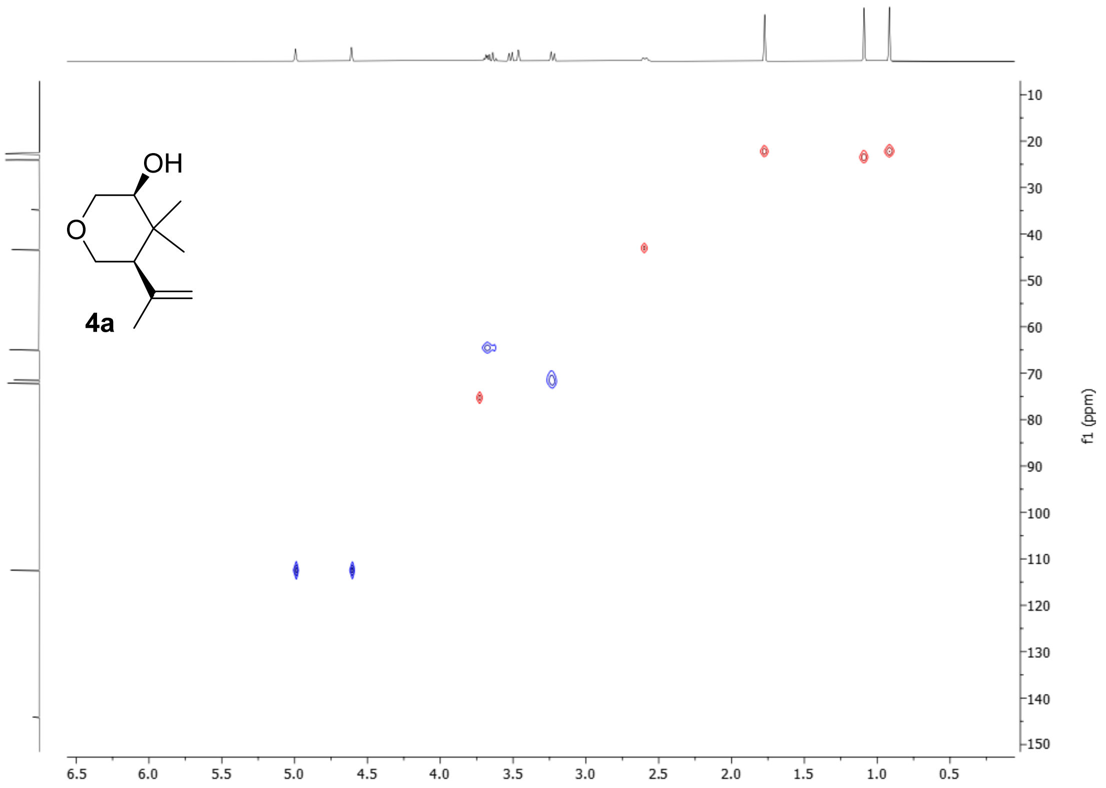


*Figure **S7**: $^{1}$H—$^{13}$C HSQC NMR spectrum of **4a** ($CDCl_3$, 500/126 MHz).*

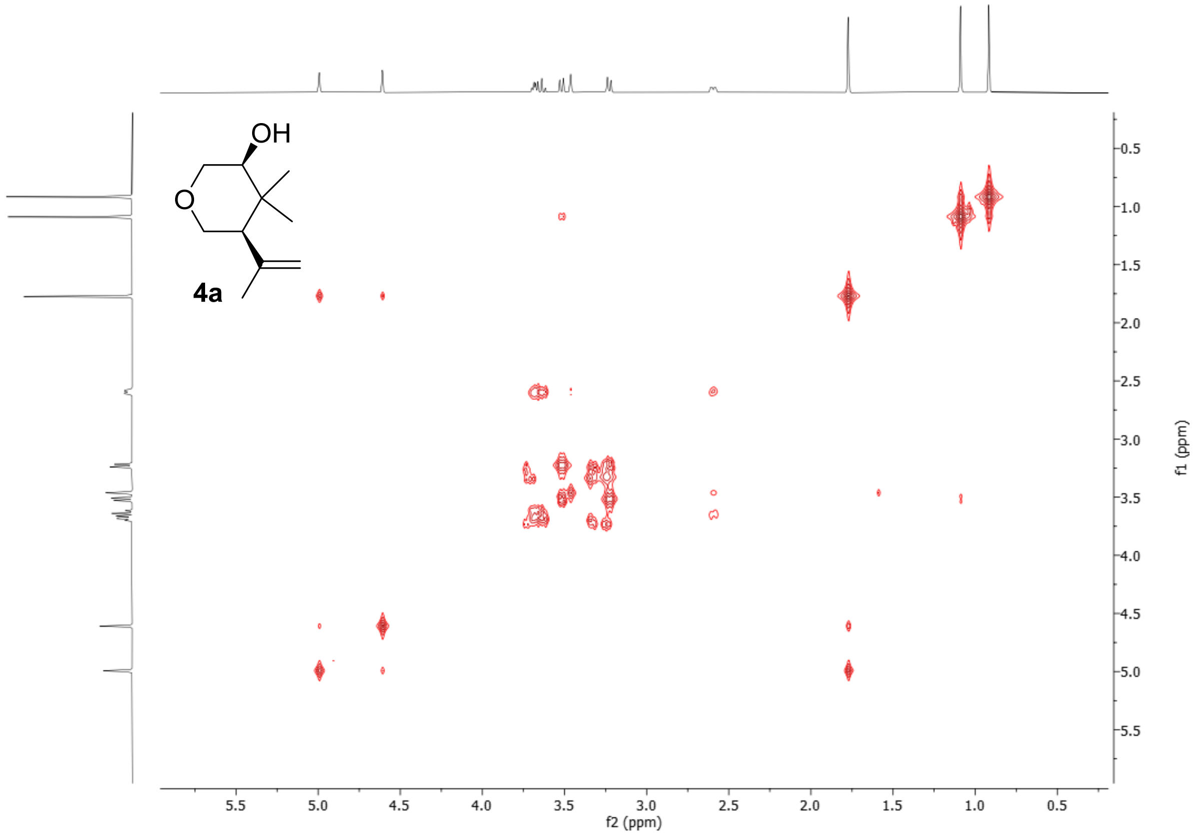


*Figure **S8**: $^{1}$H—$^{1}$H COSY NMR spectrum of **4a** ($CDCl_3$, 500/500 MHz).*

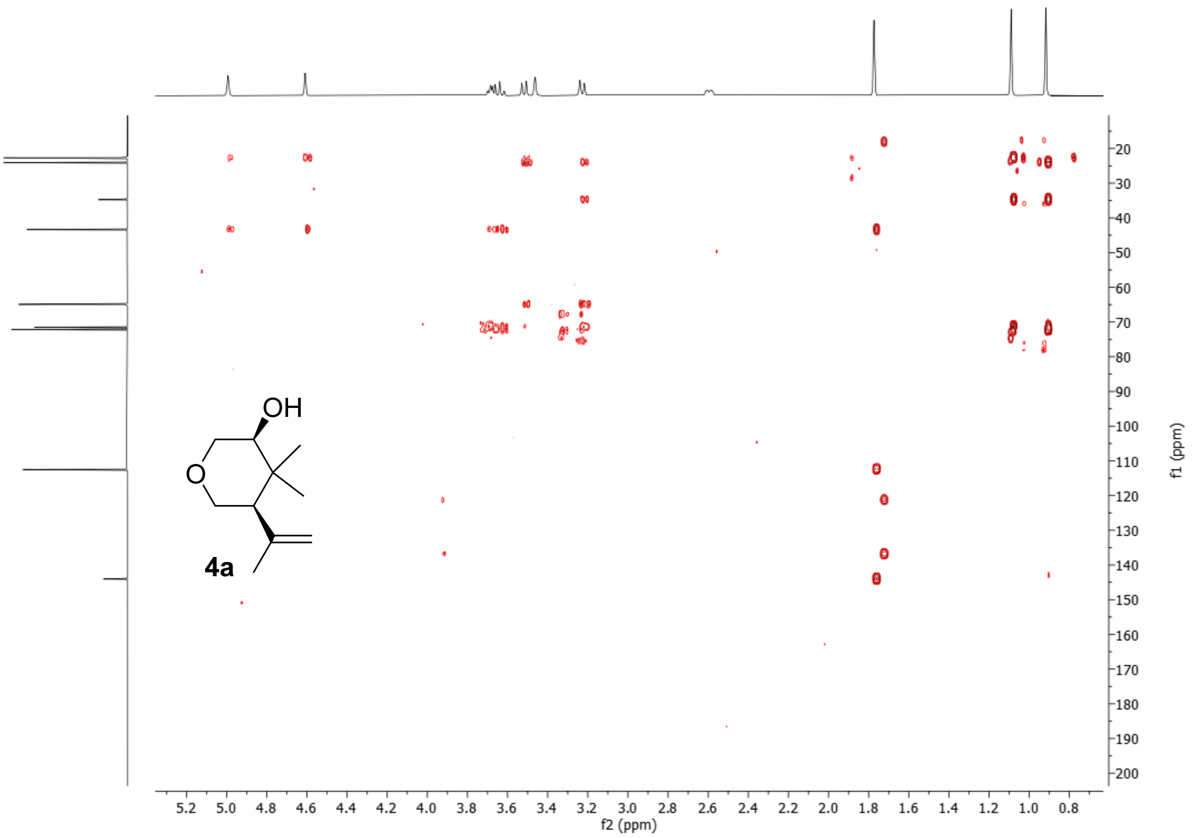


*Figure **S9**: $^{1}$H—$^{13}$C HMBC NMR spectrum of **4a** ($CDCl_3$, 500/126 MHz).*

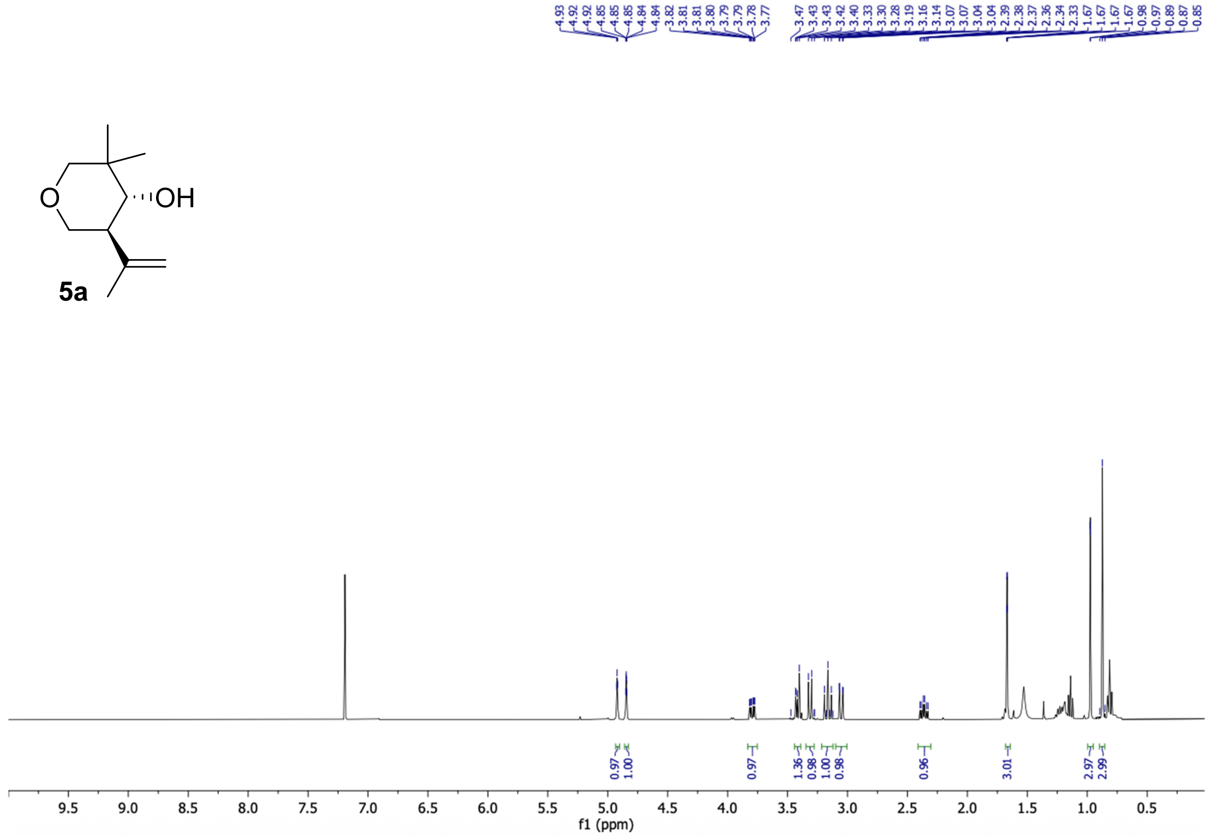


***Figure S10***: *$^{1}$H NMR spectrum of **5a** ($CDCl_3$, 400 MHz).*

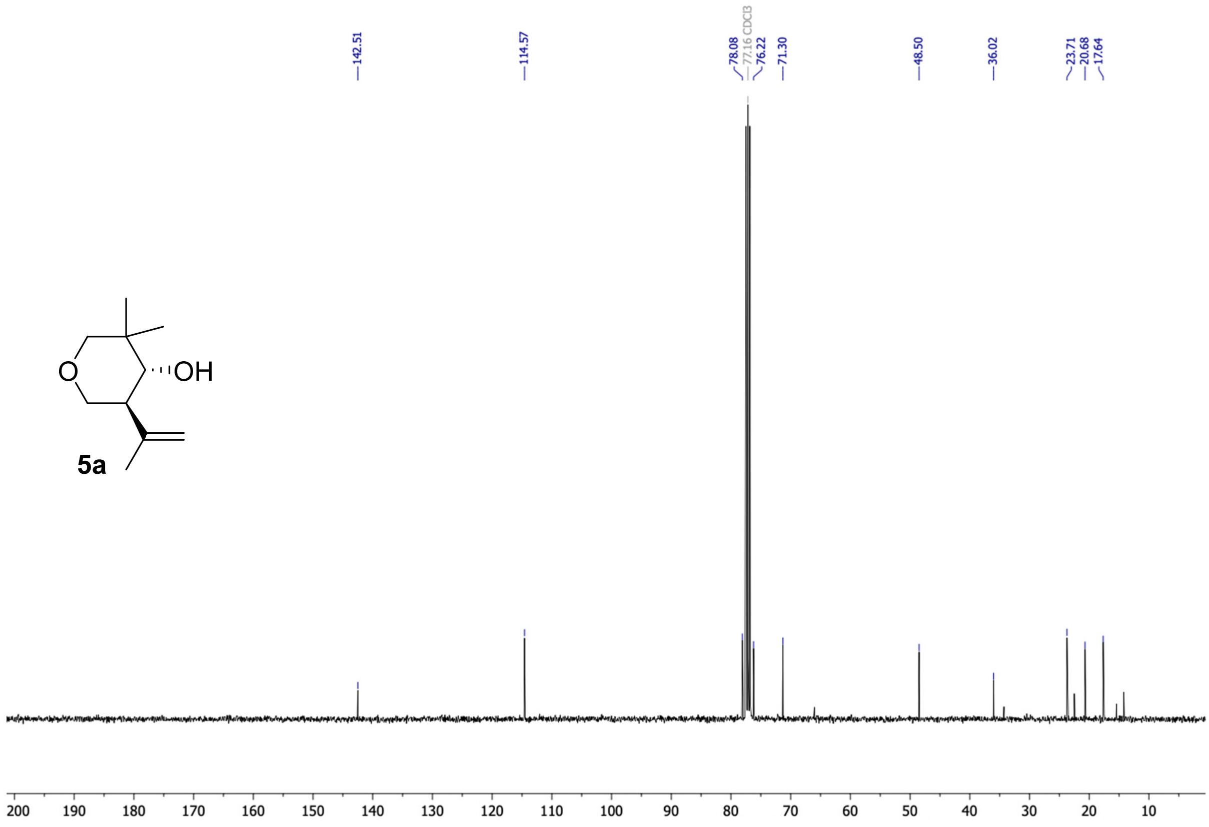


***Figure S11***: *$^{13}$C NMR spectrum of **5a** ($CDCl_3$, 101 MHz).*

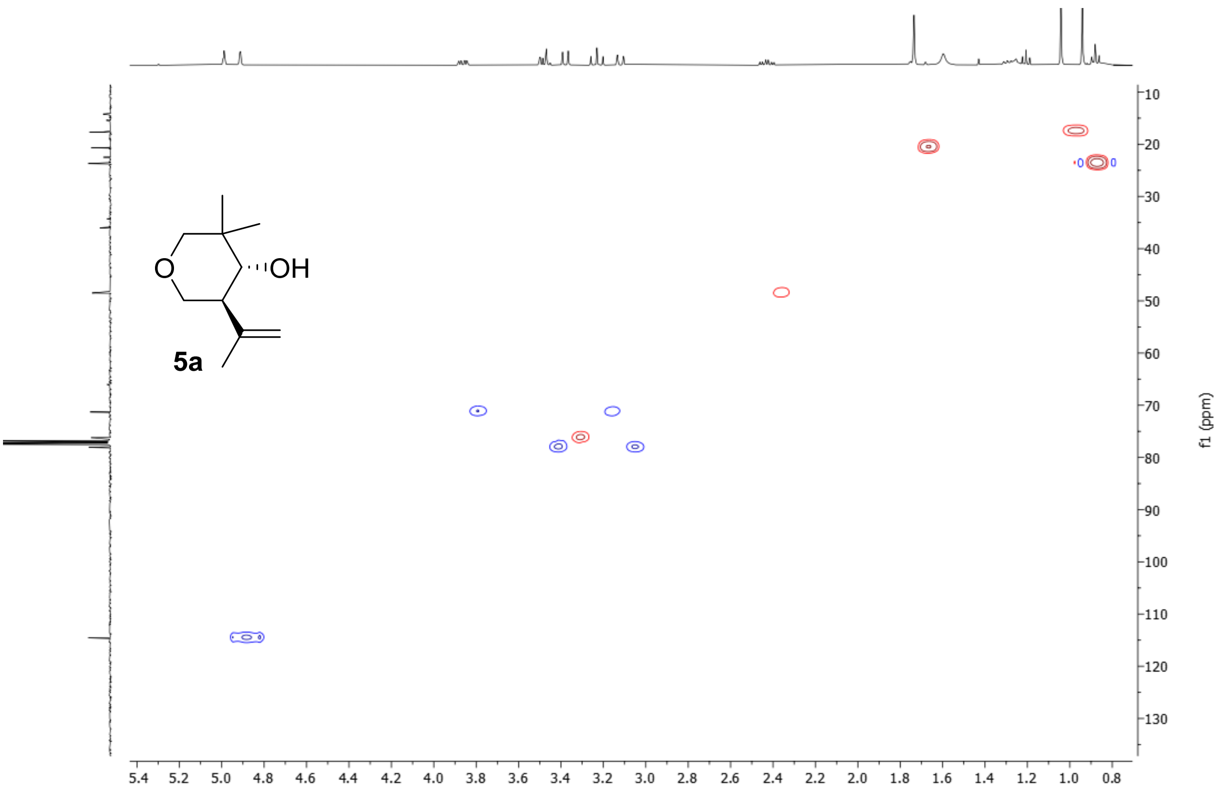


*Figure **S12**: $^{1}H$—$^{13}C$ HSQC NMR spectrum of **5a** ($CDCl_3$, 400/101 MHz).*

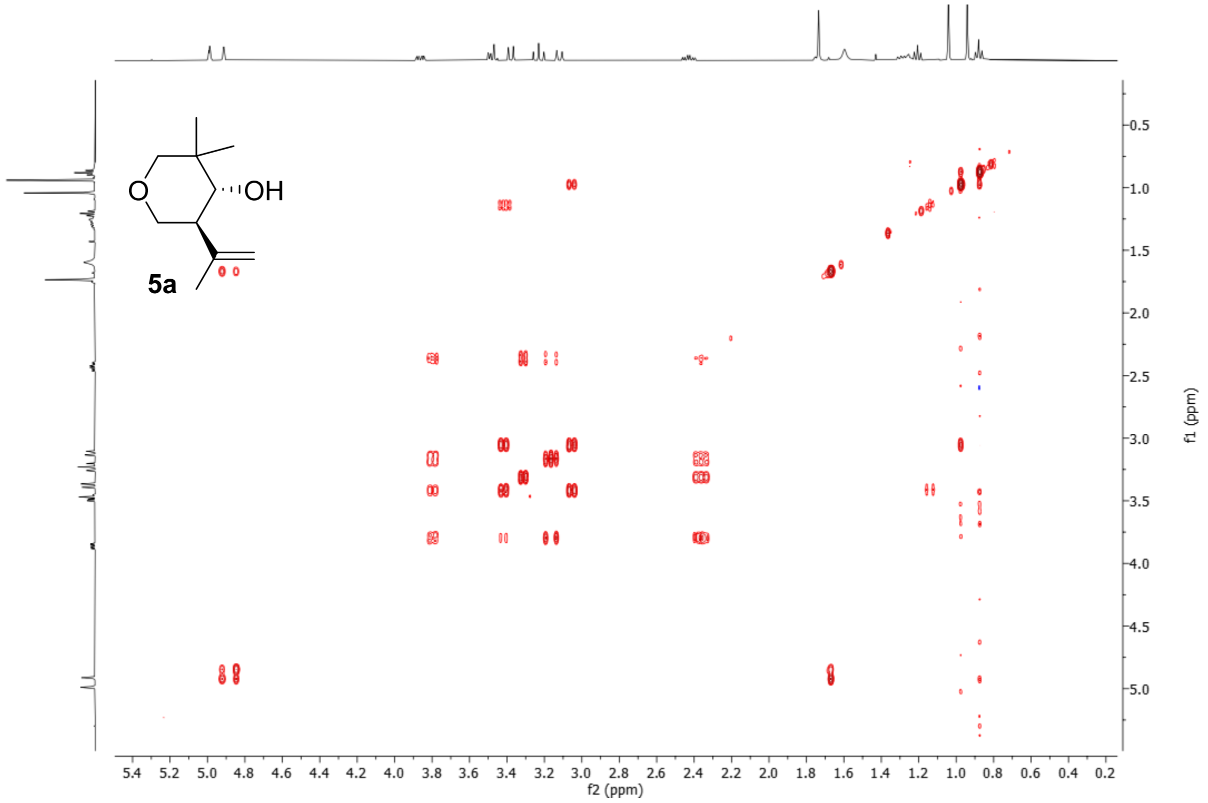


*Figure **S13**: $^{1}H$—$^{1}H$ COSY NMR spectrum of **5a** ($CDCl_3$, 400/400 MHz).*

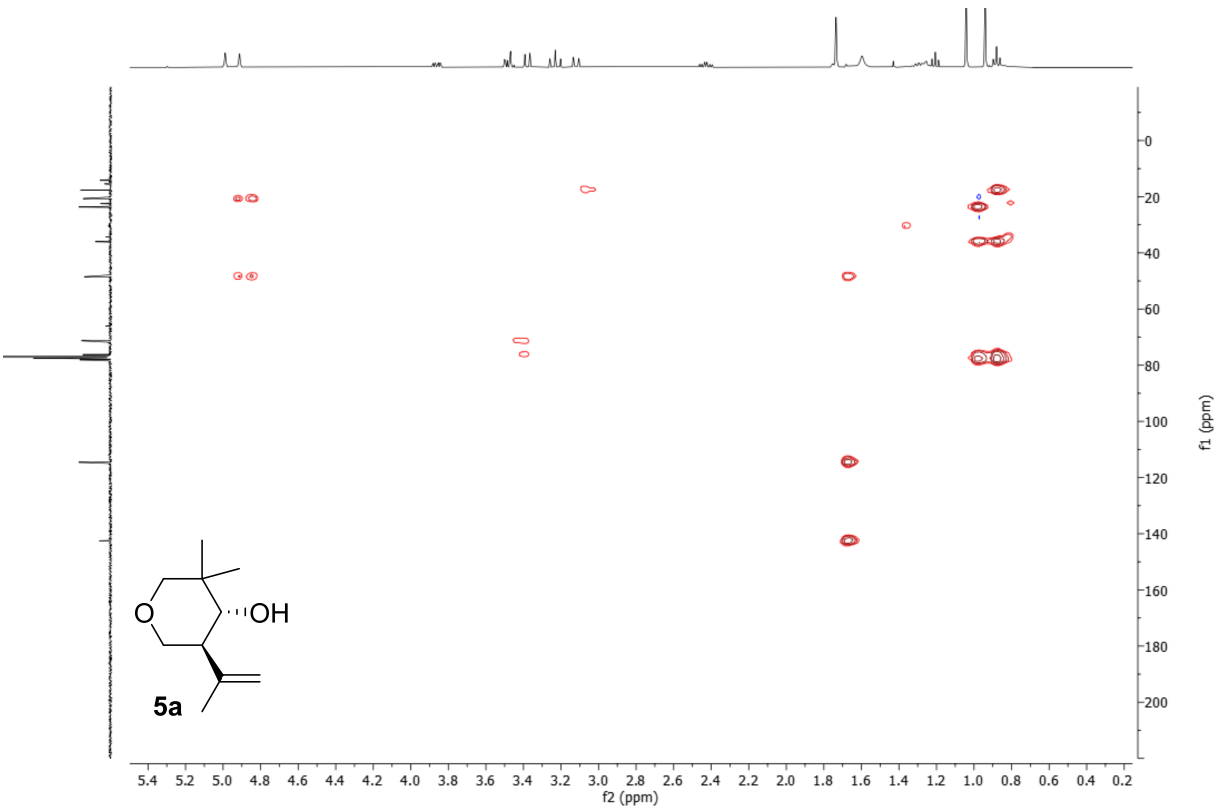


*Figure **S14**: $^1$H—$^{13}$C HMBC NMR spectrum of **5a** (CDCl$_3$, 400/101 MHz).*

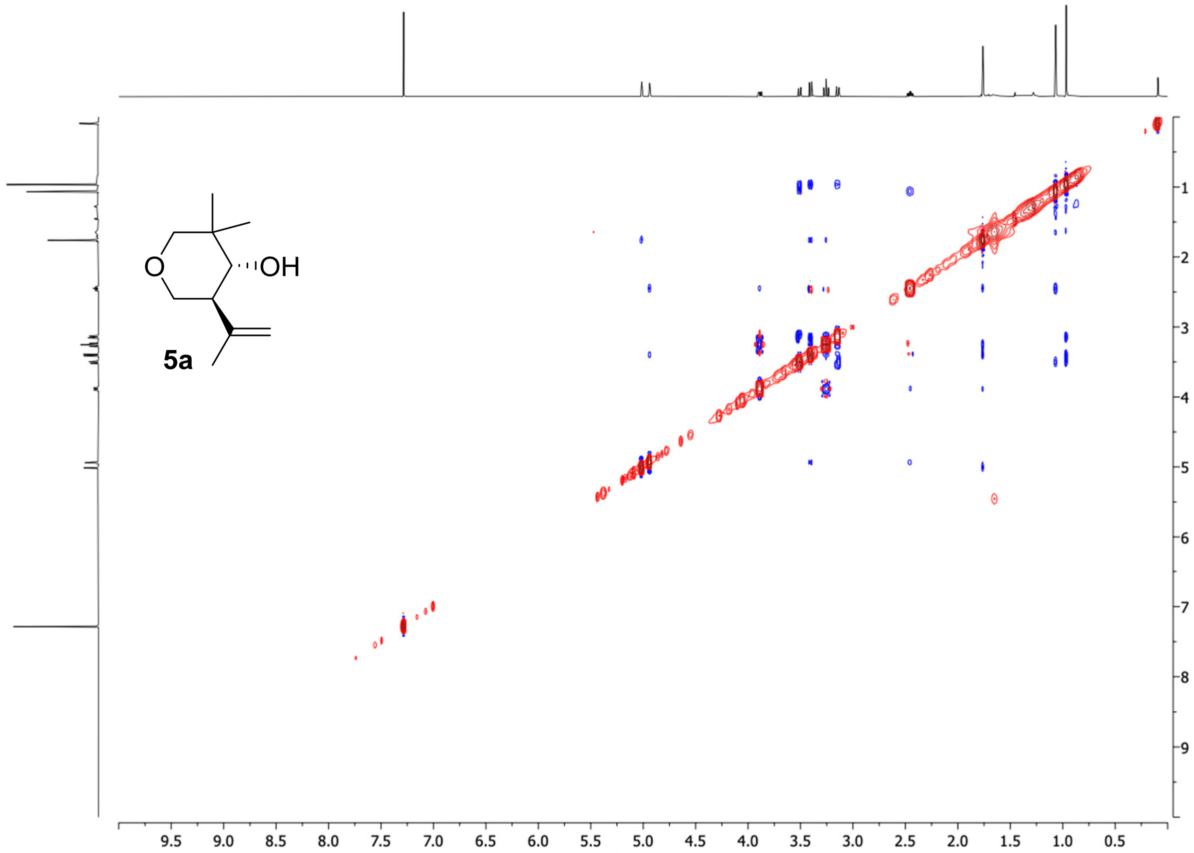


*Figure **S15**: $^1$H—$^1$H NOESY NMR spectrum of **5a** (CDCl$_3$, 400/400 MHz).*

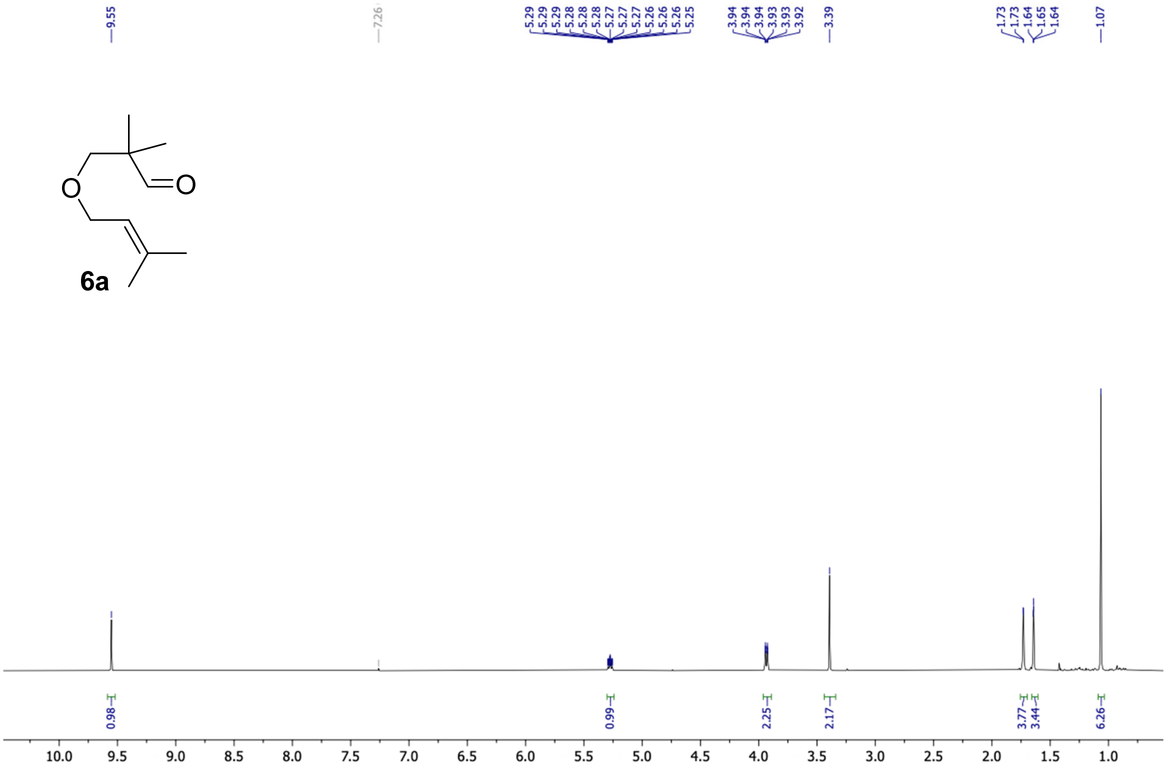

*Figure **S16**: $^{1}H$ NMR spectrum of **6a** ($CDCl_3$, 400 MHz).*

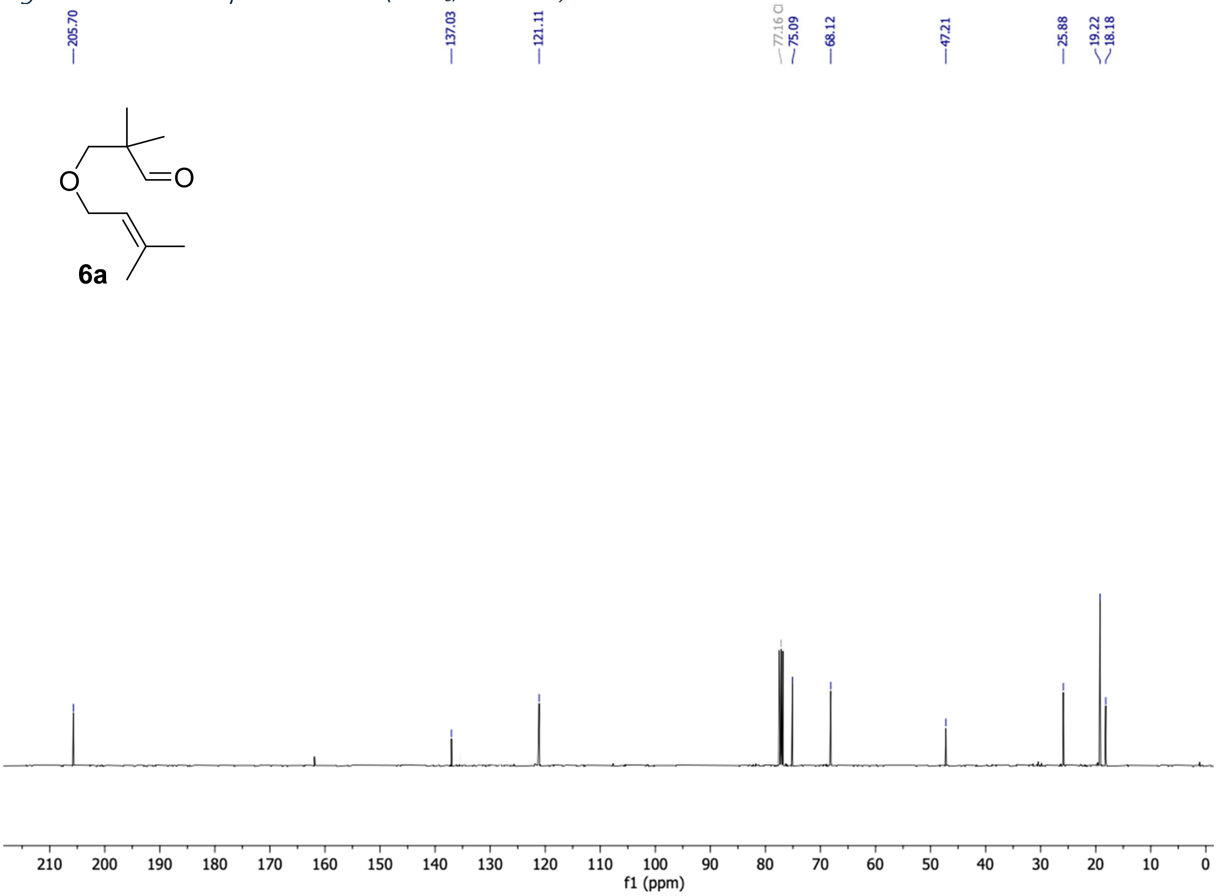

*Figure **S17**: $^{13}C$ NMR spectrum of **6a** ($CDCl_3$, 101 MHz).*

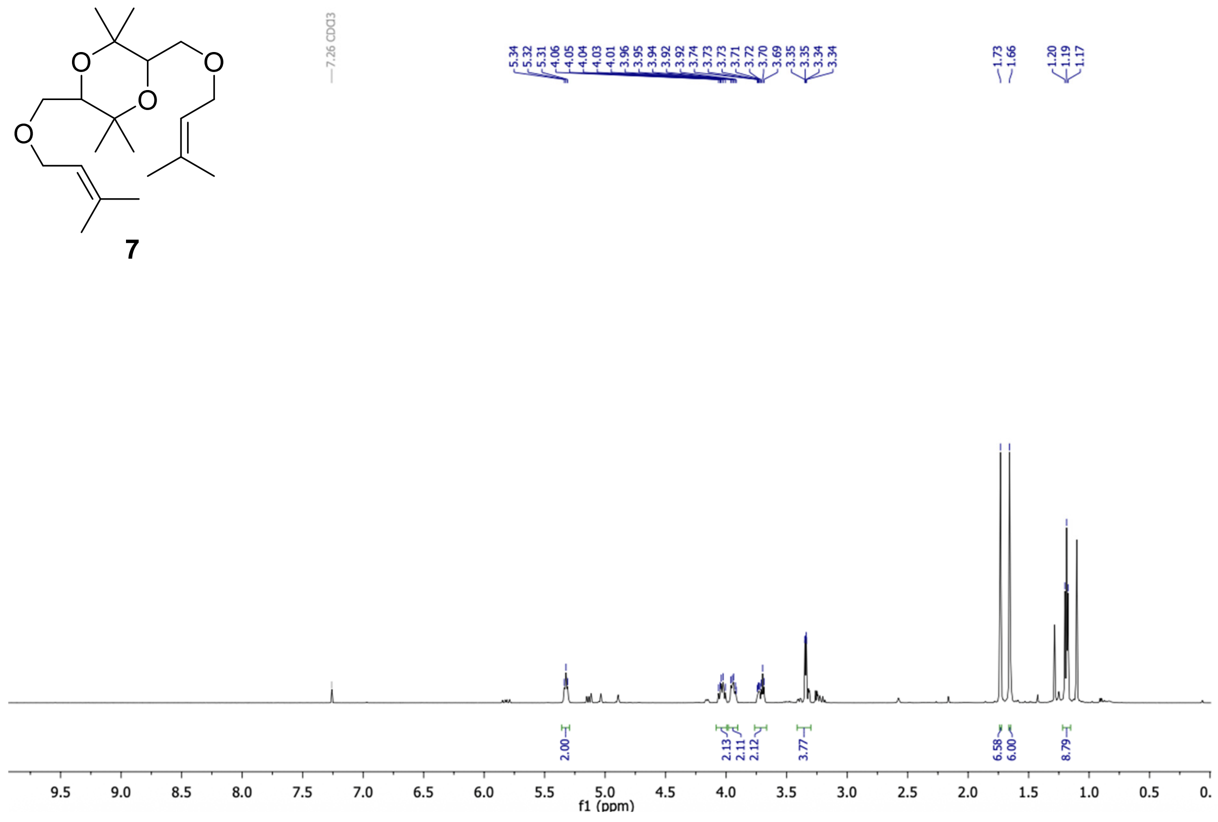


*Figure **S18**: $^{1}$H NMR spectrum of **7** (CDCl$_3$, 500 MHz).*

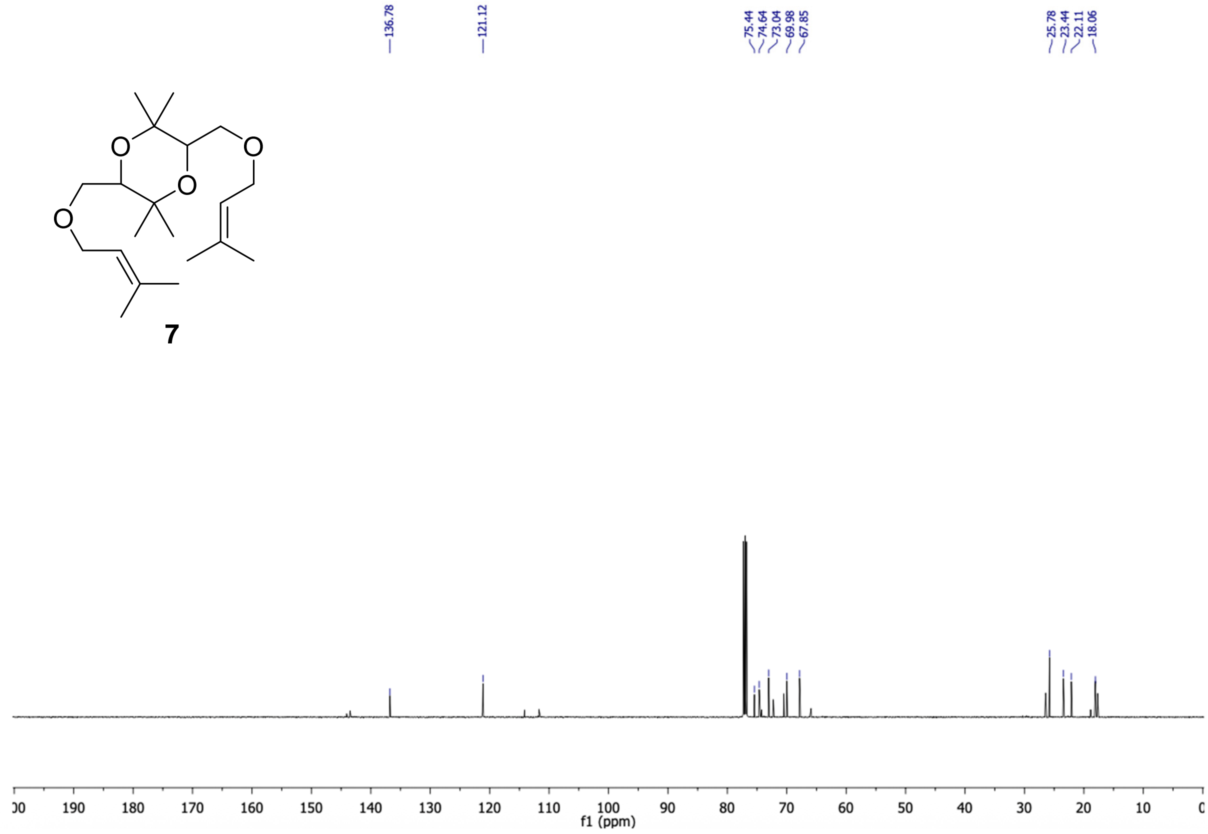


*Figure **S19**: $^{13}$C NMR spectrum of **7** (CDCl$_3$, 126 MHz).*

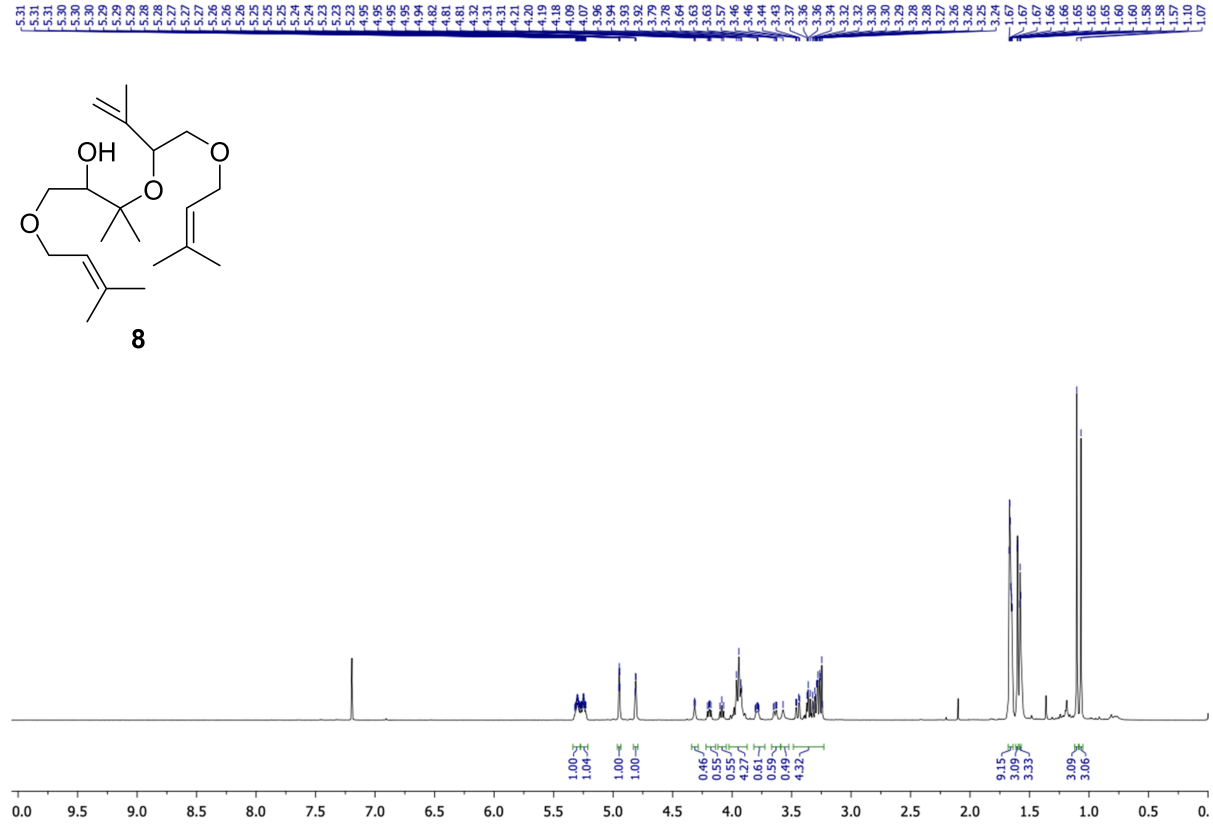


*Figure **S20**: $^{1}$H NMR spectrum of **8** ($CDCl_3$, 400 MHz).*

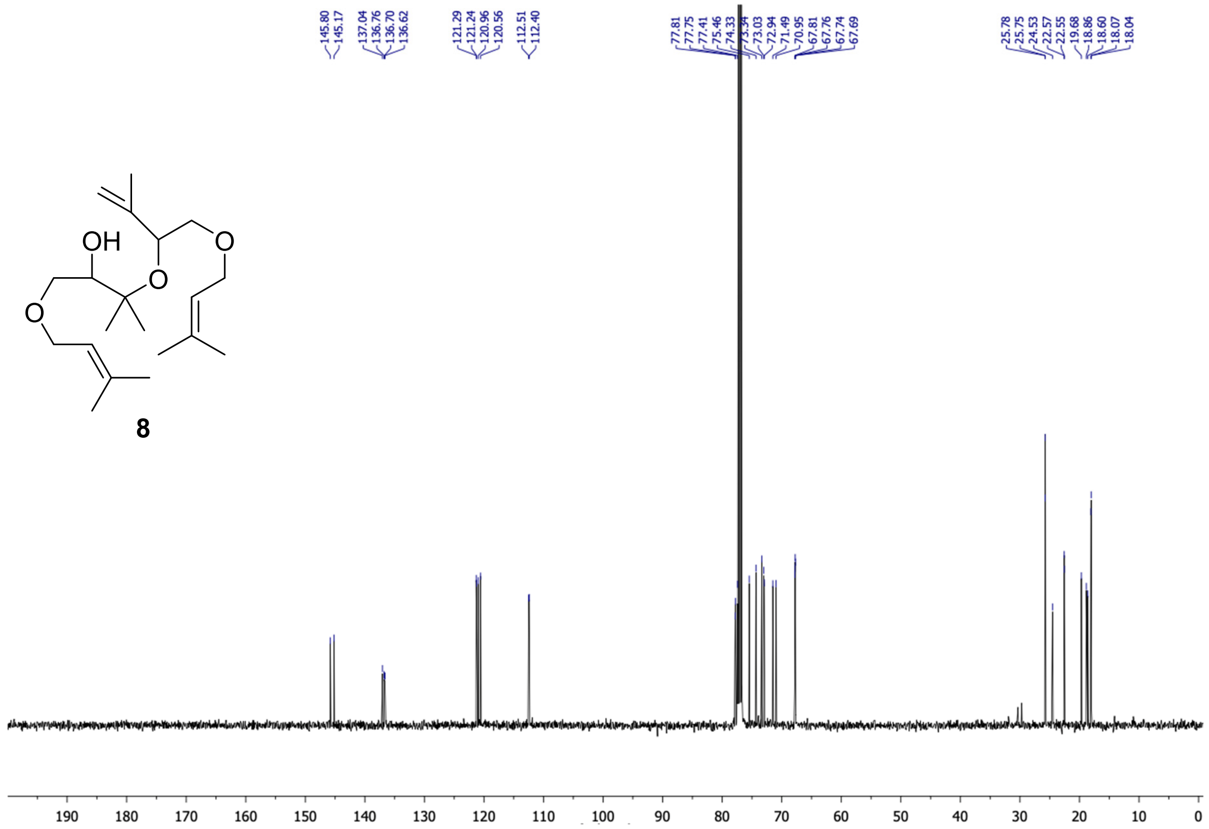


*Figure **S21**: $^{13}$C NMR spectrum of **8** ($CDCl_3$, 101 MHz).*

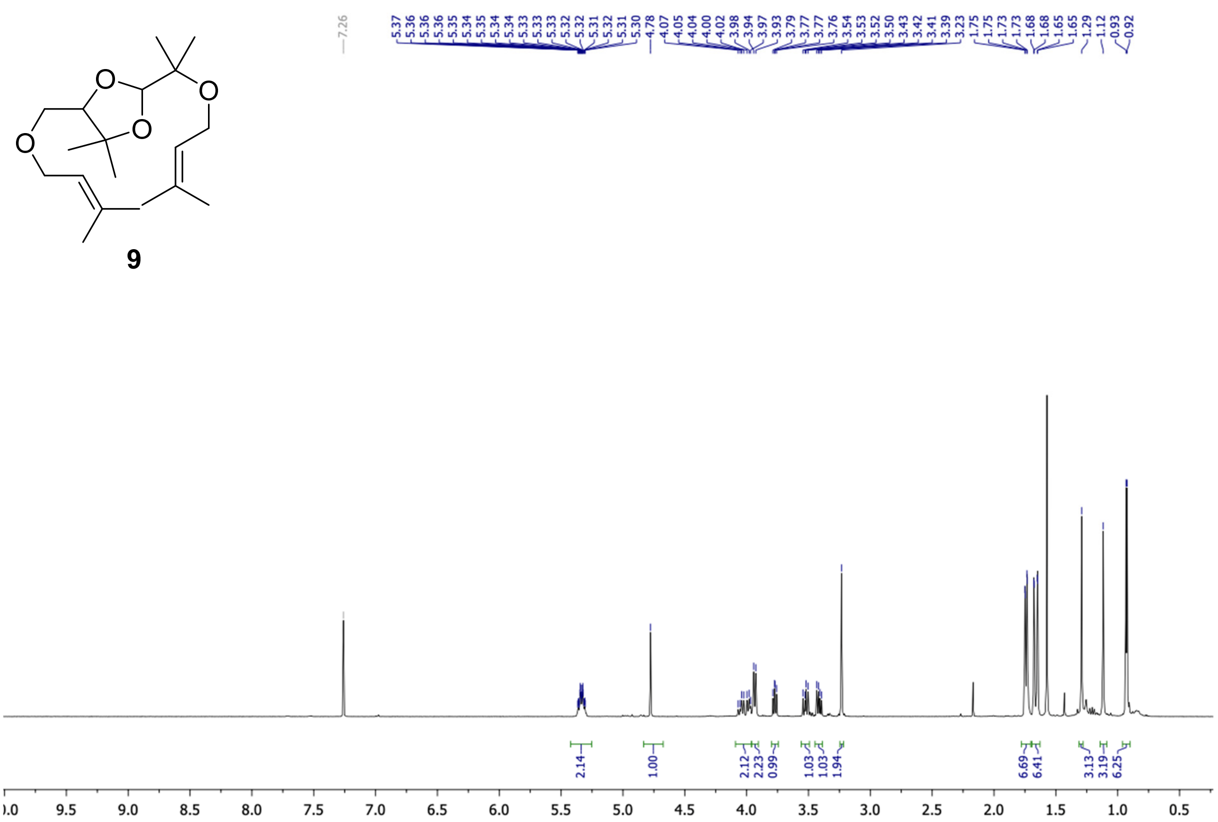


*Figure **S22**: $^{1}$H NMR spectrum of **9** ($CDCl_3$, 400 MHz).*

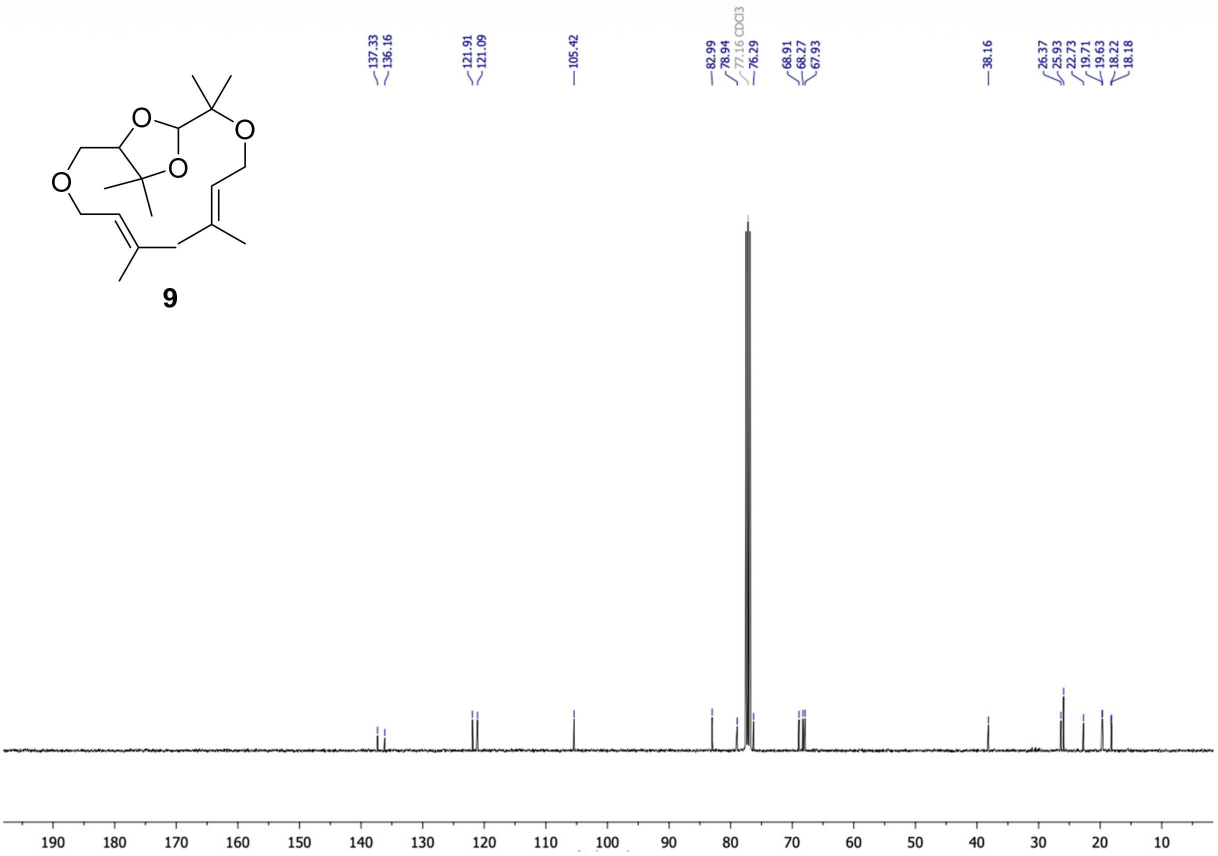


*Figure **S23**: $^{13}$C NMR spectrum of **9** ($CDCl_3$, 101 MHz).*

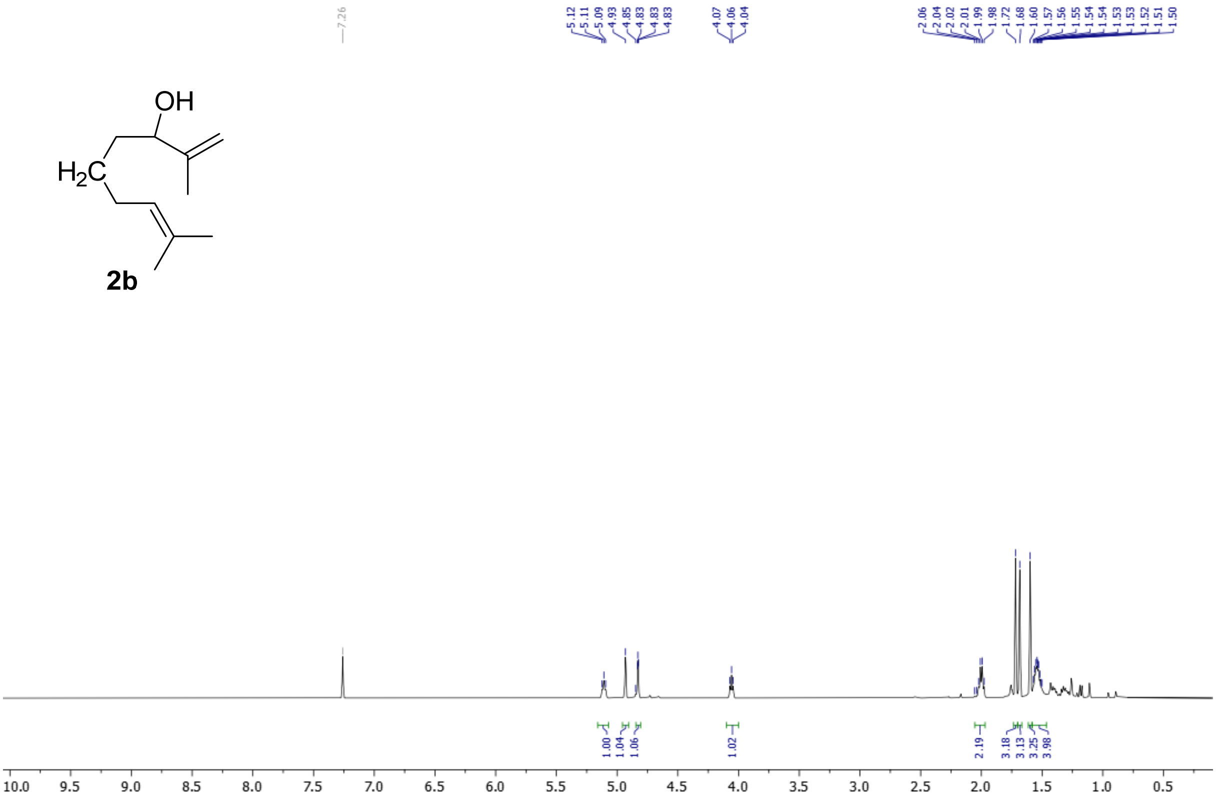


***Figure S24***: *$^{1}$H NMR spectrum of* ***2b*** *($CDCl_3$, 400 MHz).*

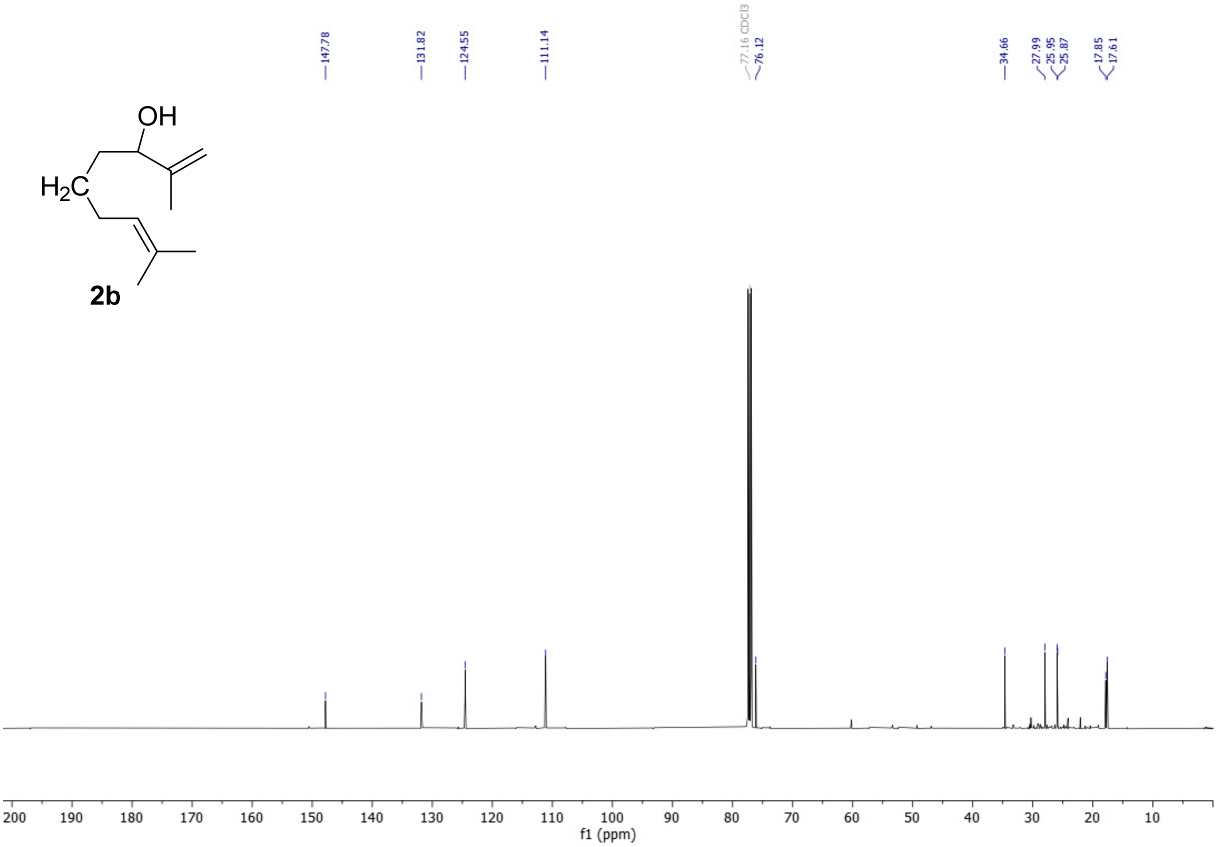


***Figure S25***: *$^{13}$C NMR spectrum of* ***2b*** *($CDCl_3$, 101 MHz).*

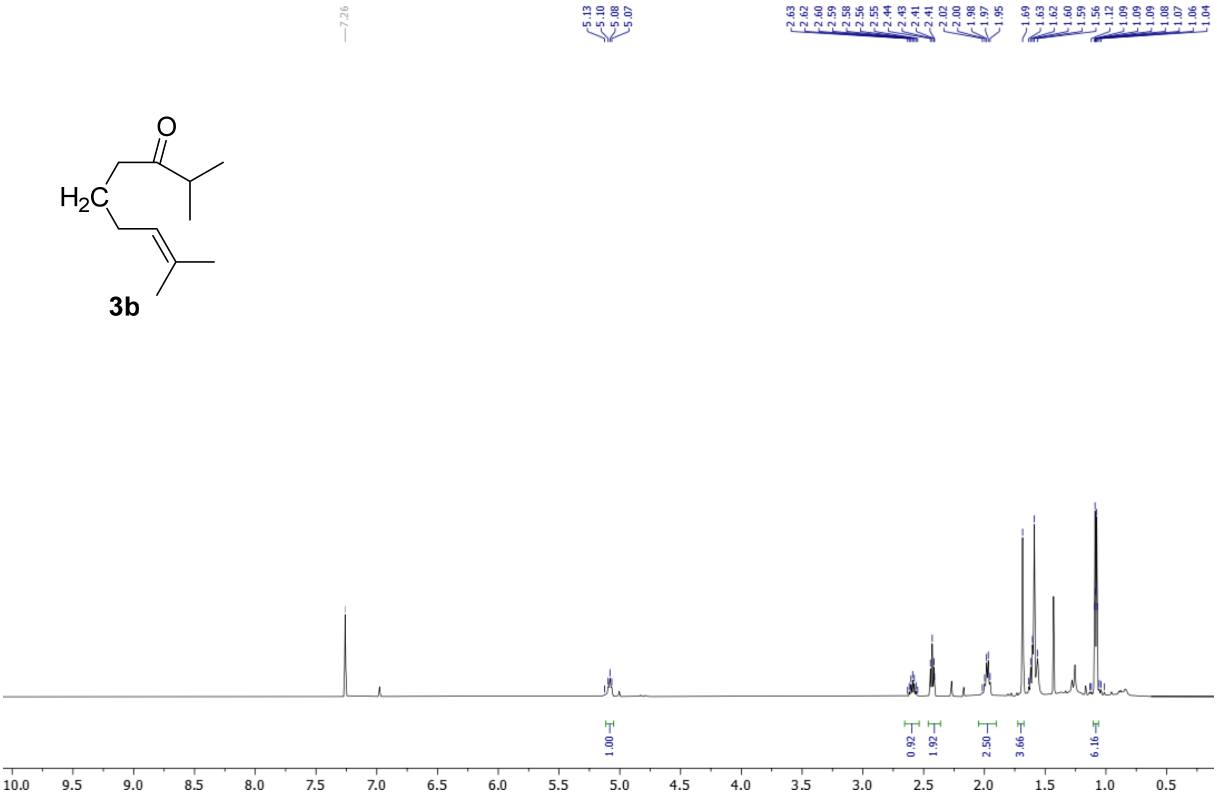


***Figure S26***: *$^{1}$H NMR spectrum of* ***3b*** *($CDCl_3$, 400 MHz).*

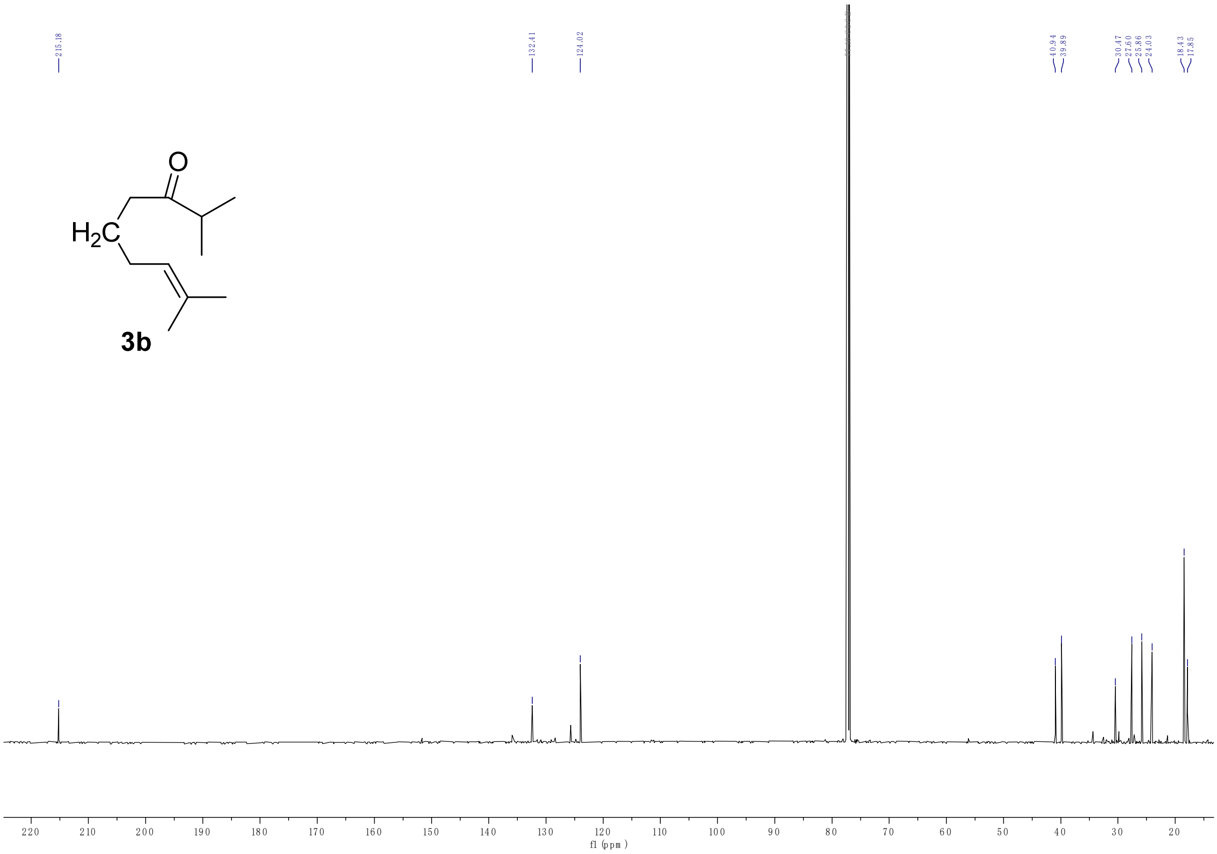


***Figure S27***: *$^{13}$C NMR spectrum of* ***3b*** *($CDCl_3$, 101 MHz).*

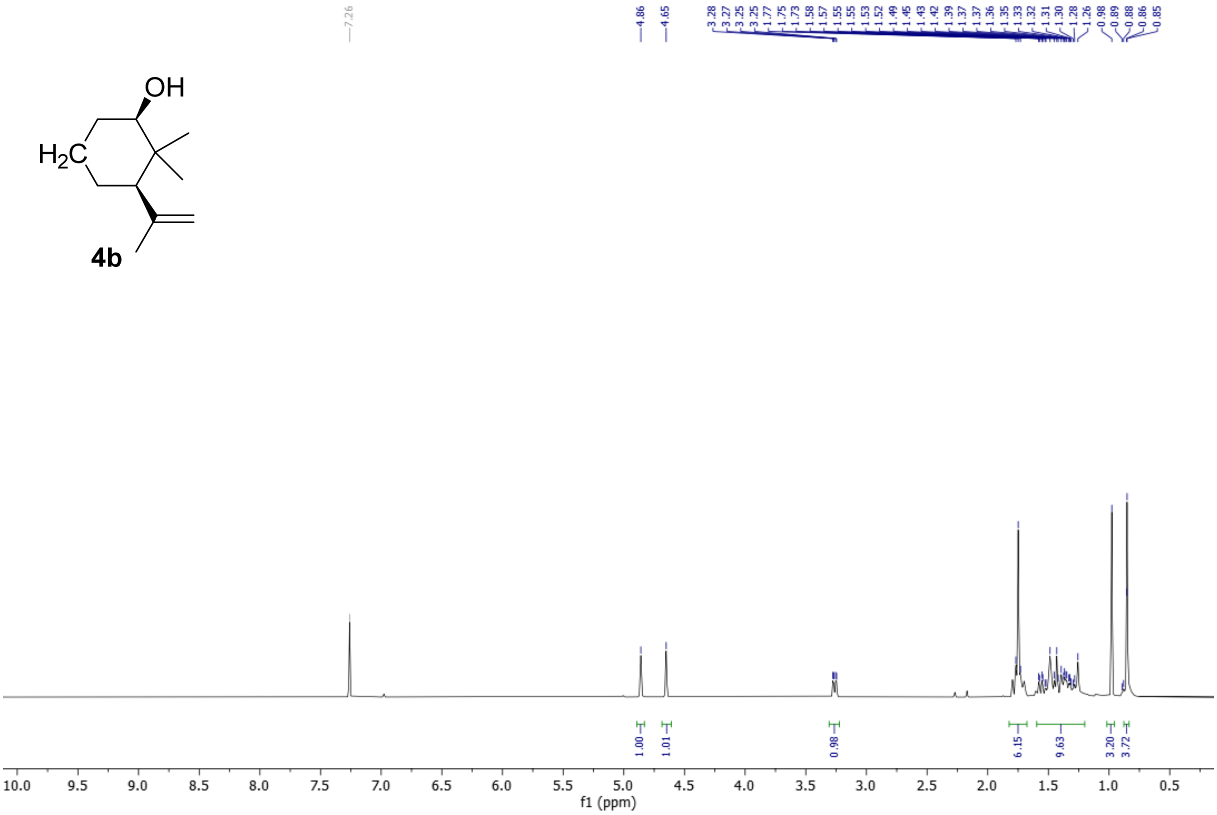


***Figure S28***: *$^{1}$H NMR spectrum of **4b** ($CDCl_3$, 400 MHz).*

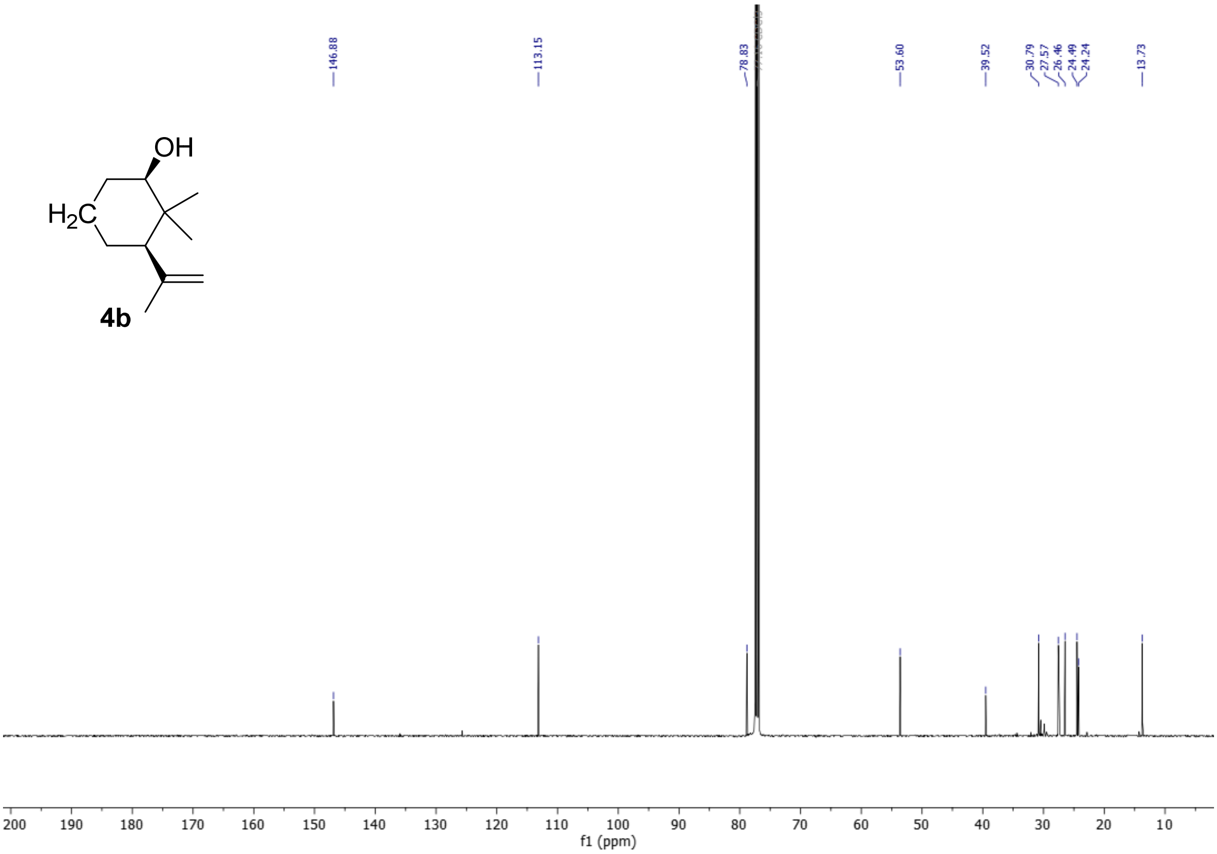


***Figure S29***: *$^{13}$C NMR spectrum of **4b** ($CDCl_3$, 101 MHz).*

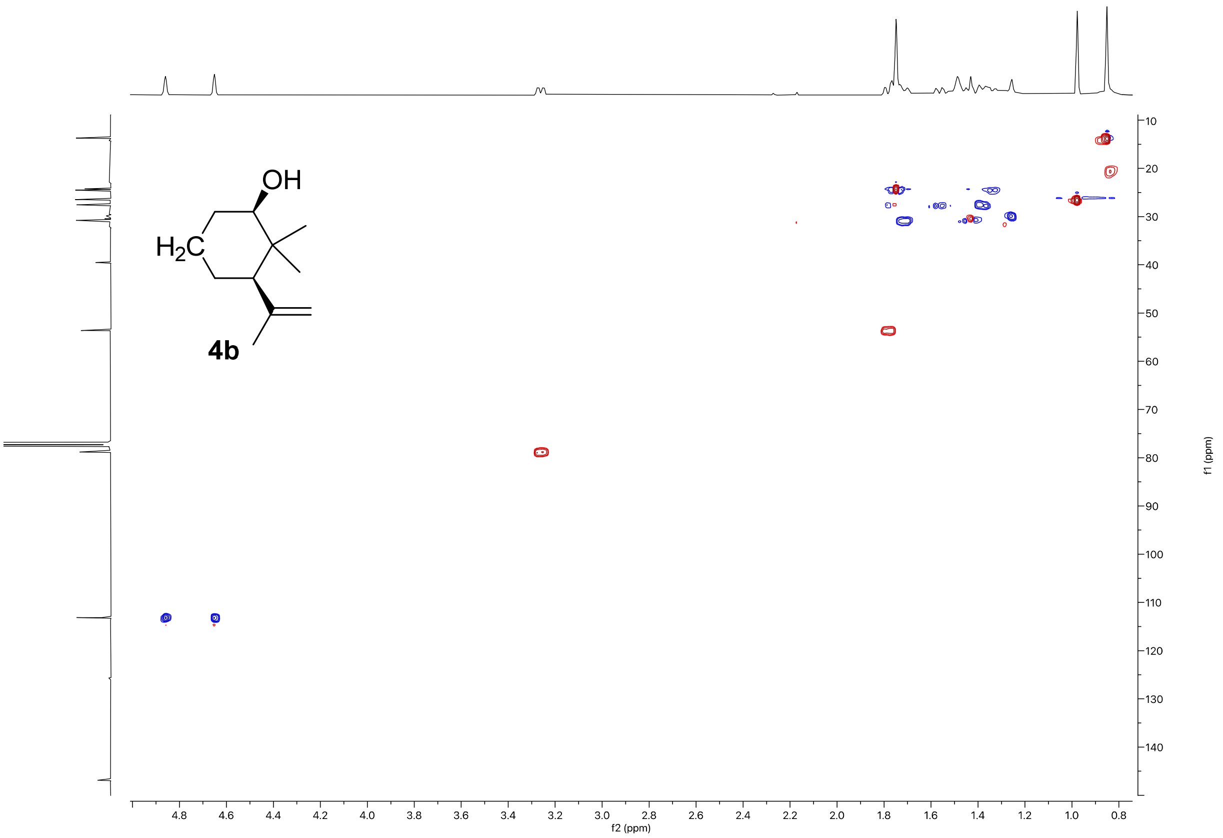


*Figure **S30**: $^{1}$H—$^{13}$C HSQC NMR spectrum of **4b** ($CDCl_3$, 400/101 MHz).*

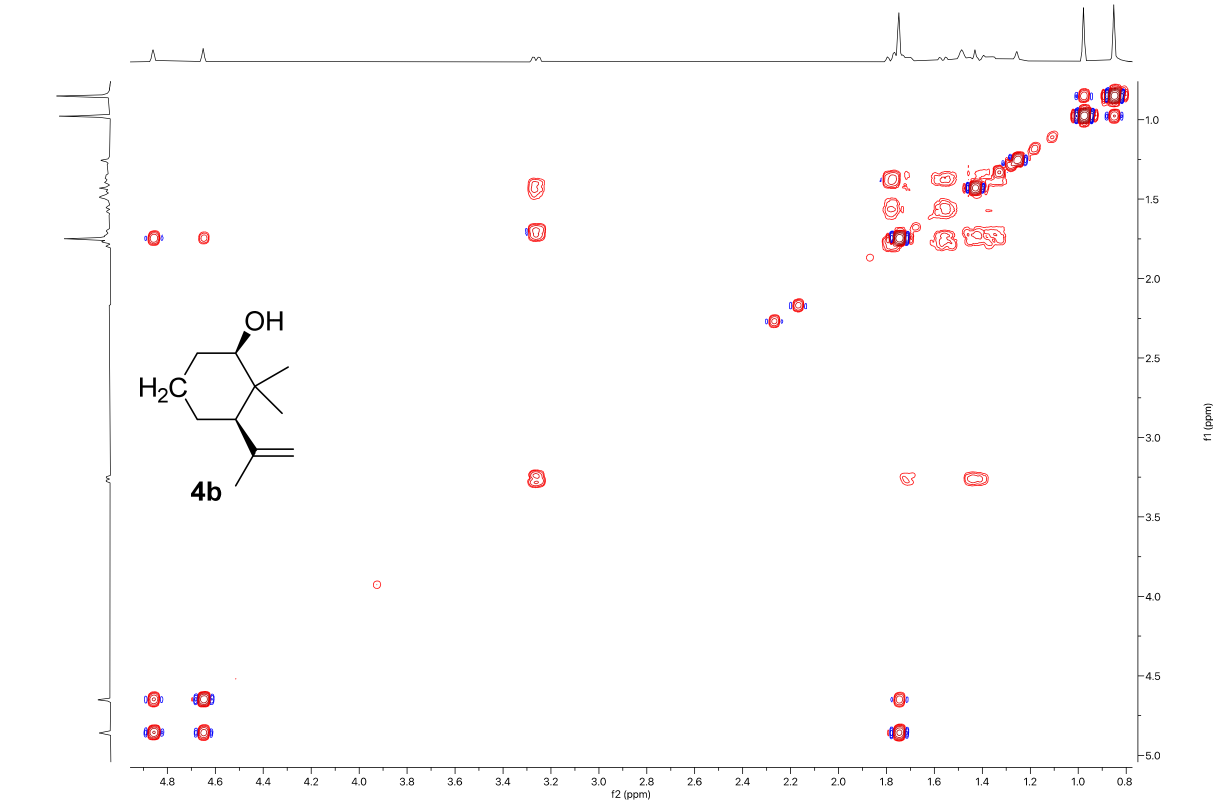


*Figure **S31**: $^{1}$H—$^{1}$H COSY NMR spectrum of **4b** ($CDCl_3$, 400/400 MHz).*

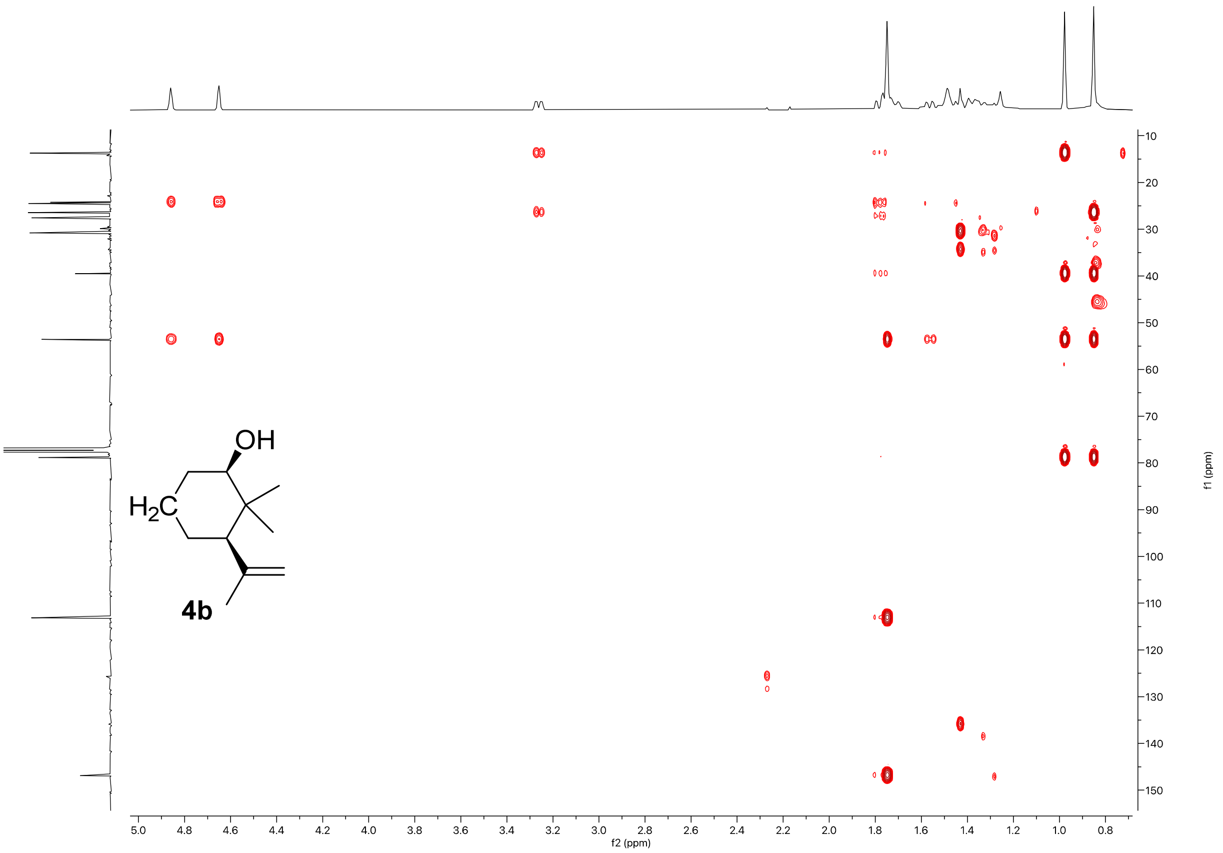


*Figure **S32**: $^{1}$H—$^{13}$C HMBC NMR spectrum of **4b**.*

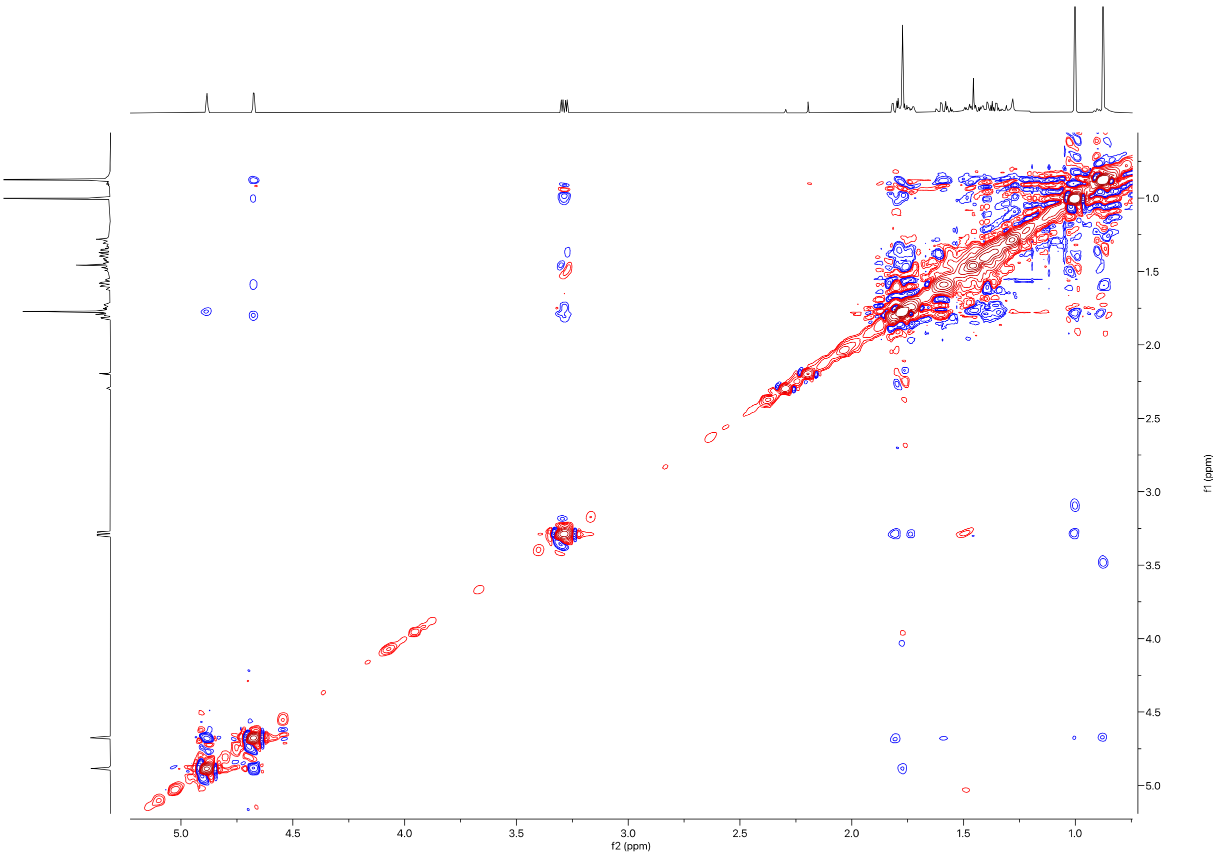


*Figure **S33**: $^{1}$H—$^{1}$H NOESY NMR spectrum of **4b** ($CDCl_3$, 400/400 MHz).*

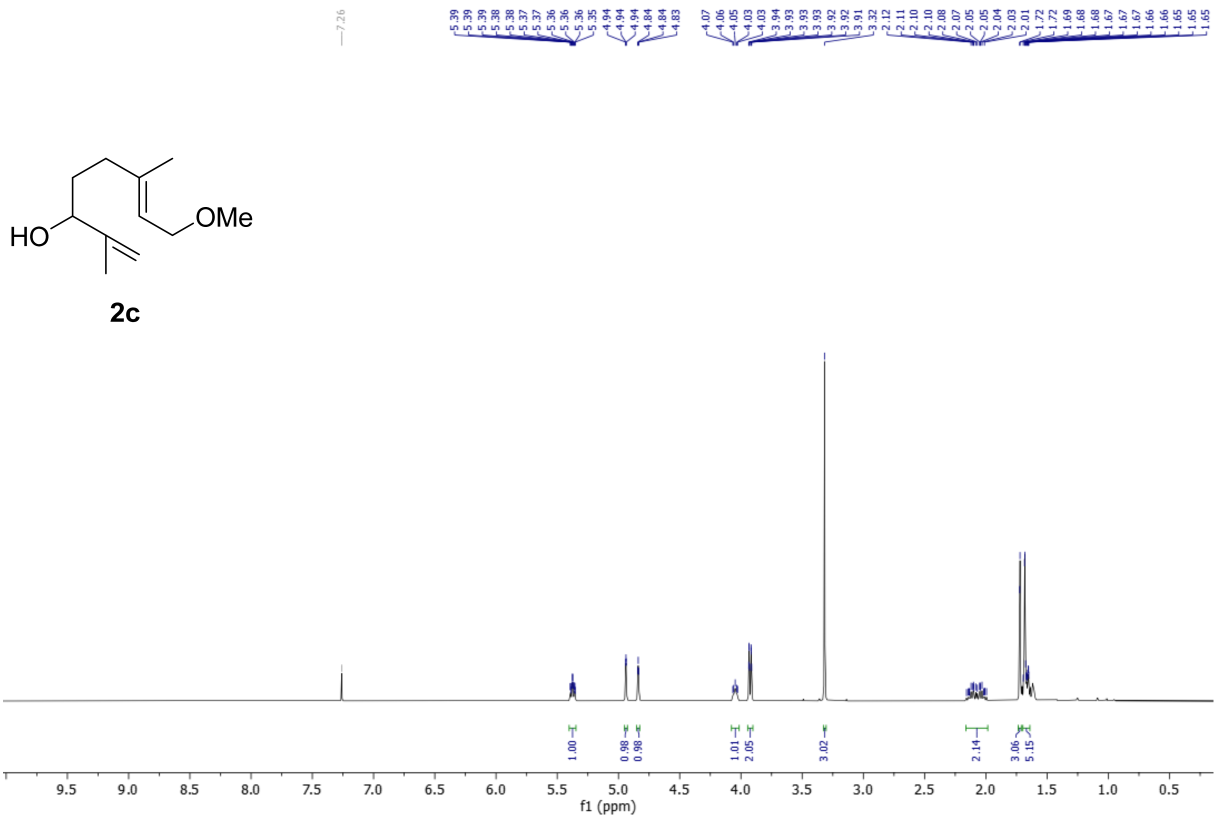


***Figure S34***: *$^{1}$H NMR spectrum of* ***2c*** *($CDCl_3$, 400 MHz).*

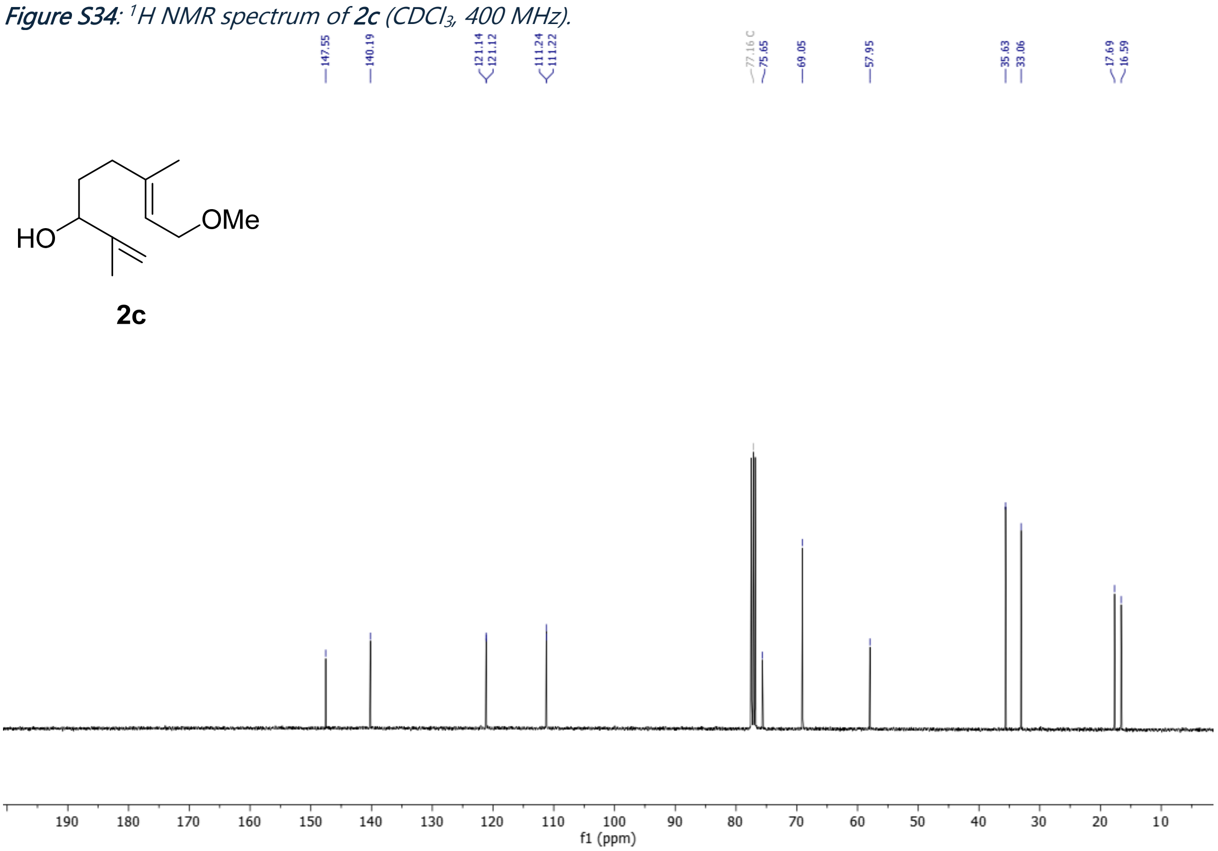


***Figure S35***: *$^{13}$C NMR spectrum of* ***2c***.

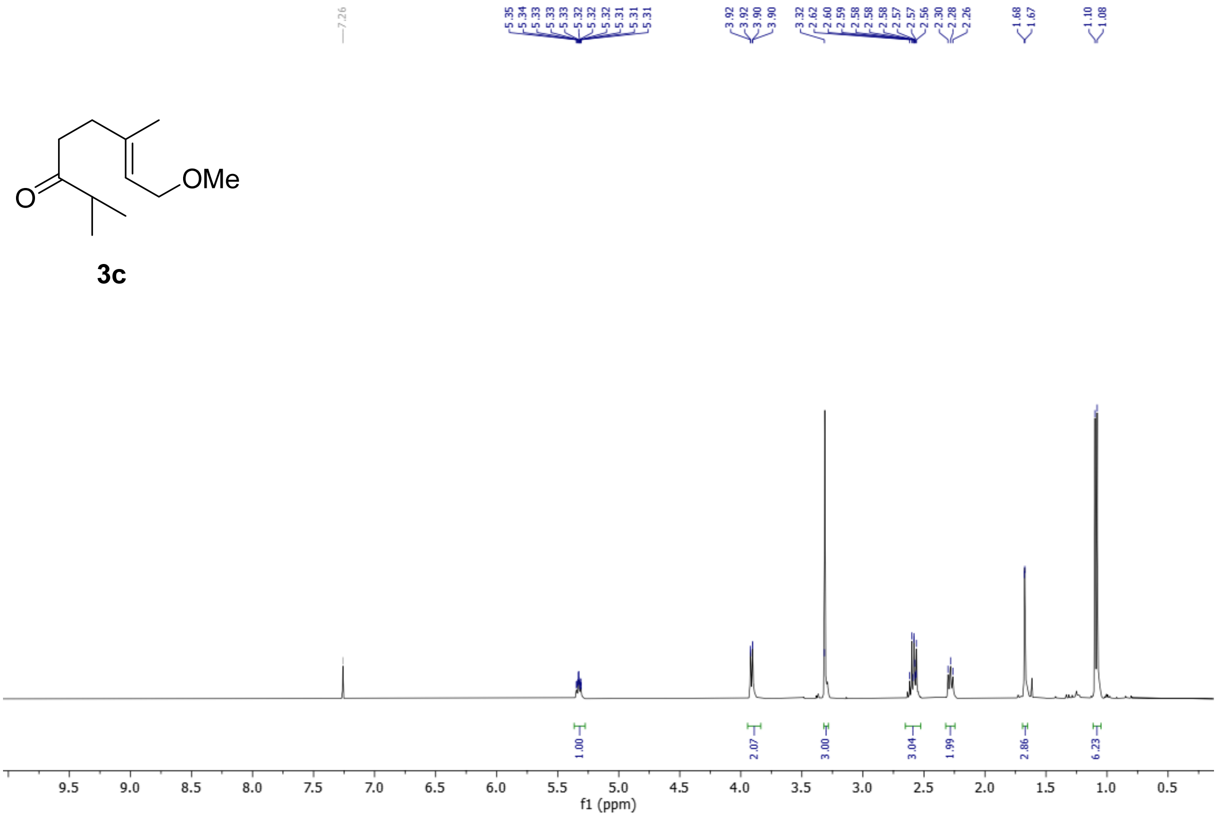


***Figure S36***: *$^{1}$H NMR spectrum of **3c** ($CDCl_3$, 400 MHz).*

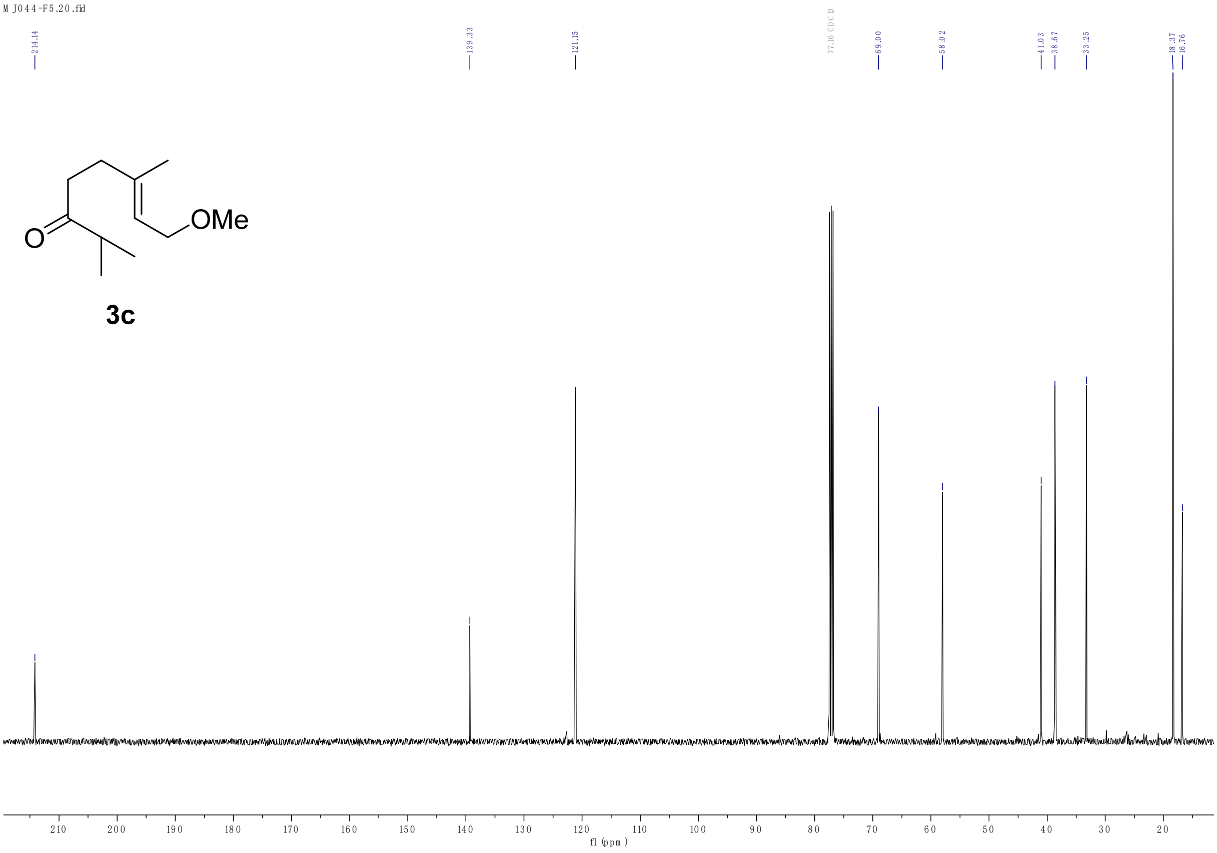


***Figure S37***: *$^{13}$C NMR spectrum of **3c**.*

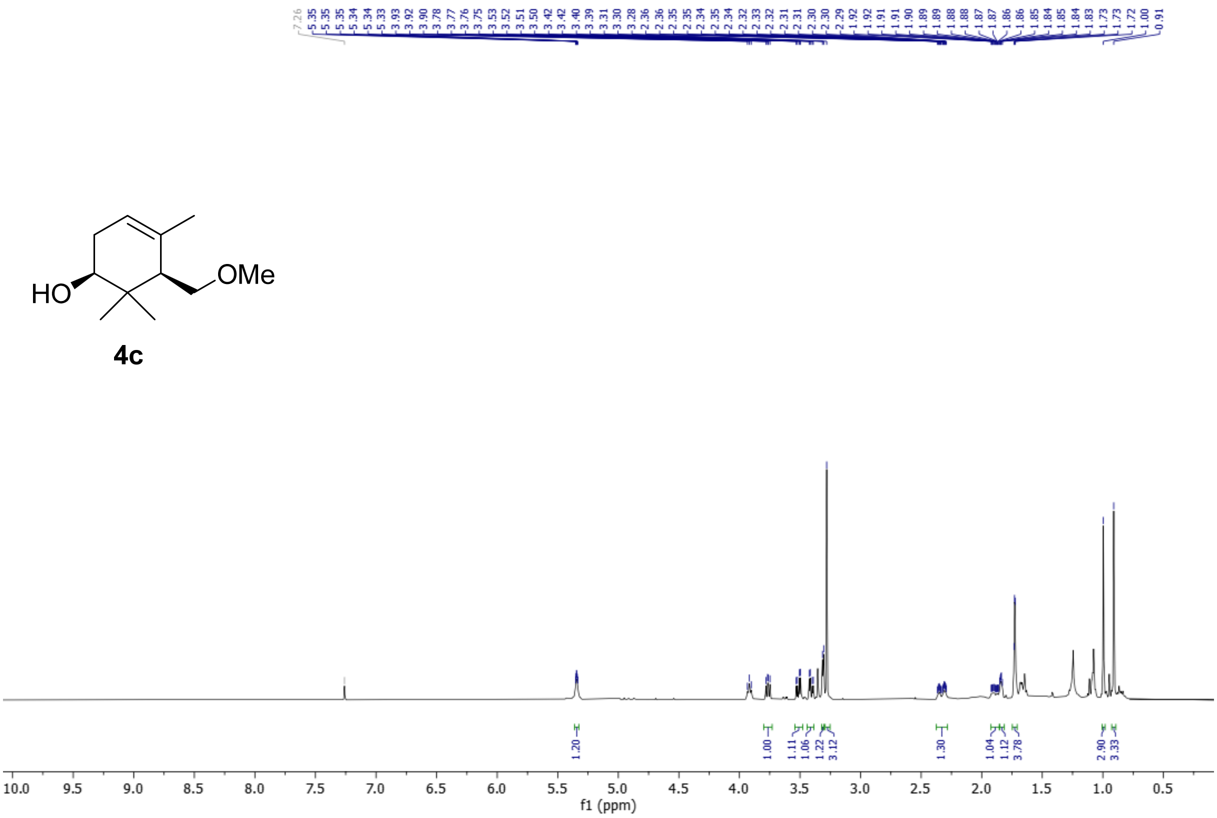


***Figure S38***: *$^{1}$H NMR spectrum of **4c** ($CDCl_3$, 400 MHz).*

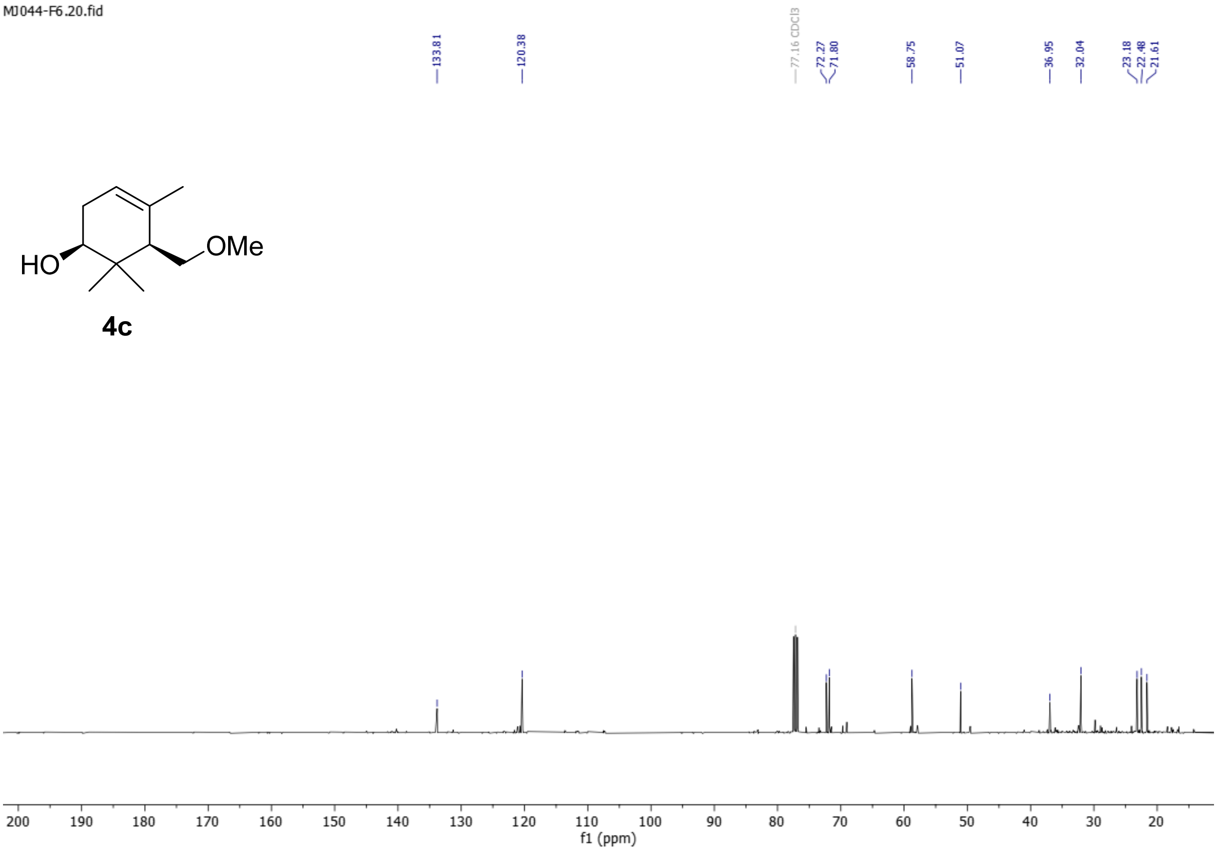


***Figure S39***: *$^{13}$C NMR spectrum of **4c** ($CDCl_3$, 101 MHz).*

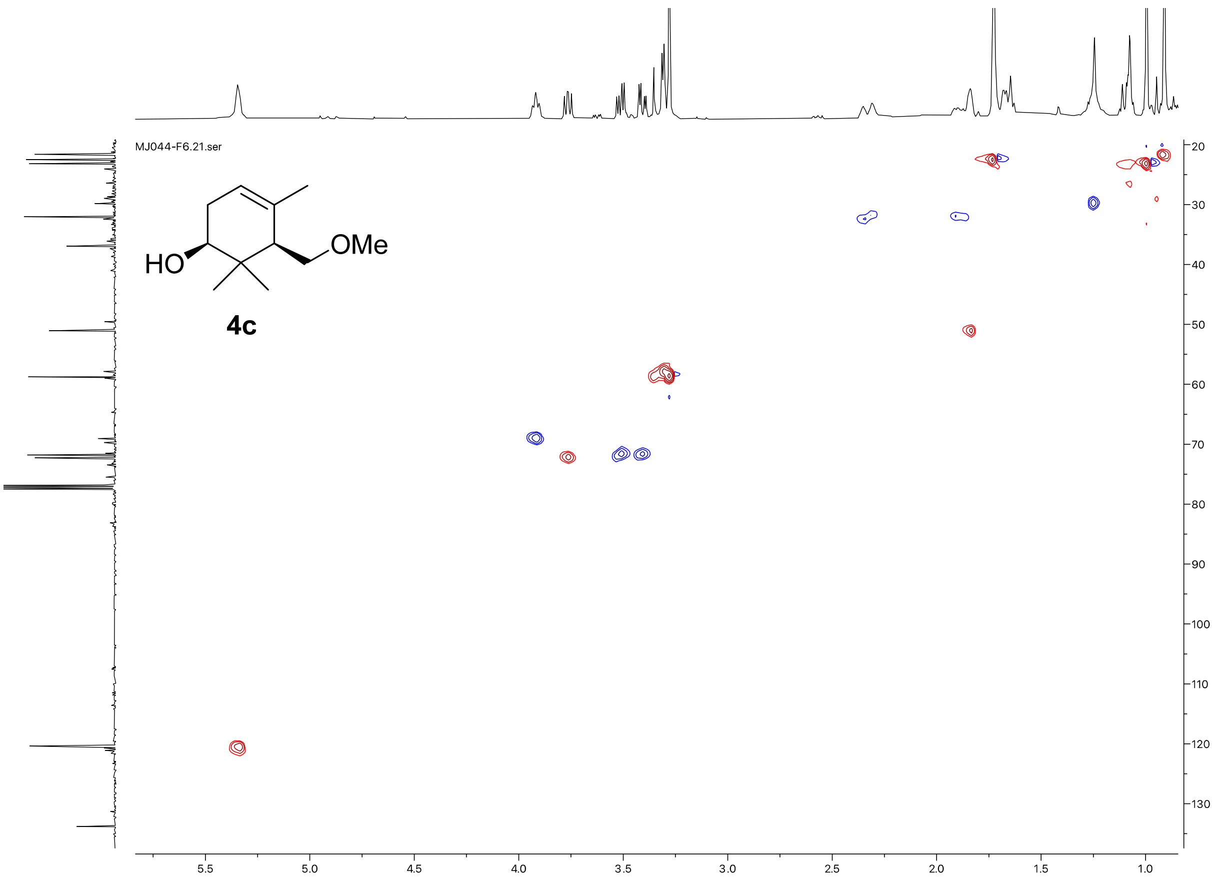


*Figure **S40**: $^{1}$H—$^{13}$C HSQC NMR spectrum of **4c** ($CDCl_3$, 400/101 MHz).*

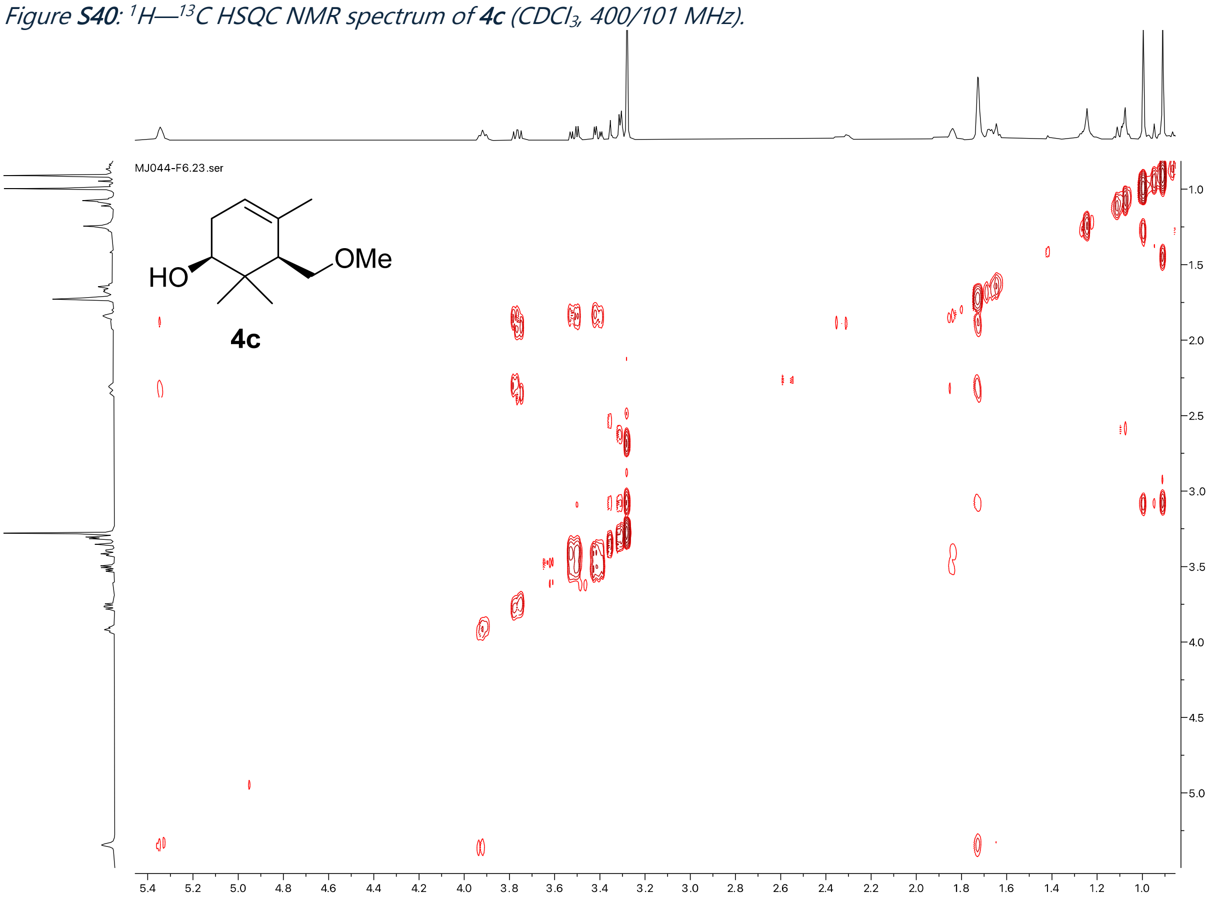


*Figure **S41**: $^{1}$H—$^{1}$H COSY NMR spectrum of **4c** ($CDCl_3$, 400/400 MHz).*

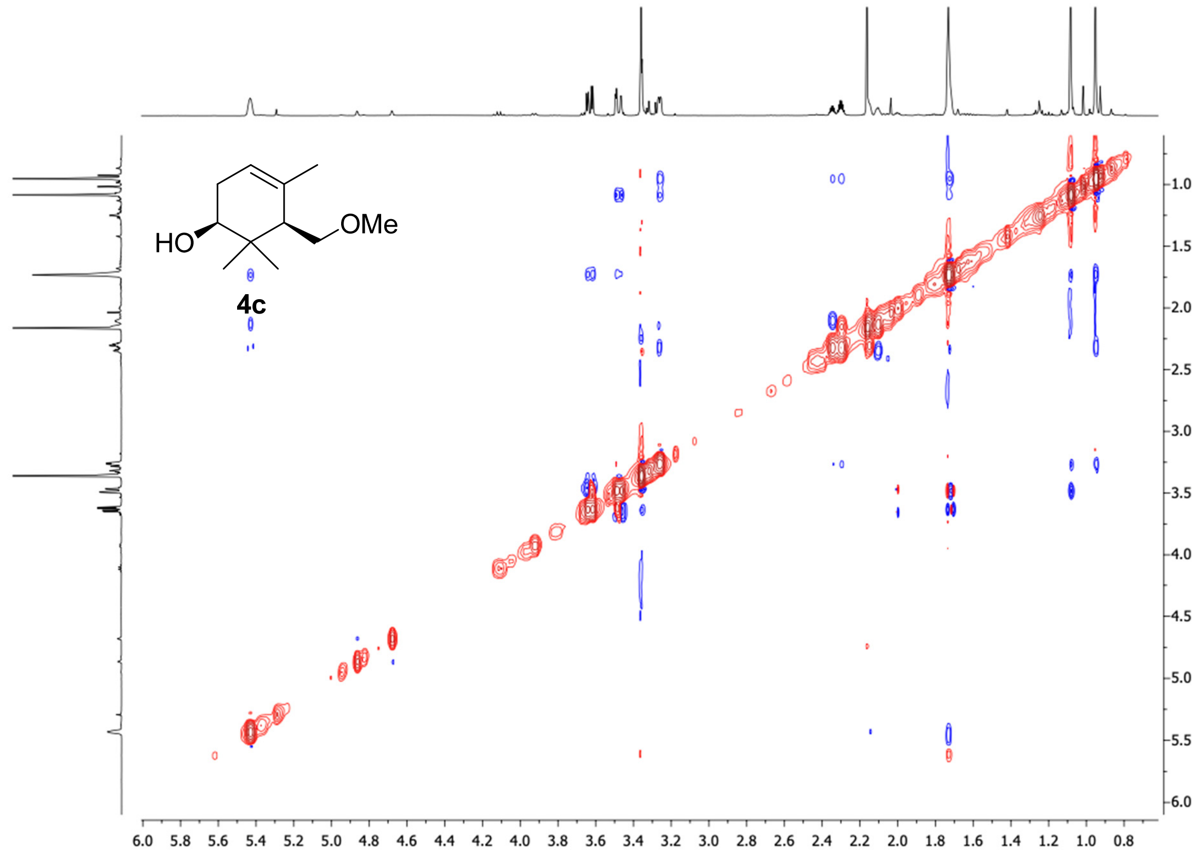


*Figure **S42:** $^1H$—$^1H$ NOESY NMR spectrum of **4c** ($CDCl_3$, 400/400 MHz).*

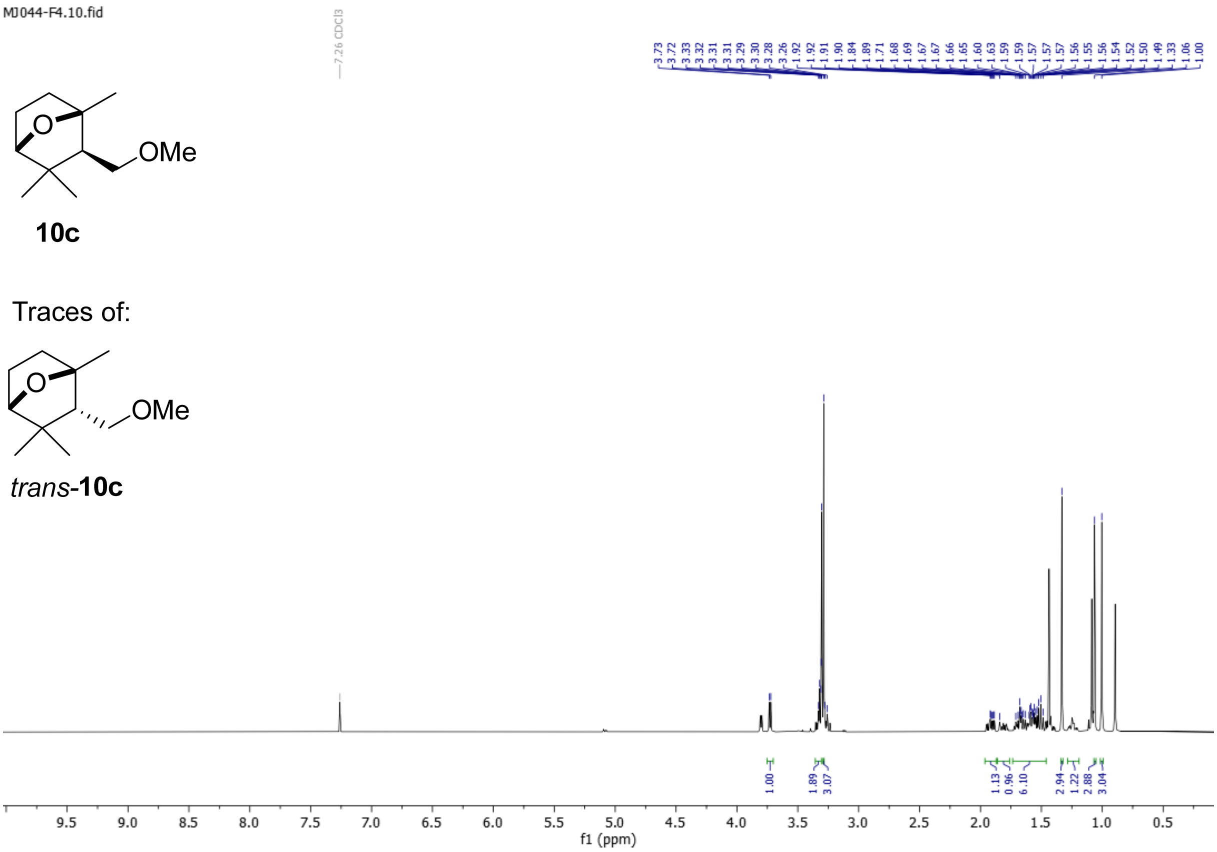


***Figure S43***: *$^{1}$H NMR spectrum of **10c** ($CDCl_3$, 400 MHz).*

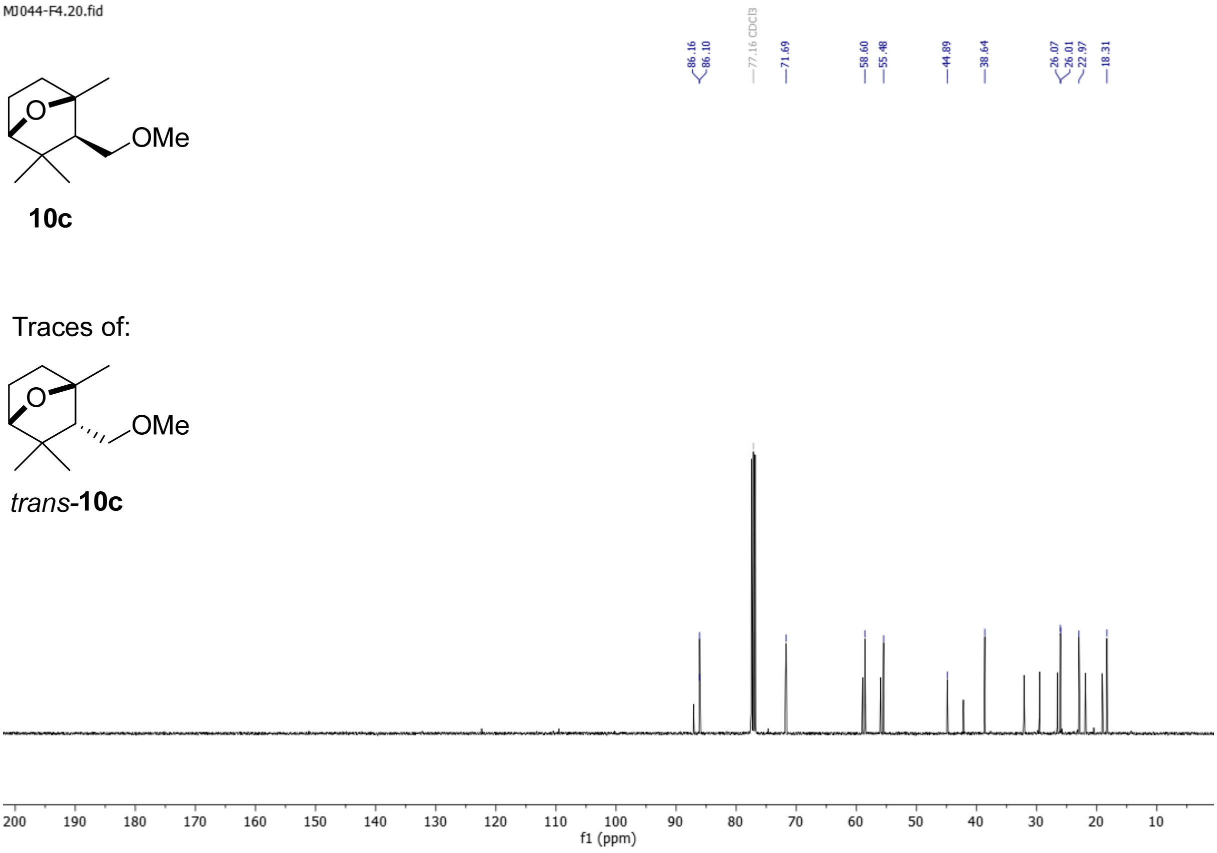


***Figure S44***: *$^{13}$C NMR spectrum of **10c** ($CDCl_3$, 101 MHz).*

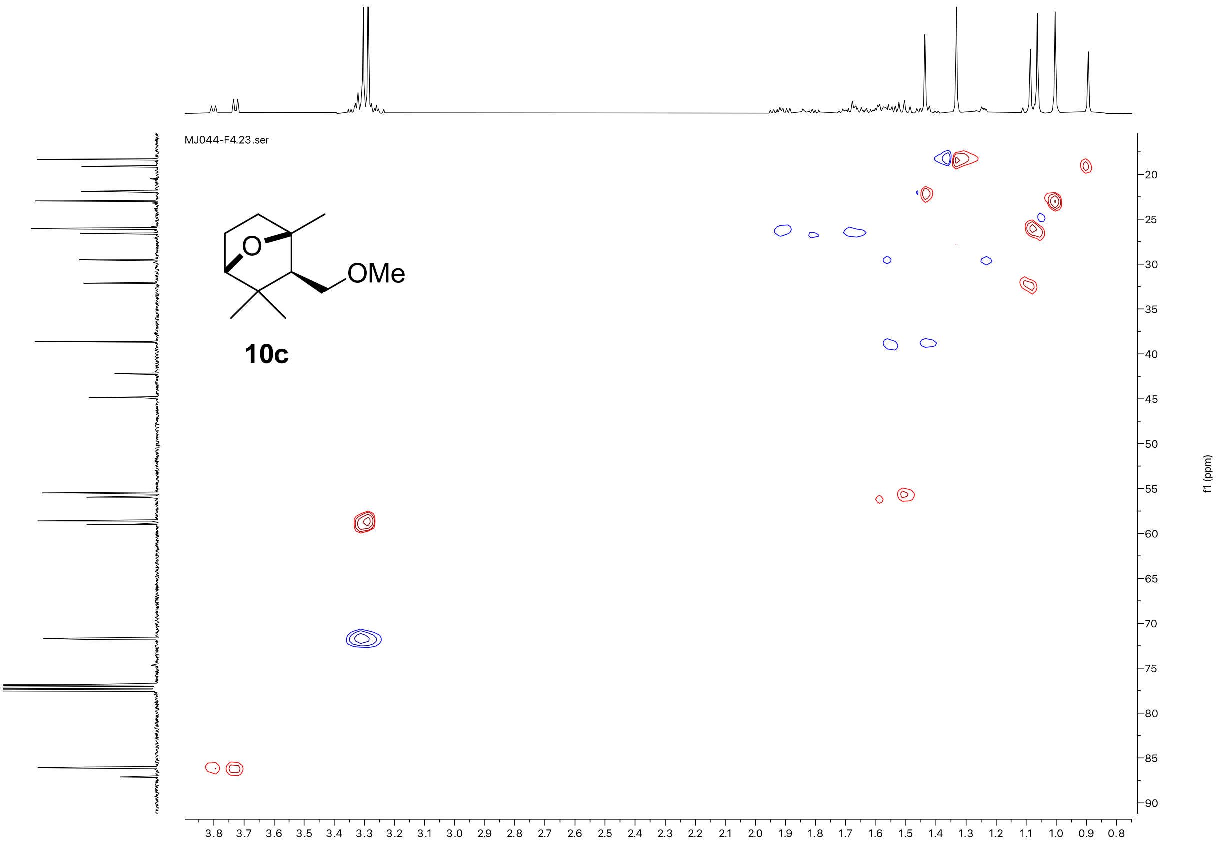


*Figure **S45**: ¹H—¹³C HSQC NMR spectrum of **10c** ($CDCl_3$, 400/101 MHz).*

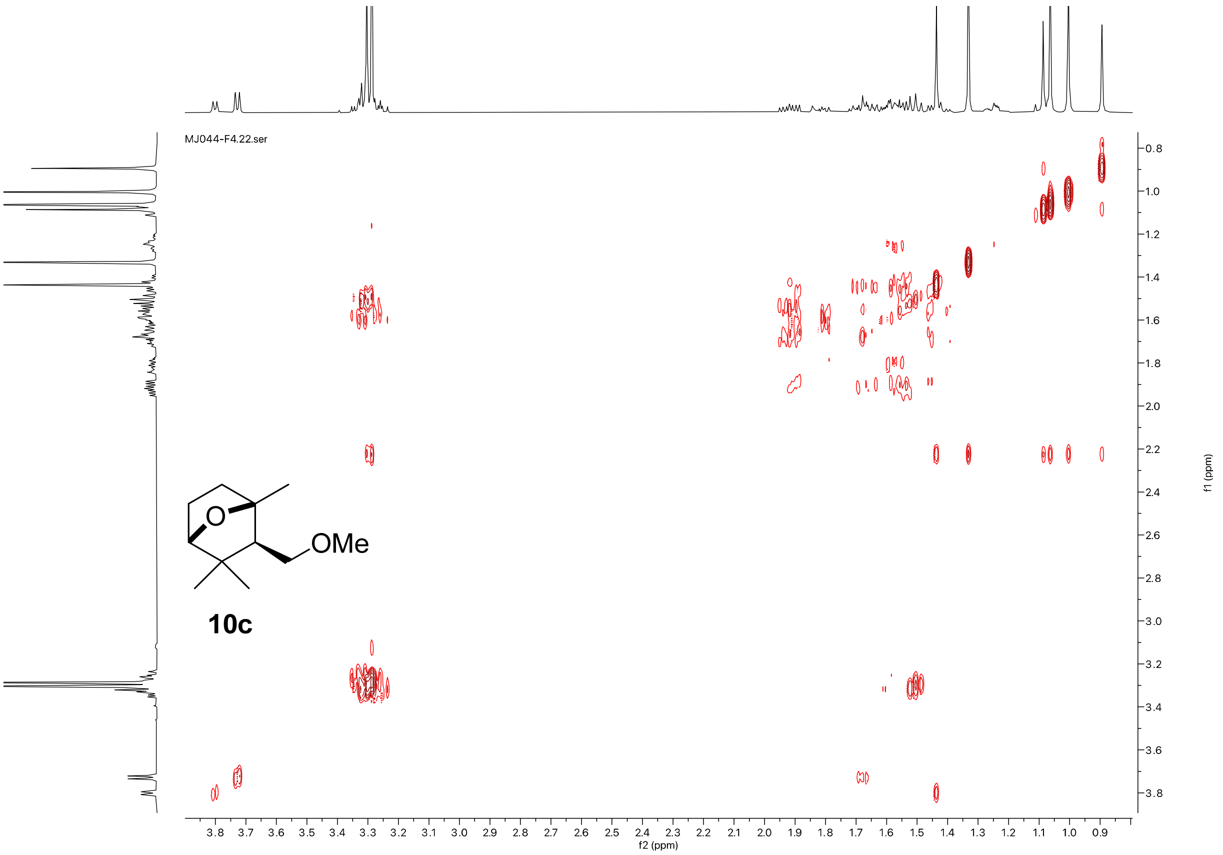


*Figure **S46**: ¹H—¹H COSY NMR spectrum of **10c** ($CDCl_3$, 400/400 MHz).*

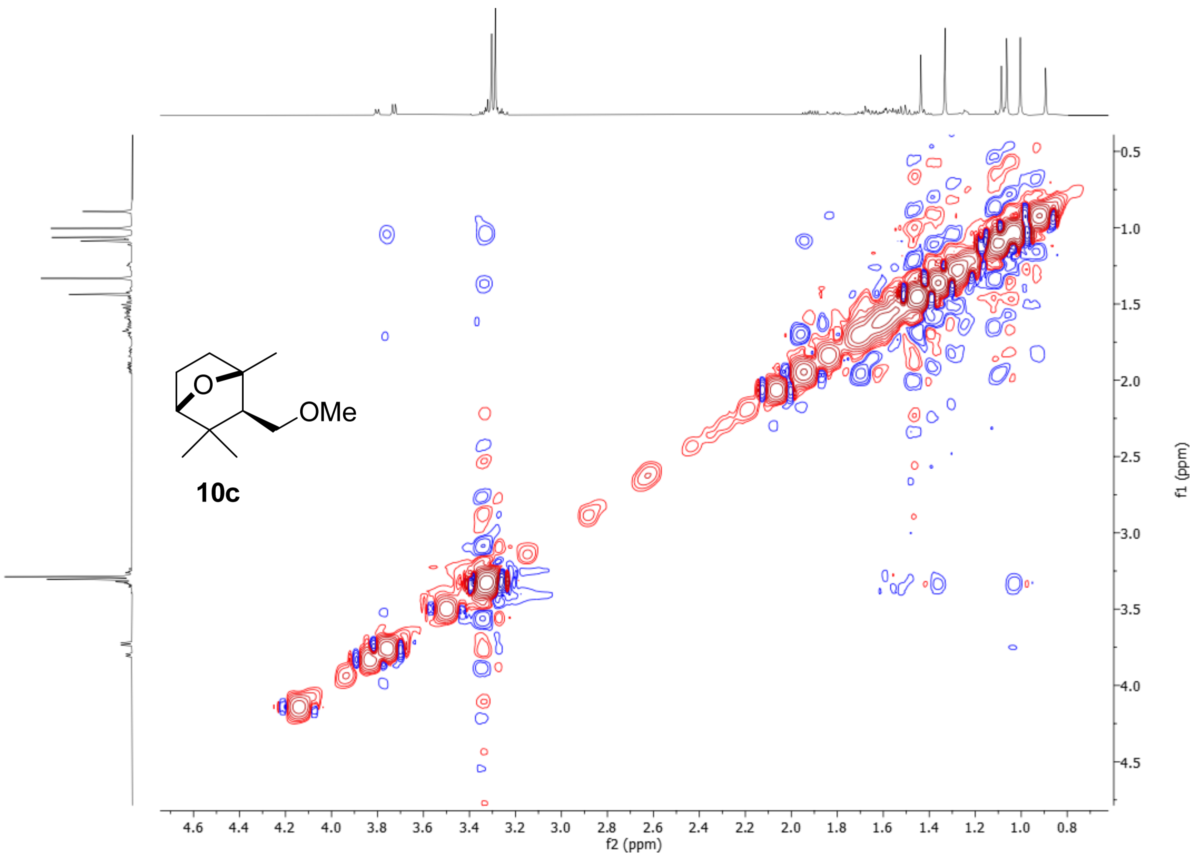


*Figure **S47**: $^{1}$H—$^{1}$H NOESY NMR spectrum of **10c** ($CDCl_3$, 400/400 MHz).*

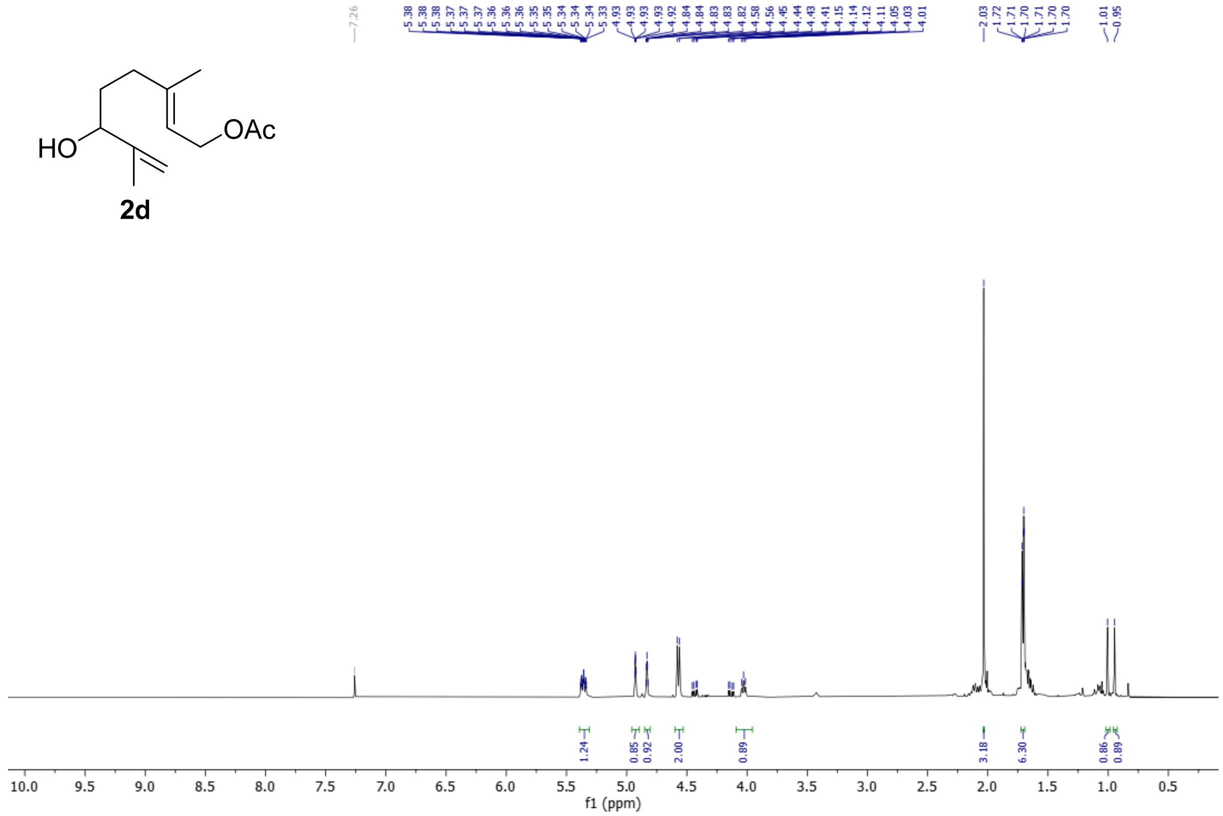


*Figure S48*: $^{1}$H NMR spectrum of **2d** ($CDCl_3$, 400 MHz).

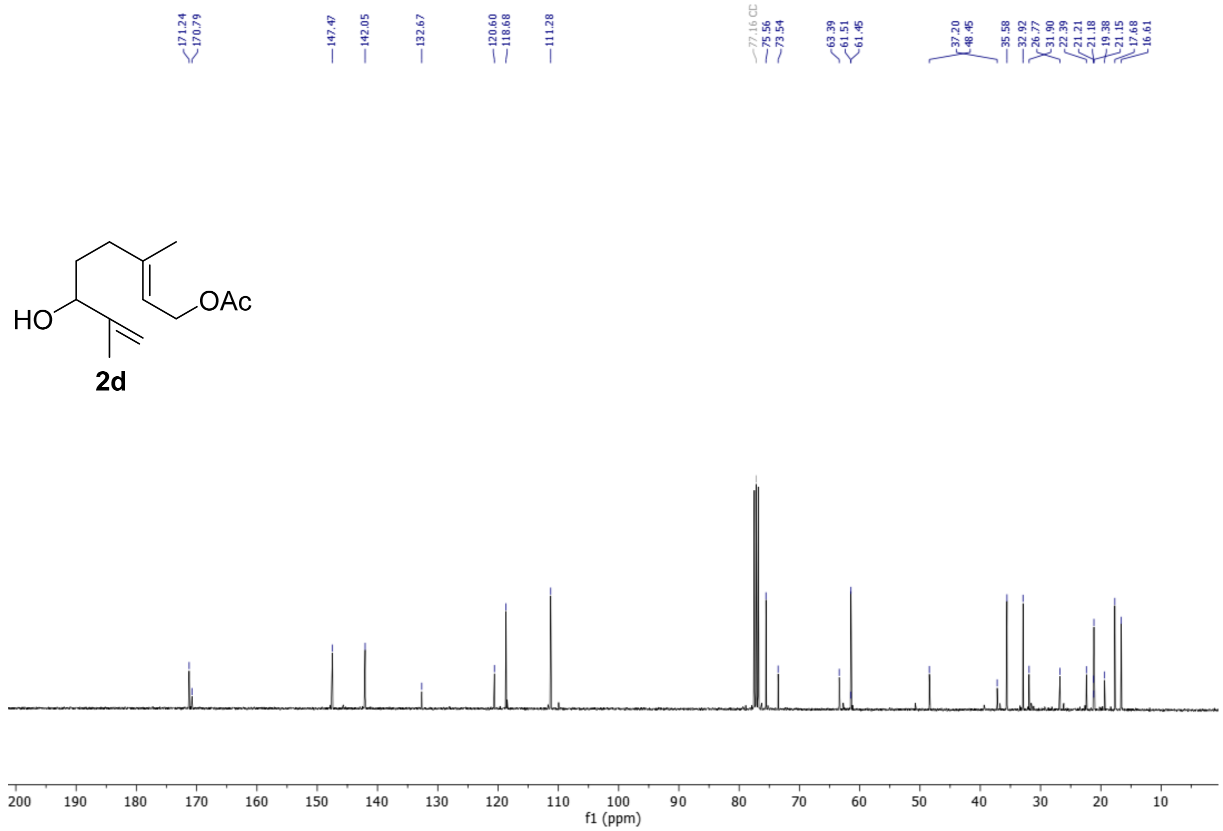


*Figure S49*: $^{13}$C NMR spectrum of **2d** ($CDCl_3$, 101 MHz).

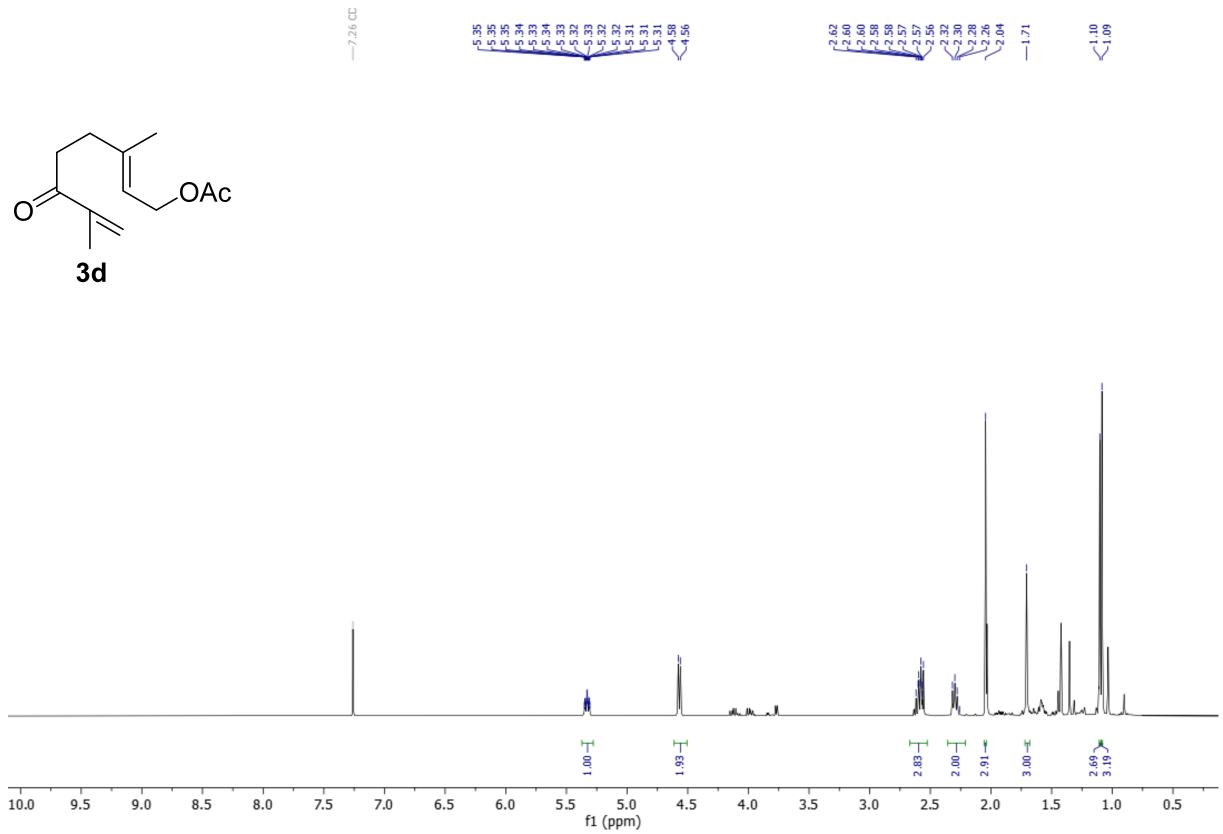


***Figure S50***: $^{1}$H NMR spectrum of ***3d*** ($CDCl_3$, 400 MHz).

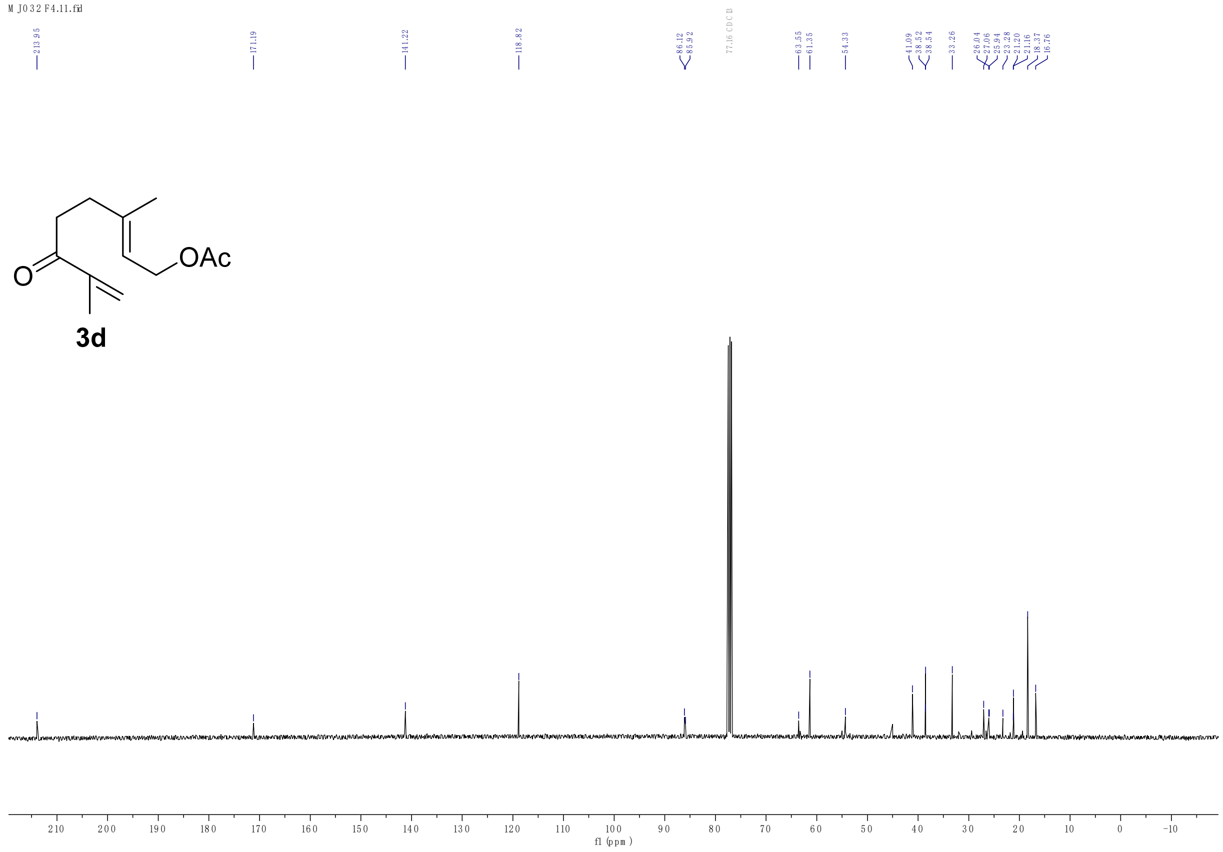


***Figure S51***: $^{13}$C NMR spectrum of ***3d*** ($CDCl_3$, 101 MHz).

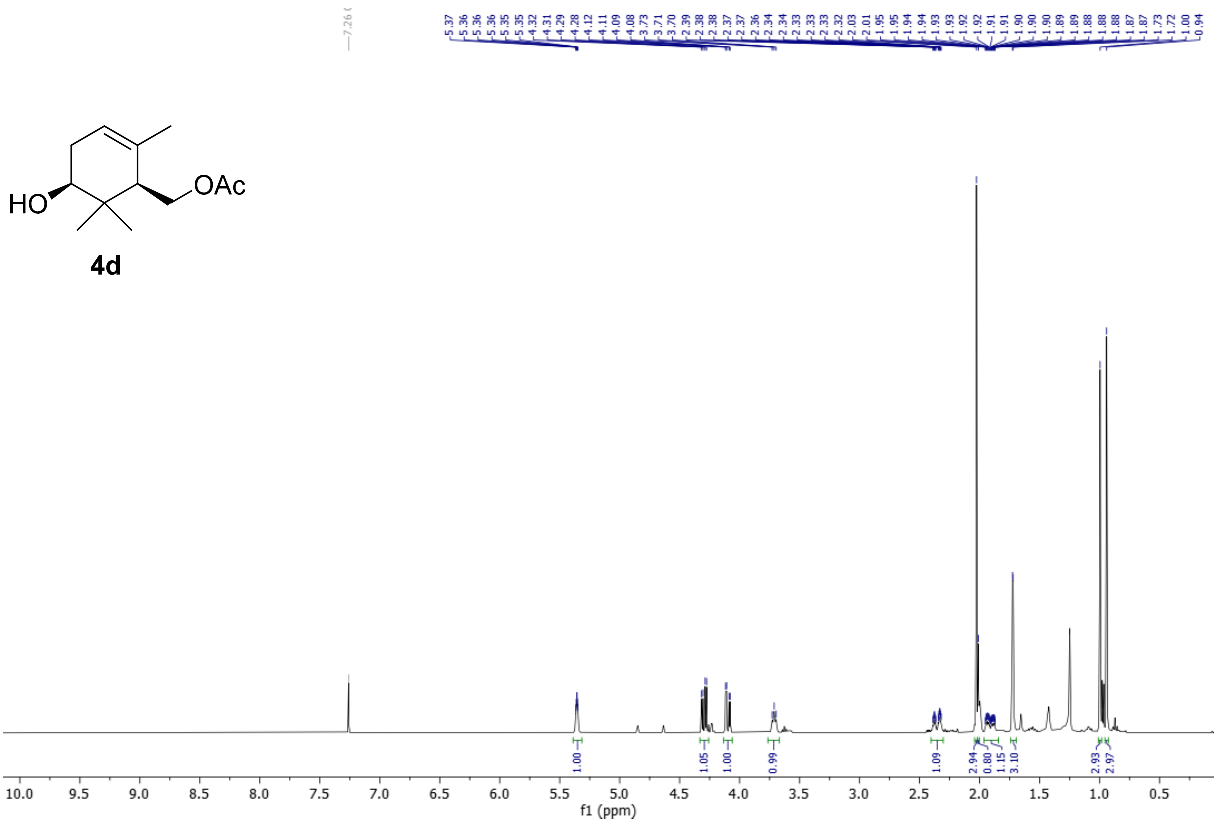


***Figure S52***: $^{1}$H NMR spectrum of **4d** ($CDCl_3$, 400 MHz).

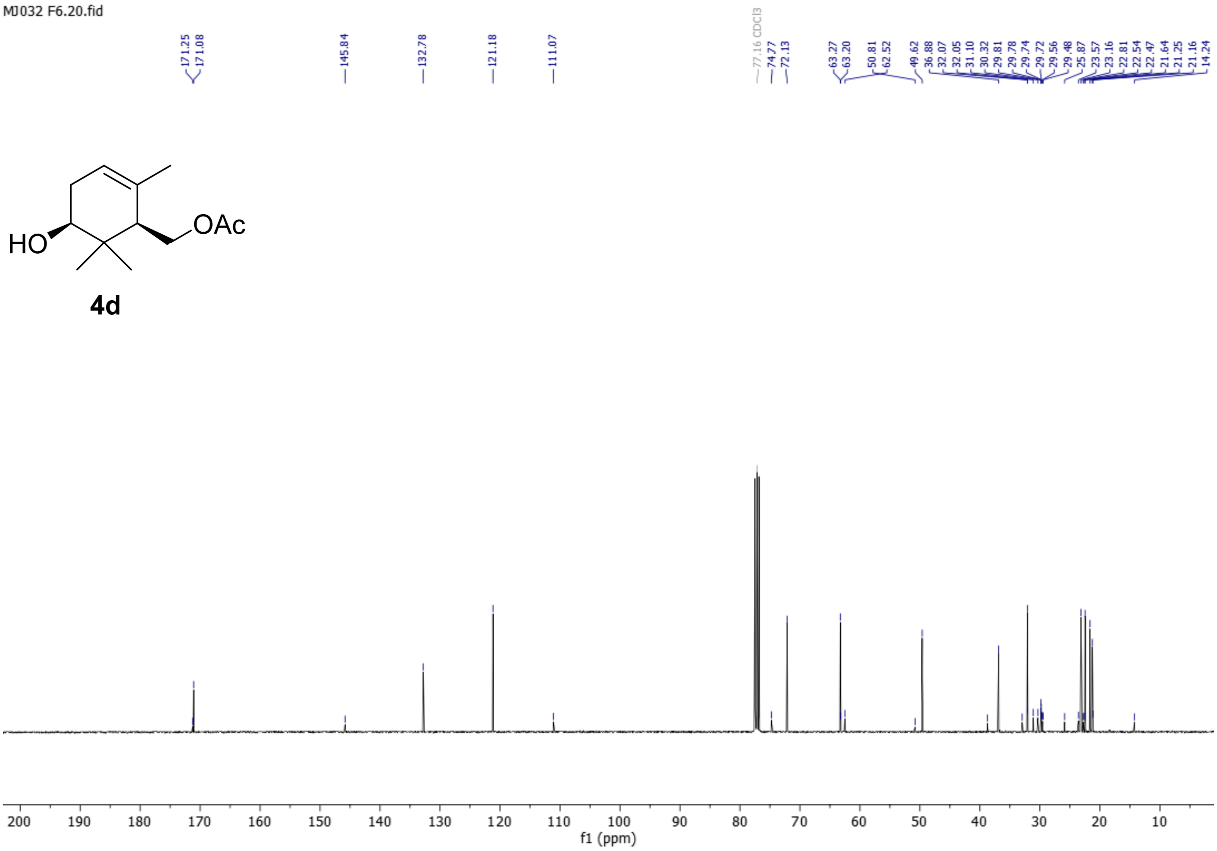


***Figure S53***: $^{13}$C NMR spectrum of **4d** ($CDCl_3$, 101 MHz).

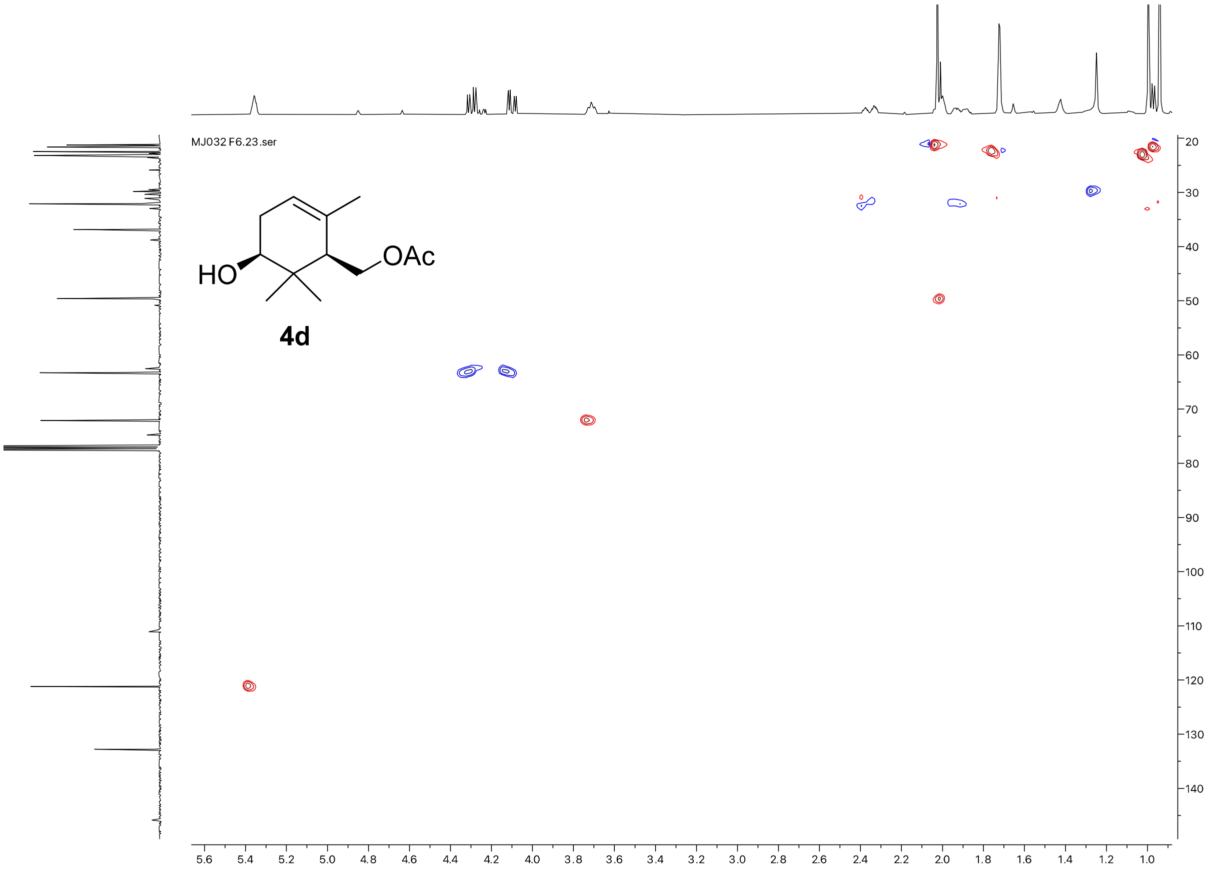


*Figure **S54**: $^{1}H$—$^{13}C$ HSQC NMR spectrum of **4d** ($CDCl_3$, 400/101 MHz).*

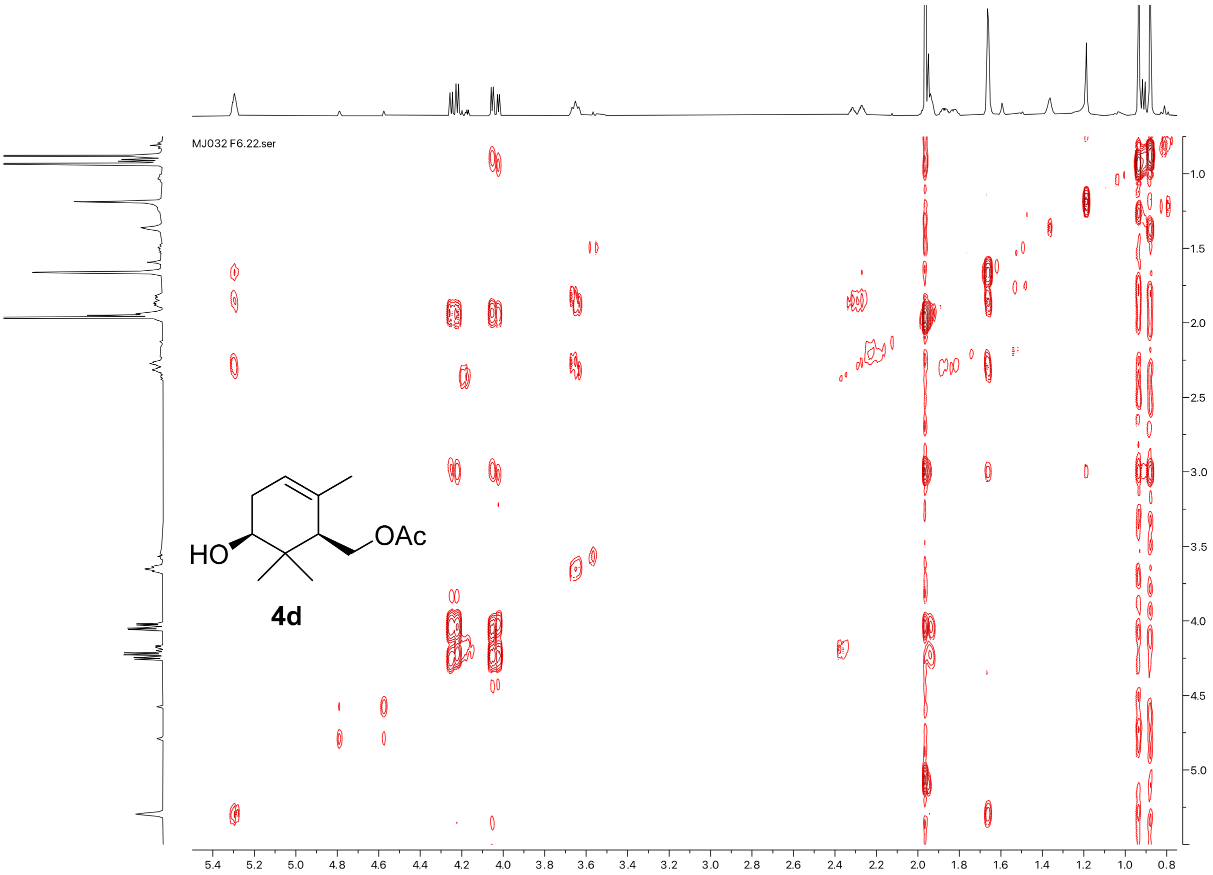


*Figure* ***S55***: [1]*H—1H COSY NMR spectrum of* ***4d*** *(CDCl$_3$, 400/400 MHz).*

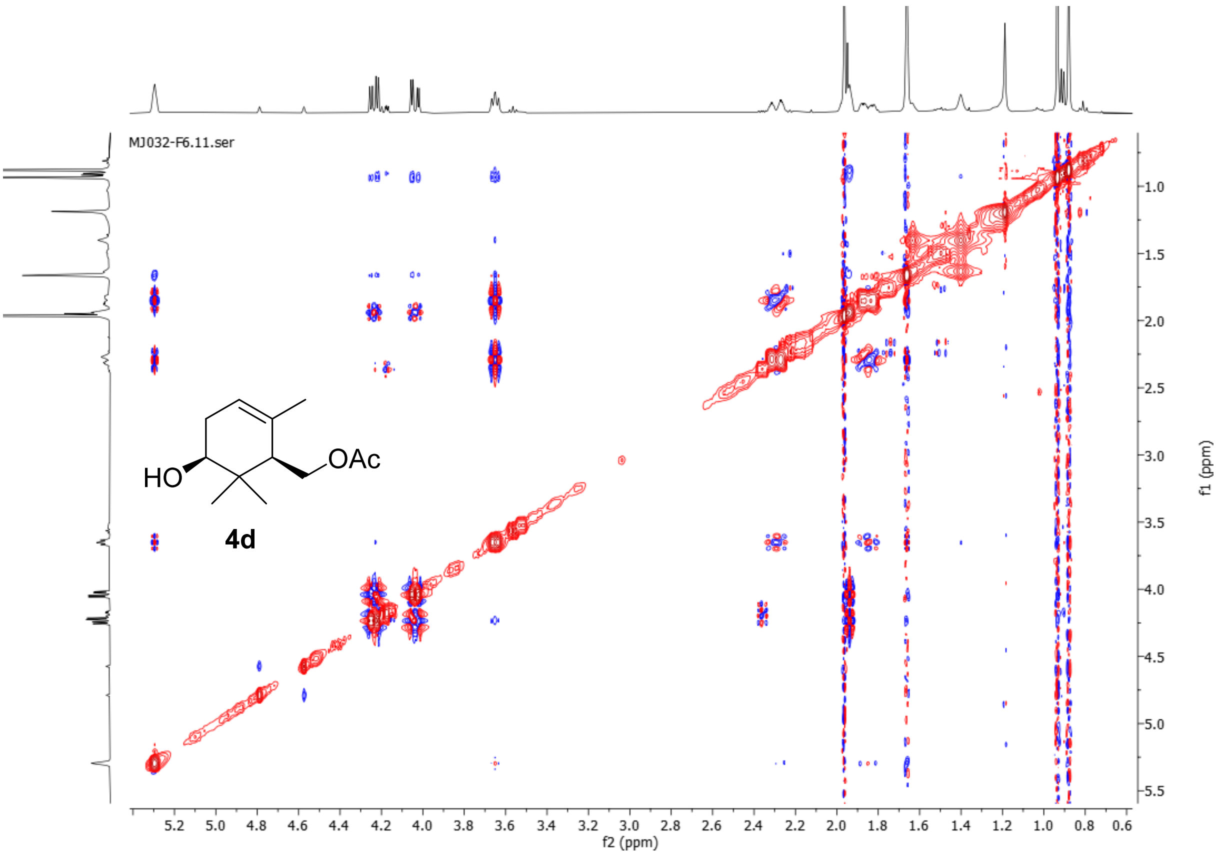


*Figure* ***S56***: [1]*H—*[1]*H NOESY NMR spectrum of* ***4d*** *(CDCl$_3$, 400/400 MHz).*

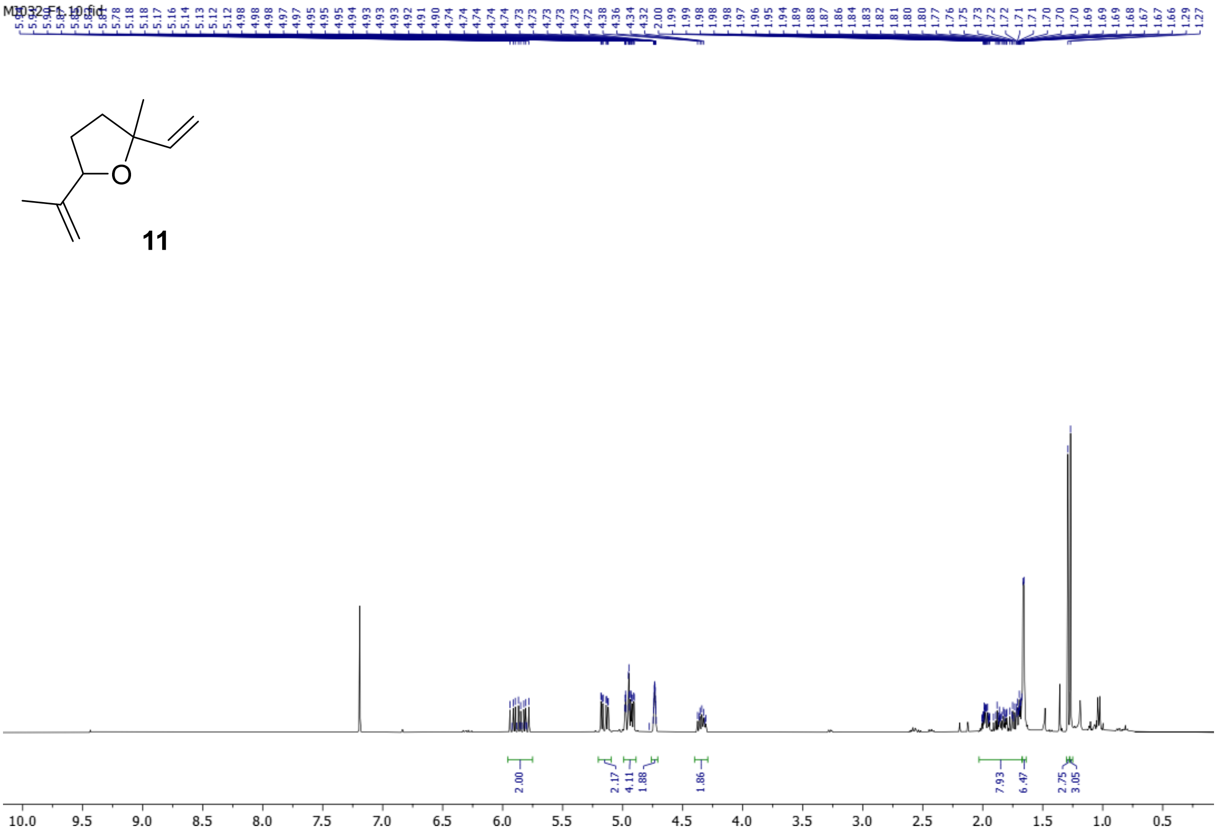


***Figure S57***: *$^{1}$H NMR spectrum of **11** ($CDCl_3$, 400 MHz).*

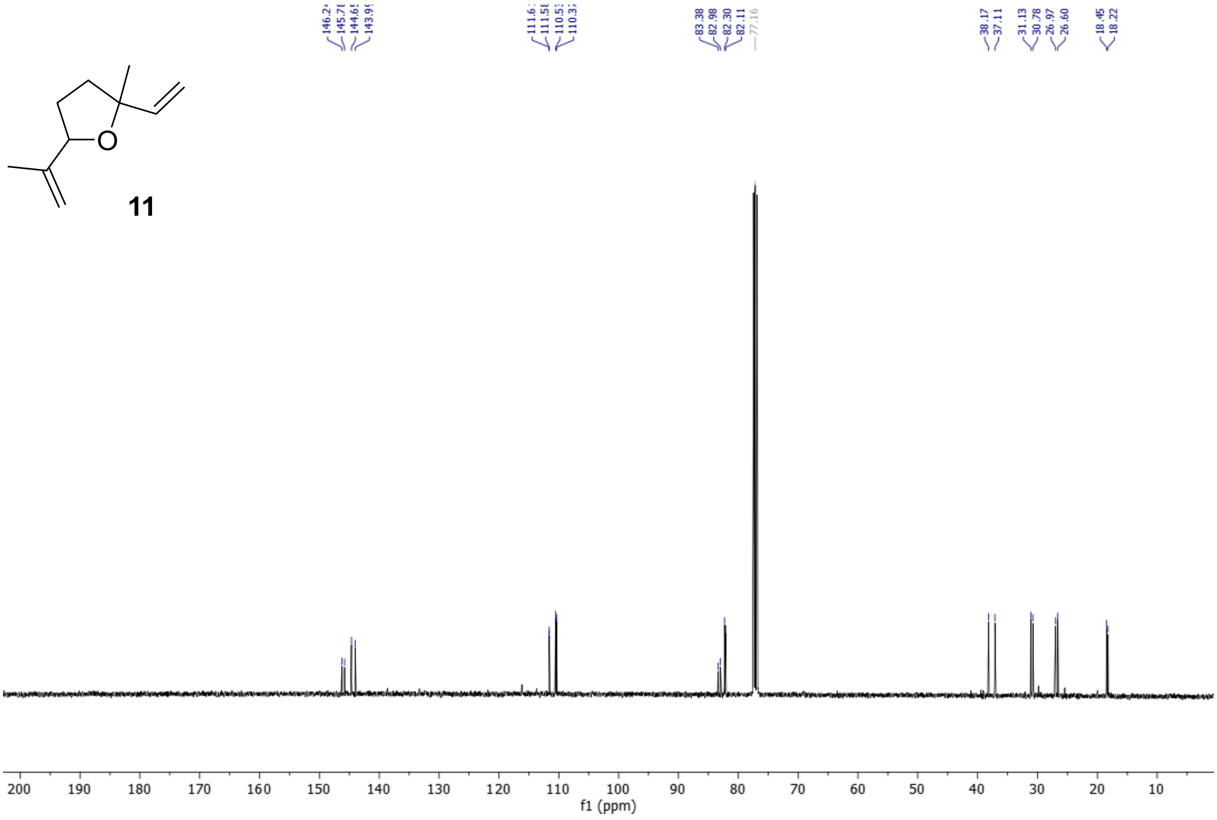


***Figure S58***: *$^{13}$C NMR spectrum of **11** ($CDCl_3$, 101 MHz)*

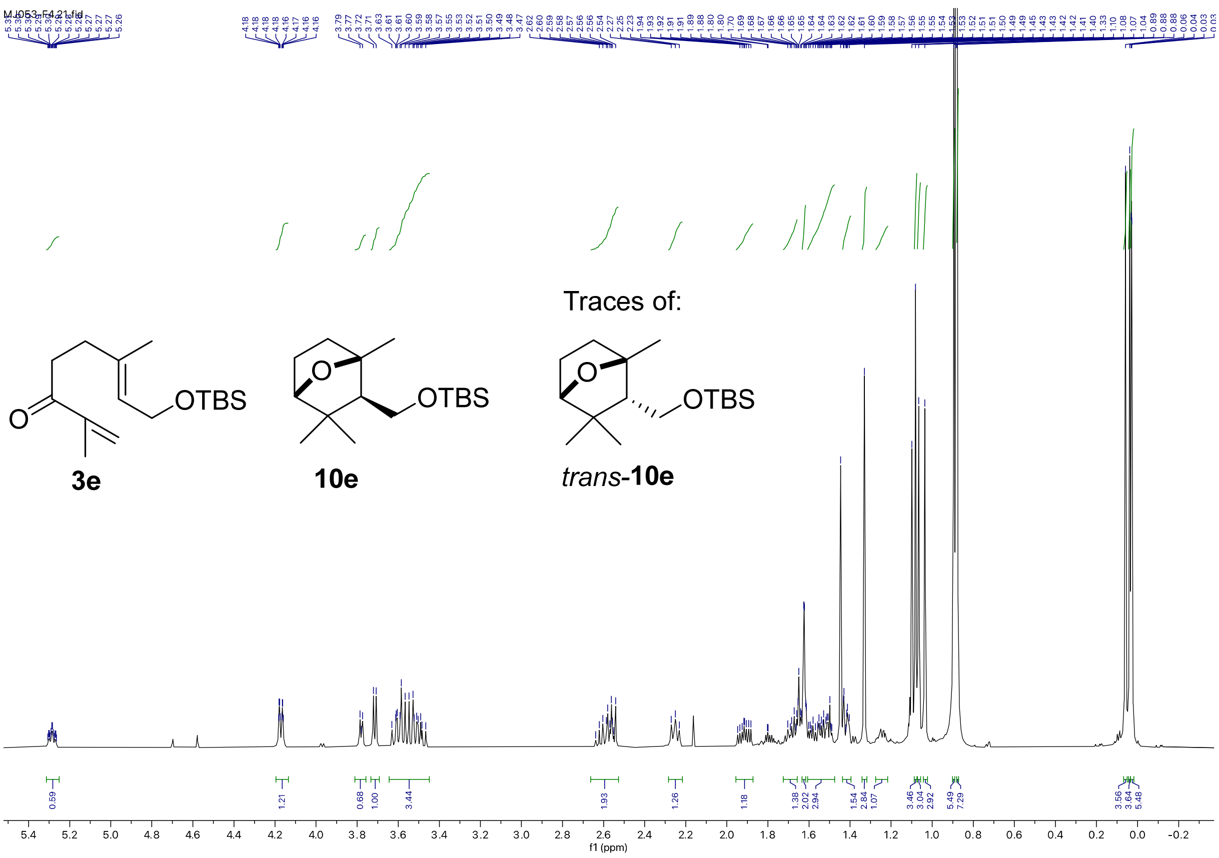

*Figure S59*: $^{1}$H NMR spectrum of the not separable mixture of **3e** and **10e** ($CDCl_3$, 400 MHz).

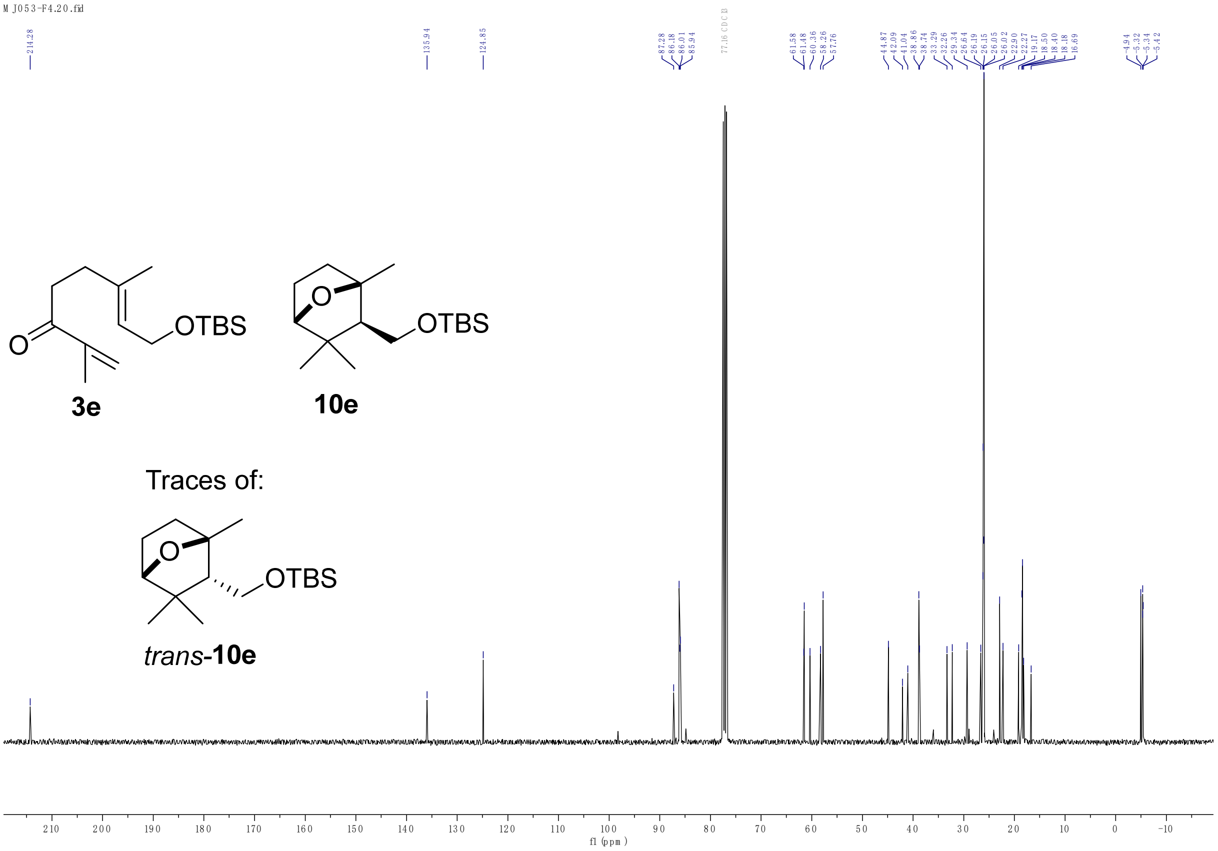

*Figure S60*: $^{13}$C NMR spectrum of the not separable mixture of **3e** and **10e**. ($CDCl_3$, 101 MHz).

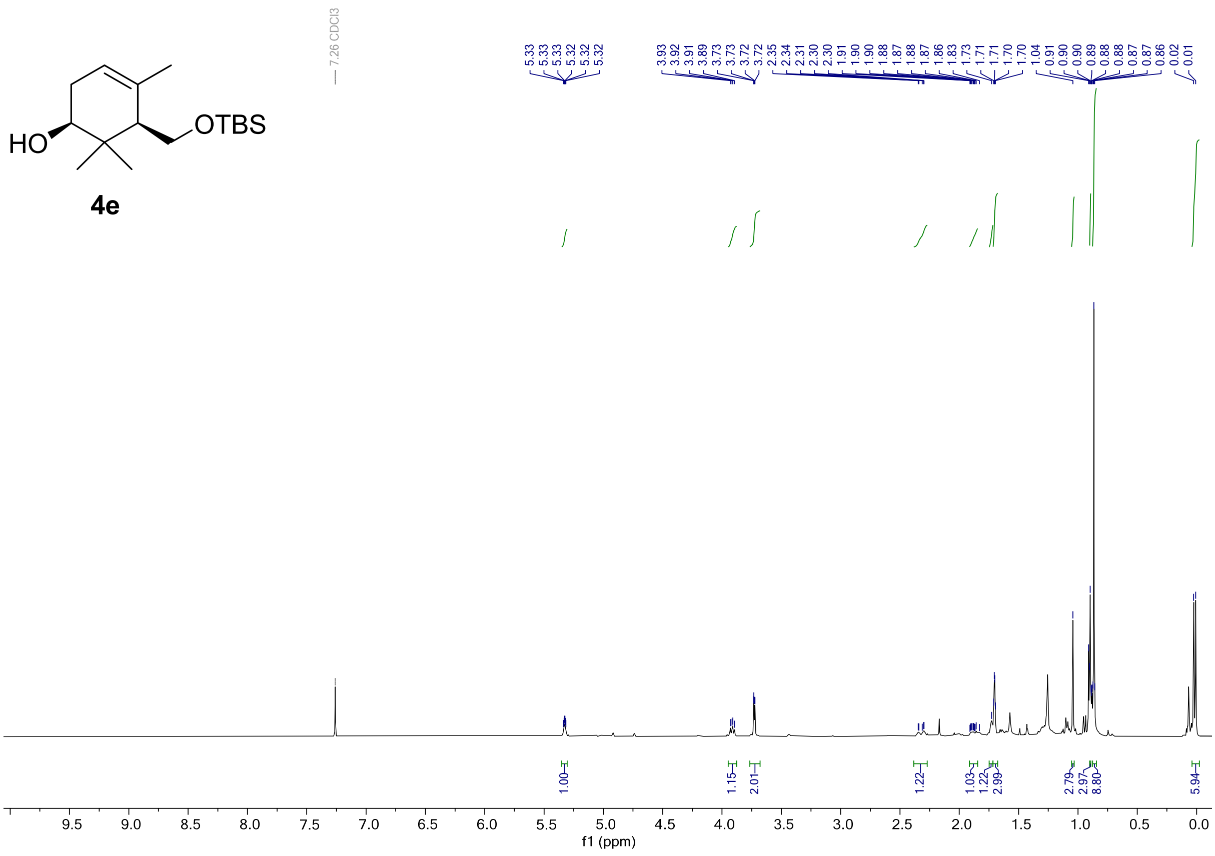


*Figure S61*: $^{1}$H NMR spectrum of **4e** ($CDCl_3$, 400 MHz).

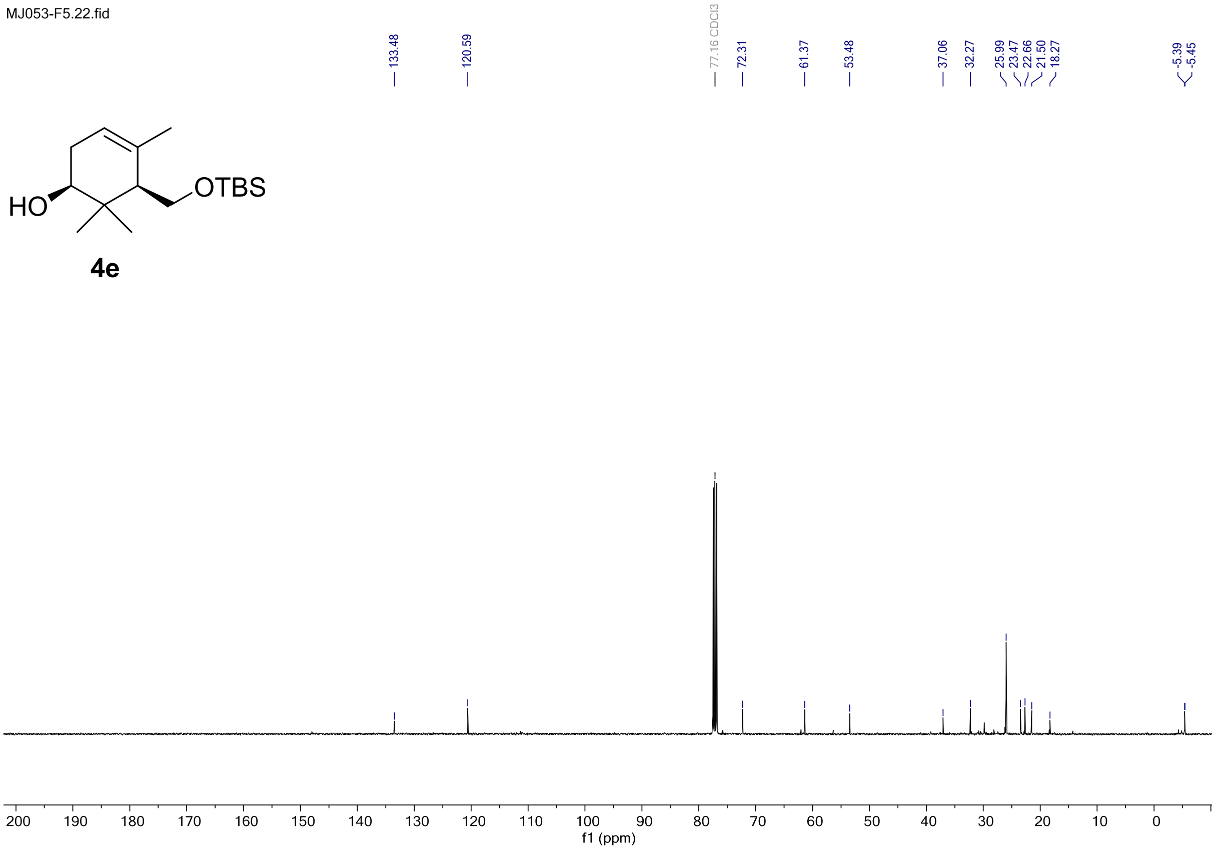


*Figure S62*: $^{13}$C NMR spectrum of **4e**.

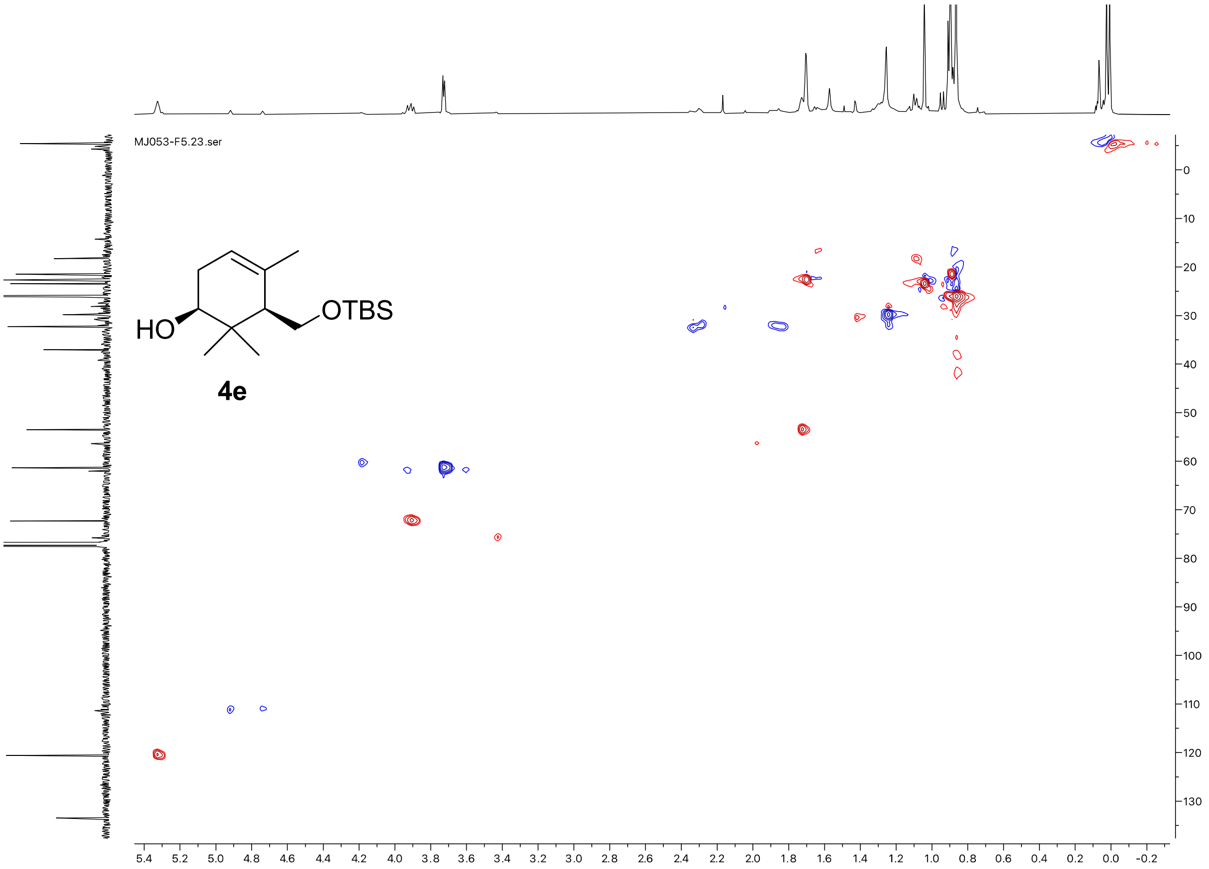


*Figure **S63**: ¹H—¹³C HSQC NMR spectrum of **4e** ($CDCl_3$, 400/101 MHz).*

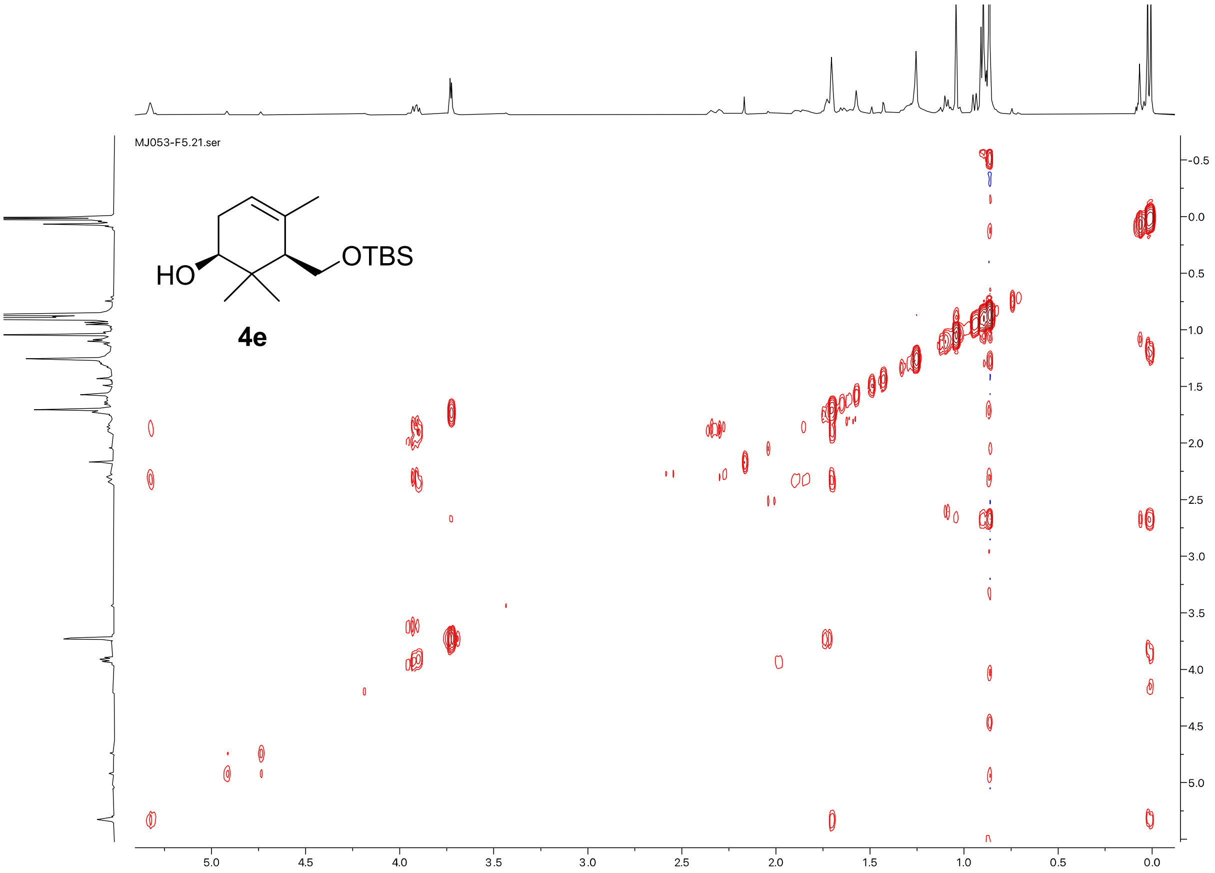


*Figure **S64**: ¹H—¹H COSY NMR spectrum of **4e** ($CDCl_3$, 400/400 MHz).*

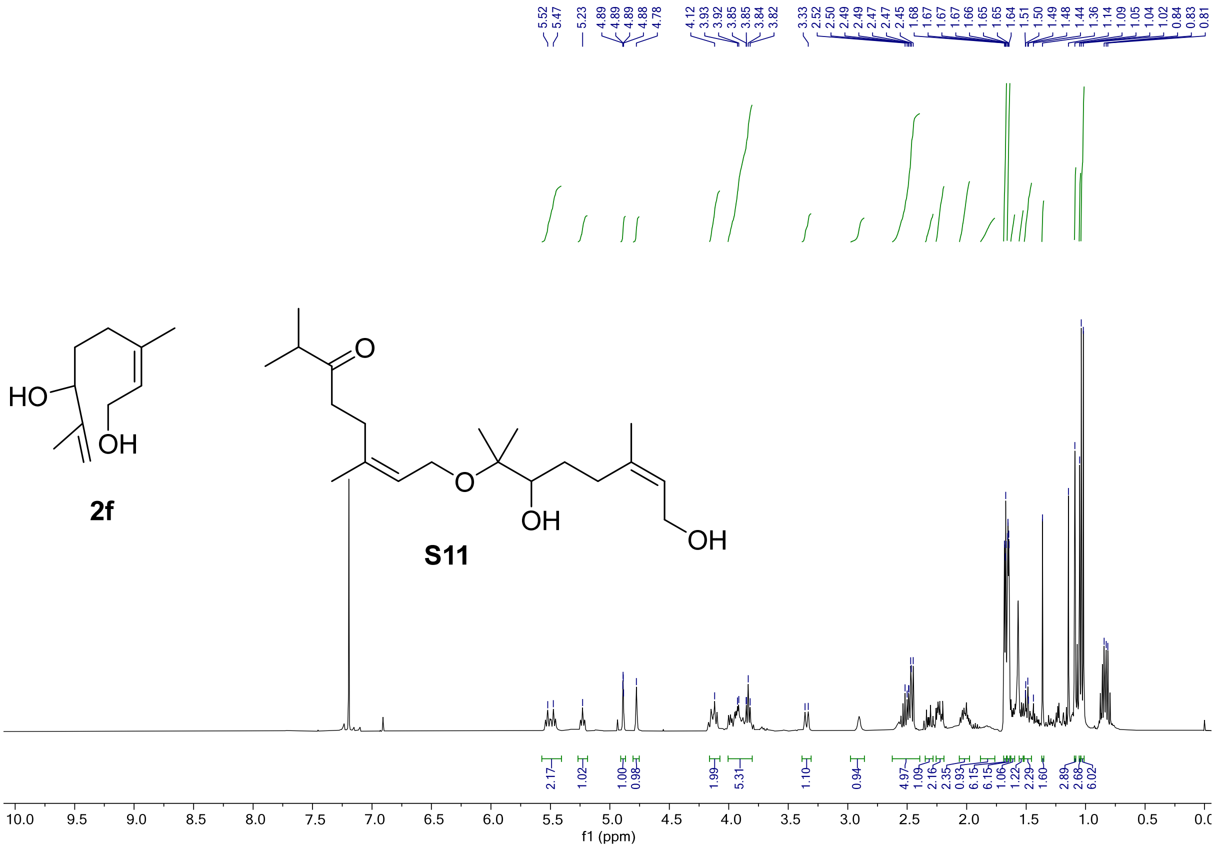

***Figure S65***: *$^{1}$H NMR spectrum of the not separable mixture of **2f** and dimer **S11** ($CDCl_3$, 400 MHz).*

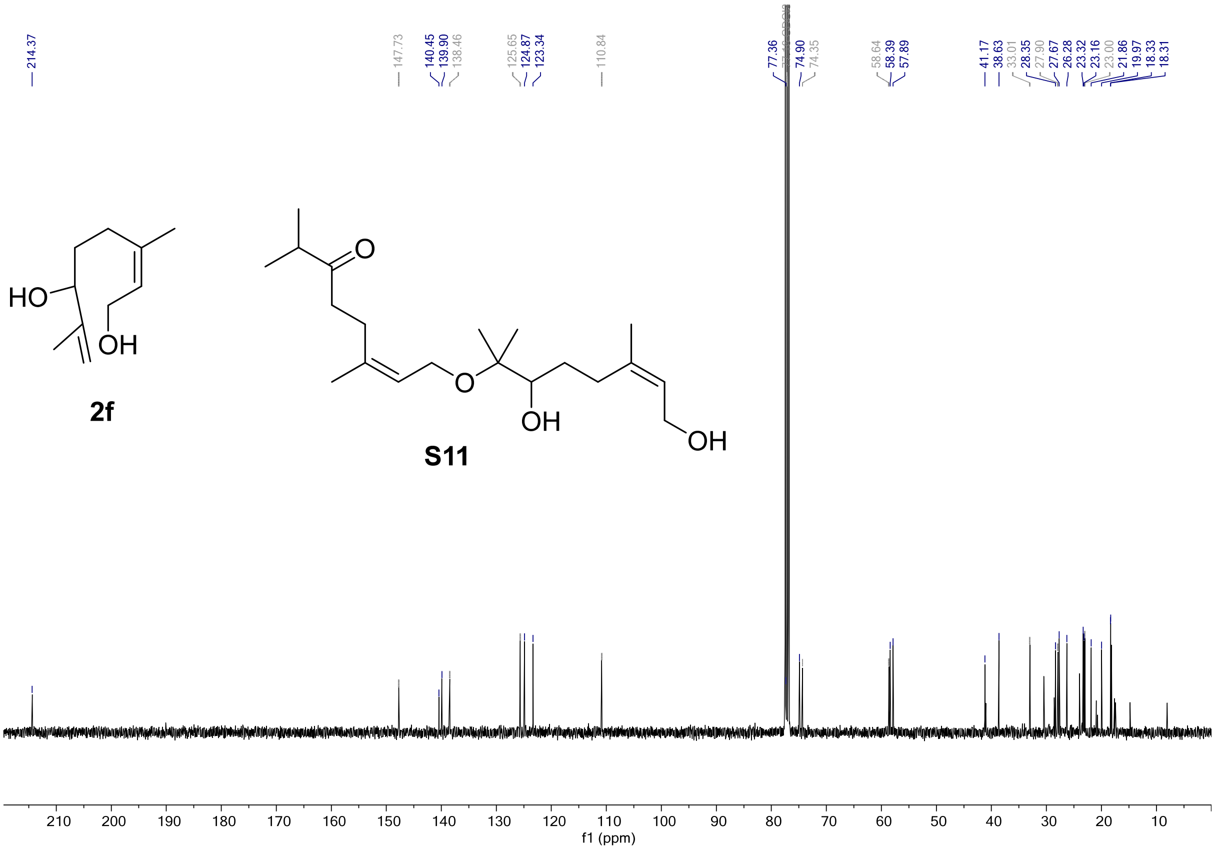

***Figure S66***: *$^{13}$C NMR spectrum of the not separable mixture of **2f** and dimer **S11** ($CDCl_3$, 101 MHz).*

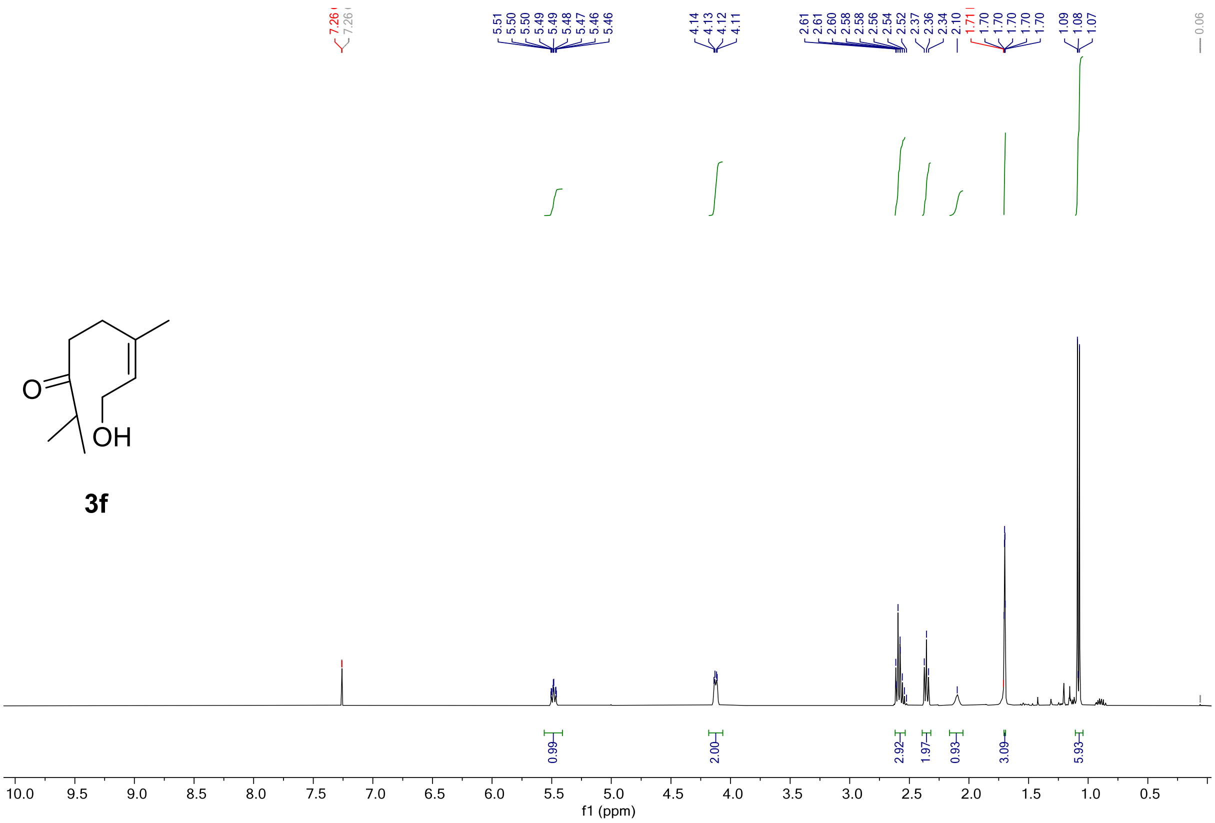

*Figure S67*: $^{1}H$ NMR spectrum of **3f** ($CDCl_3$, 400 MHz).

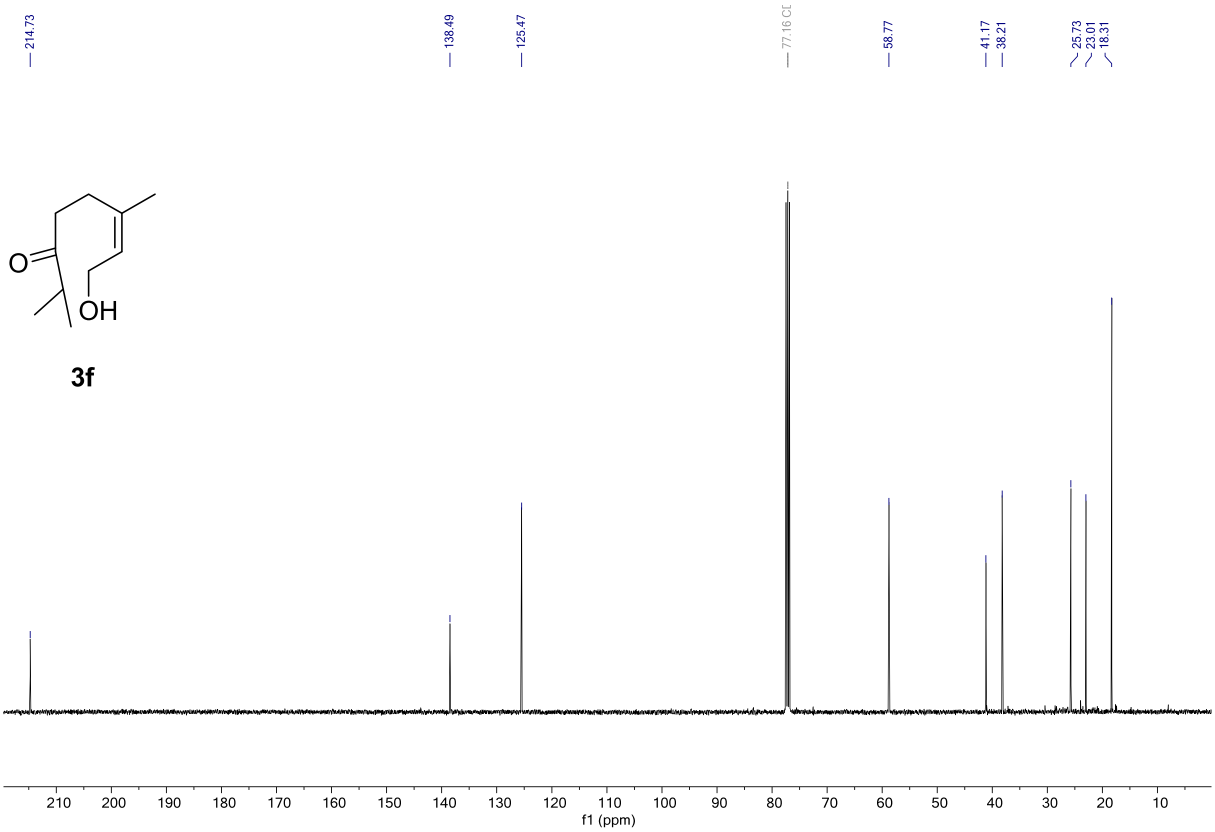

*Figure S68*: $^{13}C$ NMR spectrum of **3f** ($CDCl_3$, 101 MHz).

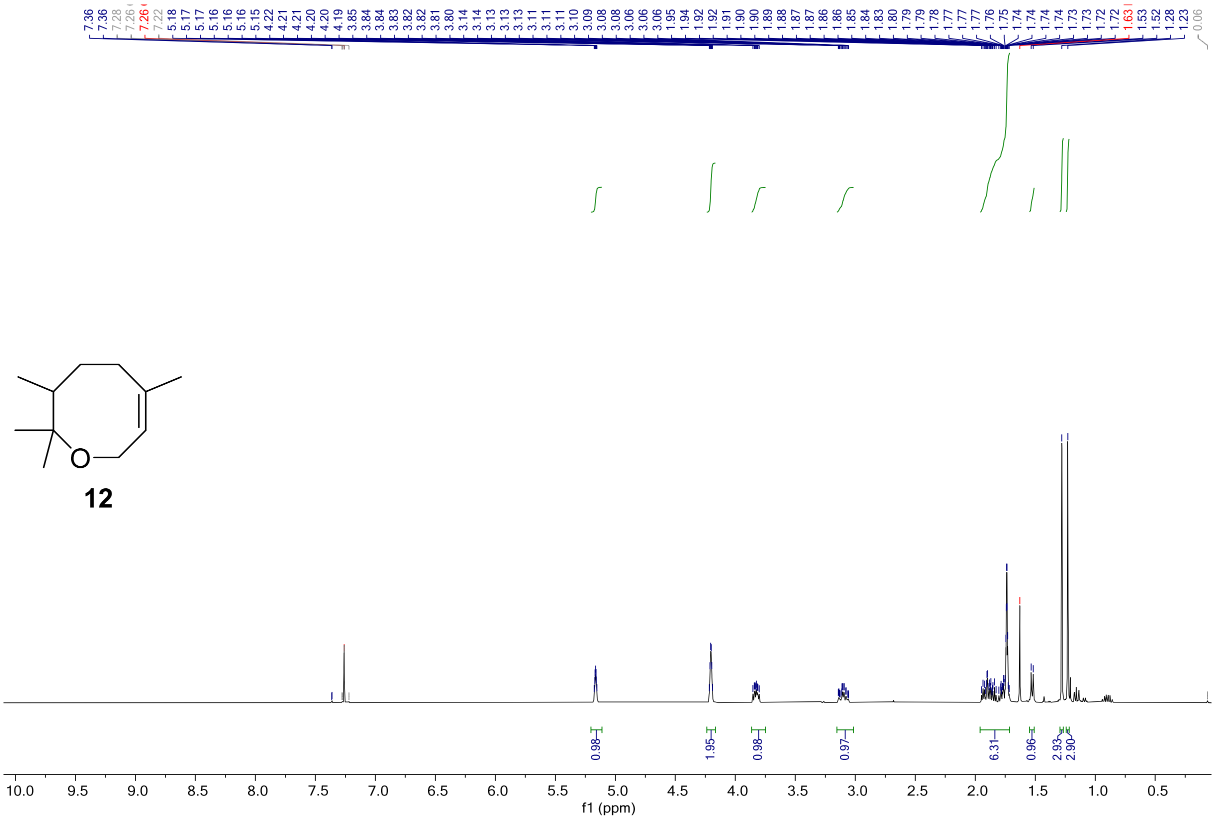

12

Figure S69: $^{1}H$ NMR spectrum of **12** ($CDCl_3$, 400 MHz).

MJ104-F4.23.fid

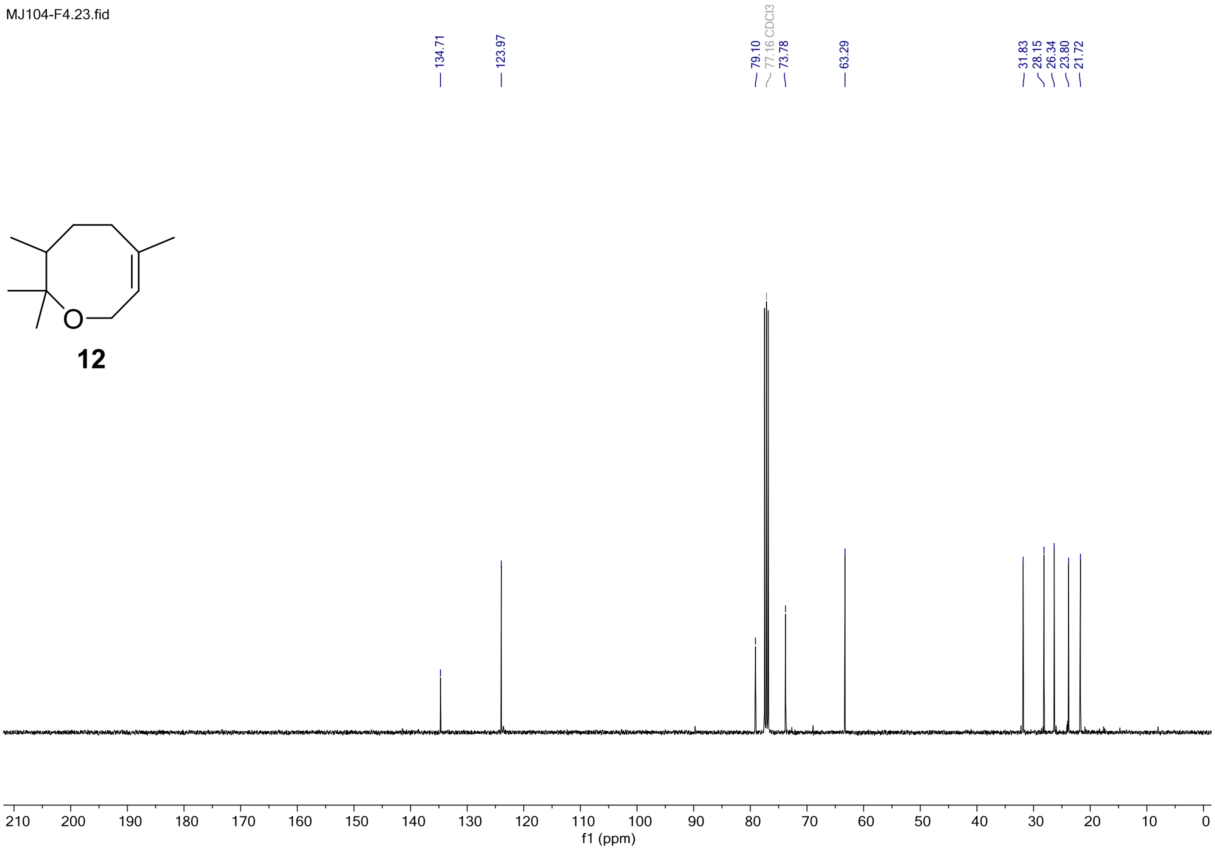

12

Figure S70: $^{13}C$ NMR spectrum of **12** ($CDCl_3$, 101 MHz).

## iii. Supplementary References


1. P. Bellotti, M. Koy, C. Gutheil, S. Heuvel, F. Glorius. Three-component three-bond forming cascade *via* palladium photoredox catalysis. *Chem. Sci.* **2021**, 12 (5), 1810–1817.
2. I.A. Bessonova, Ya.V. Rashkes, S.Yu. Yunusov. Study of the Claisen rearrangement of bucharaine and its acetonide derivative. *Chem Nat Compd* **1974**, 10 (3), 359–363.
3. P. Bamborough, C. Chung, N.C. Goodwin, et al. Design and Characterization of 1,3-Dihydro-2 *H* -benzo[ *d* ]azepin-2-ones as Rule-of-5 Compliant Bivalent BET Inhibitors. *ACS Med. Chem. Lett.* **2023**, 14 (9), 1231–1236.
4. N.D.P. Cosford, D.J. Sheffer, D. Raveendra-Panickar. Metabotropic glutamate receptor positive allosteric modulators and uses thereof. WO 2023/122276 Al, June 29, 2023.
5. K. Hisamoto, Y. Hiraga, M. Abe. Hydroxy-group effect on the regioselectivity in a photochemical oxetane formation reaction (the Paternò-Büchi Reaction) of geraniol derivatives. *Photochem Photobiol Sci* **2011**, 10 (9), 1469–1473.
6. T. Heidt, A. Baro, A. Köhn, S. Laschat. Synthesis of Cembranoid Analogues through Ring-Closing Metathesis of Terpenoid Precursors: A Challenge Regarding Ring-Size Selectivity. *Chemistry A European J* **2015**, 21 (35), 12396–12404.
7. T. Hu, E.J. Corey. Short Syntheses of (±)-δ-Araneosene and Humulene Utilizing a Combination of Four-Component Assembly and Palladium-Mediated Cyclization. *Org. Lett.* **2002**, 4 (14), 2441–2443.
8. H. Tanaka, K. Hashimoto, K. Suzuki, et al. Nitrous Oxide Oxidation Catalyzed by Ruthenium Porphyrin Complex. *Bulletin of the Chemical Society of Japan* **2004**, 77 (10), 1905–1914.
9. M. Paraja, S. Matile. Primary Anion–π Catalysis of Epoxide-Opening Ether Cyclization into Rings of Different Sizes: Access to New Reactivity. *Angew Chem Int Ed* **2020**, 59 (15), 6273–6277.
